# Intensive oral prophylaxis does not alter the tongue microbiome in young patients with chronic kidney disease: longitudinal, randomized, controlled study

**DOI:** 10.3389/fimmu.2024.1430655

**Published:** 2024-08-19

**Authors:** Karolin C. Hoefer, Lutz T. Weber, Anna Greta Barbe, Isabelle Graf, Stefanie Thom, Rasmus Ehren, Angela Nowag, Hilmar Wisplinghoff, Michael J. Noack, Claus J. Scholz, Nathalie Jazmati

**Affiliations:** ^1^ Faculty of Medicine and University Hospital of Cologne, Polyclinic of Operative Dentistry and Periodontology, University of Cologne, Cologne, Germany; ^2^ Department of Pediatrics, Faculty of Medicine and University Hospital of Cologne, Children’s and Adolescents Hospital, Pediatric Nephrology, University Hospital of Cologne, Cologne, Germany; ^3^ Department of Orthodontics, Faculty of Medicine and University Hospital Cologne, Cologne, Germany; ^4^ Wisplinghoff Laboratories, Cologne, Germany; ^5^ Institute for Virology and Microbiology, Witten/Herdecke University, Witten, Germany; ^6^ Institute for Medical Microbiology, Immunology and Hygiene, University of Cologne, Cologne, Germany

**Keywords:** microbiome, tongue, dental prophylaxis, chronic kidney disease, oral health, child, adolescents

## Abstract

**Introduction:**

Gingivitis is a common intraoral disease in patients with chronic kidney disease (CKD), which poses a particular interdisciplinary challenge. We aimed to determine the influence of an intensive oral prophylaxis program (OPP) compared to standard prevention measures on the tongue microbiome of young patients with CKD.

**Methods:**

Thirty patients with CKD (mean age 14.2 ± 5.2 years) and generalized gingivitis were included. The effects of the intensive OPP were compared with standard prophylaxis according to statutory health insurance (treatment as usual, TAU) as a control. Tongue swabs were taken from the patients at baseline (t1) and after 3 (t2) and 6 (t3) months. Next-generation sequencing of 16S rDNA genes was used to quantitatively characterize microbial communities.

**Results:**

There were no differences in the abundance, richness, or diversity of the observed genera and species between the two study groups at baseline or after 3 or 6 months. Furthermore, no change in predefined gingivitis and oral health bacterial clusters were found. At the phylum level, Firmicutes were decreased after intervention in the TAU group (t2_TAU_ 42.9 ± 7.1 to t3_TAU_ 34.8 ± 4.7 (n_pairs_=14), p=0.003; false discovery rate 0.02). The decrease of Firmicutes was not significant in the OPP group.

**Conclusions:**

Despite the intensity of dental prophylaxis and decreasing clinical signs of inflammation and decreasing plaque amount, no clinically relevant changes in the tongue microbiome were observed. Our results confirm the conserved and stable nature of the tongue microbiome, even in children with CKD.

## 1 Introduction

Chronic kidney disease (CKD) in children is mostly due to congenital abnormalities of the kidney and urinary tract, as well as hereditary diseases. Pediatric CKD is associated with high morbidity and even mortality, especially in children requiring dialysis (1, 2). In children under the age of 12 years, the survival rate three years after starting dialysis is 95%; in children aged 4 years or younger, it is around 82% after one year (3). In Europe, approximately 11-12 per million children and adolescents develop stages 3-5 of chronic kidney disease annually, with a prevalence estimated at 60-70 per million in the age-related population (4). Despite extensive progress in the treatment of patients affected by chronic kidney disease (CKD), morbidity and mortality still remain unacceptably high (5). One of the major causes of death are cardiovascular diseases (CVD) (6, 7). Various factors contribute to the chronic inflammatory status observed in CKD, including increased production and decreased clearance of proinflammatory cytokines, oxidative stress and acidosis, chronic and recurrent infections, altered metabolism of adipose tissue, and intestinal dysbiosis (8). Chronic kidney disease (CKD) in adults is linked to dysbiosis of the human intestinal microbiome. This imbalance may be attributed to CKD-related factors such as uremia, heightened inflammation, immunosuppression, pharmacological treatments, and dietary restrictions (9). Moreover, a variety of treatments for patients with CKD, including hemodialysis and peritoneal dialysis, can be associated with alterations in the intestinal microbiome (9). Among various factors, poor oral health in CKD patients is a significant yet often underestimated source of chronic inflammation (10, 11). Young patients diagnosed with CKD often exhibit generalized gingivitis, increased plaque accumulation, attachment loss, enamel defects, and gingival hyperplasia (12). Gingivitis in general is characterized by gingival inflammation in response to dental plaque accumulation, which can be restored to health with oral hygiene measures that reduce the bacterial burden. The pathogenesis of gingivitis appears to be multifactorial, involving environmental factors and the oral microbiota (13), and has been linked to chronic systemic inflammation—thereby posing additional challenges to individuals with CKD (11, 14). The role of the oral microbiome in this pathogenesis is not fully understood. Limited data exist on the oral microbiome of children, adolescents, and young adults with chronic kidney disease (CKD). Additionally, a comparable study examining the relationship between CKD and the oral microbiome in adult patients utilized pharyngeal swabs (15). According to Guo et al., patients with chronic kidney disease (CKD) exhibit higher microbial diversity compared to healthy controls. This result implies that potential oral microbial markers could serve as non-invasive diagnostic tools for CKD (15). The tongue, as an integral component of the oral microbiome, has been identified as a reservoir for periodontal disease-related bacterial species (16, 17). Understanding the dynamics of the tongue microbiome is crucial for addressing the potential impact on preventive dental care. A recent cross-sectional study by Hoefer et al. confirmed that the tongue microbiome of young CKD patients showed no significant differences compared to their healthy mothers, suggesting its stability as a niche within the oral cavity (18). However, there are well-described gingivitis and oral health clusters that summarize bacterial species associated with gingivitis and oral health, respectively (19). The potential alterability of these clusters within the resilient tongue microbiome through intensive dental prophylaxis interventions, and any subsequent influence on the disease, remain open questions. Overall, the treatment of gingivitis is an essential prevention strategy for periodontitis (20, 21). Periodontitis may elevate the systemic inflammatory burden in individuals with CKD, potentially contributing to increased mortality rates in adults (22).

The primary objective of our study was to investigate whether preventive dental care measures of varying intensity have a discernible influence on the tongue microbiome, specifically targeting gingivitis pathogens in CKD patients. We aimed to contribute to a deeper understanding of the interactions between oral health interventions and the tongue microbiome in the context of CKD, potentially informing more effective strategies for preventive dental care in this vulnerable patient population.

## 2 Materials and methods

### 2.1 Study design

Our tongue microbiome analysis was part of a clinical trial that was performed as a prospective, single-center, randomized controlled clinical trial (23). Patients represented a typical study population from the Department of Pediatric Nephrology at the University Hospital of Cologne, Germany. The effects of an intensive oral prophylaxis program (OPP) were compared with standard statutory health insurance prophylaxis (treatment as usual, TAU) (see details below).

Patients who attended their CKD control assessment (Aug 1, 2016, to Aug 31, 2019) were consecutively included in the study. All patients were initially enrolled by a pediatric nephrologist and examined by the dentists involved in the study. Patients were enrolled at baseline for dental screening and randomization (t1), as described in Höfer et al. (23). The study arm received OPP in the following weeks, while the control arm received TAU. All patients were examined again after termination of OPP/TAU (10 ± 1 weeks after randomization; t2) and at 12 weeks after t2 (t3). The control group received a single intensive prophylaxis treatment between t2 and t3.

According to the study protocol, tongue swabs were taken at all three time points. Tongue swabs were obtained in the morning (all patients fasted until sample collection), and patients were asked to refrain from brushing their teeth or using mouthwashes for 12 h before the sample was taken. Oral microbiome samples were collected at all three study time points from the posterior tongue dorsum using dry cotton swabs (Microbrush, Germany), transferred to sterile 1.5-ml reaction tubes (Eppendorf, Germany), and stored at -80°C until use for 16S rDNA sequencing.

The trial was approved by the Ethics Committee of the Faculty of Medicine, University of Cologne, Germany, and recorded at The German Clinical Trials Register (registration number DRKS00010580).

### 2.2 Inclusion and exclusion criteria

Patients who regularly attended the Department of Pediatric Nephrology at the University Hospital for examination were screened by pediatric nephrologists according to the following criteria (see also (18)). Patients with CKD grades 1 to 5 (according to the Kidney Disease Improving Global Outcomes (KDIGO) classification (24)) who were conservatively treated, underwent transplantation or dialysis, and were affected by gingivitis (gingival index (GI) >0 and periodontal screening index (PSI) 0–2, determined by the study dentist) were included. The exclusion criteria were any signs of acute infection, fever, or antibiotic treatment 14 days prior to participation. This decision was made by pediatric nephrologists based on the clinical parameters and blood tests. Patients who did not have tongue swabs taken at the time points described above were also excluded from the analysis. Prior to participation in the study, written informed consent was obtained from the parents/legal guardians of eligible young patients and, if indicated, from the patients themselves. Detailed description of the oral preventive programme (OPP) compared to TAU (treatment as usual) can be found in the **Supplementary Table S7**.

### 2.3 Study measures

The age and sex of all patients, as well as the underlying disease, time of diagnosis, dialysis (in years), treatment measures, and medication intake were evaluated. Tongue swabs were collected from each patient to analyze changes in the oral microbiome during dental prophylaxis. Further clinical parameters (PBI, QHI, PSI, DMFT/dmft) were assessed and analyzed separately (see (23)). The dentition status was recorded in all patients.

#### 2.3.1 Prophylaxis interventions

For the OPP group (n=15), depending on the baseline degree of inflammation (gingival index, GI, papilla bleeding index, PBI and periodontal screening index, PSI), professional mechanical plaque removal, local chlorhexidine gel application, and mouth rinse were applied at need-related weekly appointments (with a limit of four appointments), focusing on local plaque control between t1 and t2. Between t2 and t3, the OPP group received re-motivation and examination of any clinical indices at t2 and t3, but no further intervention. The control group (TAU, n=15) received instructions according to the statutory health insurance program at time point t1 and a single intensive prophylaxis treatment at t2 (after swab sampling). No further interventions were done in the TAU group between t1 and t2 and between t2 and t3.

#### 2.3.2 16S rDNA Sequencing

DNA was isolated using a QIAMP DNA Mini Kit (Qiagen, Germany) according to the manufacturer’s instructions. DNA samples were processed with the Ion 16S Metagenomics Kit (Thermo Fisher Scientific, Germany) using two primer pools, thereby amplifying seven of the nine hypervariable bacterial 16S rDNA regions (pool 1: V2, V4, and V8; pool 2: V3, V6/7, and V9). Amplicons were pooled and cleaned using the NucleoMag NGS Clean-up (Macherey-Nagel, Germany), followed by library preparation using an Ion Plus Fragment Library Kit (Thermo Fisher Scientific, Germany). Metagenomic DNA libraries were sequenced on an Ion Torrent platform using S5 and S5 Prime devices (Thermo Fisher Scientific, Germany).

#### 2.3.3 Raw data analysis

Patient data were considered for final analysis until dropout (intent-to-treat principle). Primary data analysis was tailored to the generation of microbiome profiles from raw sequencing reads and performed in the Qiime2 environment. For read quality control, the nucleotides following sequences of three low quality (Phred score<20) base calls were eliminated, and only the read was retained in the analysis if at least 50% of the nucleotides remained after truncation. Thereafter, the residual sequences of library adapters (5’- ATCACCGACTGCCCATAGAGAGGCTGAGAC-3’) were eliminated, requiring a minimum remaining read length of 150 nucleotides. The reads were subsequently subjected to denoising and dereplication using dada2. The resulting representative amplicon sequences were then assigned to SILVA v138 taxonomies that were restricted to the domain of bacteria and where species names did not contain “uncultured” or “metagenome”.

#### 2.3.4 Bioinformatical analysis

Secondary data analysis involved bioinformatics and statistics to describe and visualize the microbiome dataset, which was performed in the R environment using diverse data science packages. For analysis at the phylum, genus, and species levels, postprocessing was limited to the exclusion of all reads that were assigned lower than the family level (assuming low-quality reads that did not reach the family level). To ensure saturation of the species-level microbiome profiles, we defined a minimum read threshold by rarefaction analysis and excluded all the samples that did not contain sufficient reads after postprocessing. This led to exclusion of one sample (p28) from analysis. Sequencing depth and genus richness for all analyzed samples is shown in **Supplementary Figure 2**.

Data visualization was performed with package ggplot2, radar plots were generated using R package ggradar. Alpha diversity was assessed using the Shannon diversity index ( $-\sum^{}p\times \log_{2}p$ ) at the genus and species levels. For rarefaction analysis, we first calculated a blueprint profile containing averaged species proportions across the study population; the rounded products of the target read depths using this blueprint were then utilized to calculate alpha diversities. Beta diversity (i.e., the distance between the microbiome profiles) was determined using the weighted UniFrac method (25). Differentially abundant taxa were determined using two-sided Welch two-sample t-tests on relative abundances; false discovery rates (FDR) were calculated using the Benjamini-Hochberg method to correct for multiple testing. FDR<0.05 were considered significant. Results are presented as mean ± standard deviation.

### 2.4 Data availability statement

Sequencing data and sample metadata are available at the Sequence Read Archive (https://www.ncbi.nlm.nih.gov/sra) under accession number PRJNA938485.

## 3 Results

Thirty patients with generalized gingivitis were included; 14 (46.7%) were female, 16 (53.3%) were male, and the mean age was 14.2 ± 5.2 years. There were no differences in clinical indices between the two groups; both groups had caries, with the Decayed, Missing, and Filled permanent/deciduous Teeth (DMFT/dmft) value being predominantly filled teeth. The measured biofilm values on the buccal and lingual sides showed relevant plaque amounts (Quigley-Hein Index) in both the OPP and TAU groups. Detailed patient characteristics for the OPP and TAU groups can be found in **Table 1**.

**Table 1:** Baseline demographic and clinical characteristics.

	Total (n=30*)	OPP (n=15*)	TAU (n=15)	p-value
Sex, n (%)				0.143^a^
Male	16 (53.3)	10 (66.7)	6 (40)	
Female	14 (46.7)	5 (33.3)	9 (60)	
Age (years), mean ± SD (range)	14.2 ± 5.2 (6-26)	13.5 ± 4.9 (6-21)	14.9 ± 5.5 (7-26)	0.539^b^
DMFT/dmft, mean ± SD	0.6 ± 1	0.6 ± 1	0.7 ± 1	
PBI, mean ± SD	1.1 ± 0.7	1 ± 0.6	1.1 ± 0.8	0.935^b^
QHI, mean ± SD	2.5 ± 0.9	2.6 ± 1	2.3 ± 0.9	0.233^b^
Dentition, n (%)				0.456
Mixed	13 (43.3)	7 (46.7)	6 (40)	
Permanent	17 (56.7)	8 (53.3)	9 (60)	
Primary disease, n (%)				0.085
*CAKUT*	11 (36.7)	8 (53.3)	3 (20)	
*Glomerulopathy*	9 (30)	2 (13.3)	7 (46.7)	
*Ciliopathy*	7 (23.3)	3 (20)	4 (26.7)	
*Systemic disease*	1 (3.3)	0 (0)	1 (6.7)	
*Renovascular*	0 (0)	0 (0)	0 (0)	
*Others*	2 (6.7)	2 (13.3)	0 (0)	
Therapy, n (%)				0.606
*Conservative*	7 (23.3)	2 (13.3)	5 (33.3)	
*Dialysis*	3 (10)	2 (13.3)	1 (6.7)	
*Post-transplant*	18 (60)	10 (66.7)	8 (53.3%)	
Duration of CKD disease (years)	11 (max 22)			
Dialysis duration (years)	2 patients 1 and 6			
Medication, n (%)				
*Immunosuppression*	22 (73.3)	11 (73.3)	11 (73.3)	
*-including cyclosporin*	5 (16.7)	3 (20)	2 (13.3)	
*Amlodipine*	17 (56.7)	8 (53.3)	9 (60)	
*Ramipril*	11 (36.7)	6 (40)	5 (33.4)	
*Amlodipine + ramipril*	4 (13.3)	2 (13.3)	2 (13.3)	

a Pearsons chi-squared test.

b Mann–Whitney U test.

CAKUT, congenital anomalies of the kidney and urinary tract; DMFT/dmft, decayed/missing/filled teeth-index, permanent (DMFT) or primary dentition (dmft); GI, gingival index; OPP, oral preventive program; PBI, papillary bleeding index; QHI, Quigley–Hein Plaque Index; TAU, treatment as usual. *One patient (p28) was not included in the analysis of the microbiome data, as sampling at t2 and t3 were missing. The clinical results were included according to the intent-to-treat principle.

### 3.1 Microbiome analysis

#### 3.1.1 Baseline characteristics

**Figure 1:**
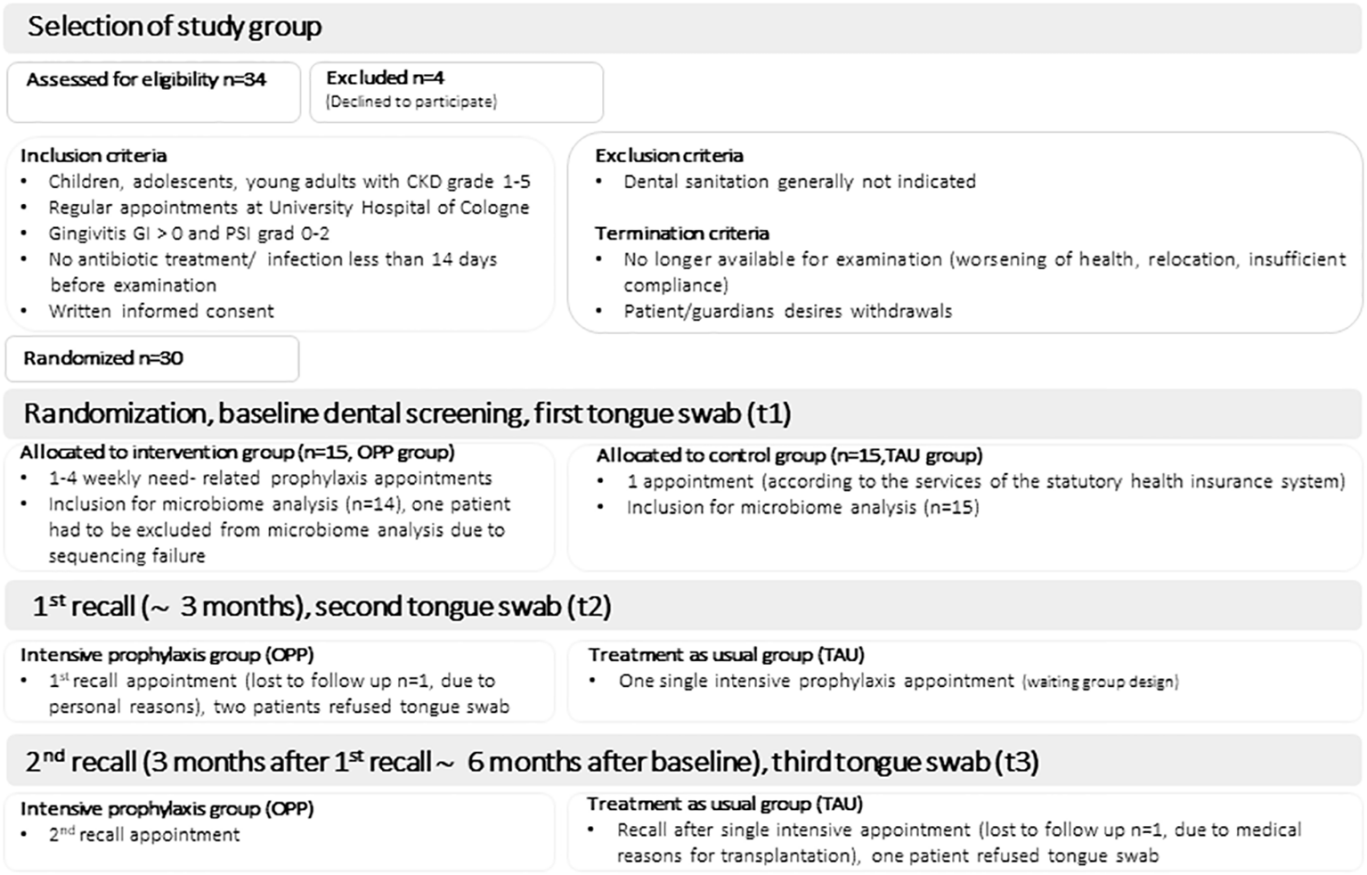
Consort diagram.

For the microbiome analysis, we included samples from 14 patients in the OPP group and 15 patients in the TAU group, see **Figure 1** (consort diagram). Detailed information on microbiome data availability for all patients and all study time points can be found in **Supplementary Table 1**. The microbiome profiles of the OPP and TAU groups were compared at baseline (t1) to describe group characteristics. The bacterial taxonomic composition in all samples (n=29) revealed 12 different phyla, 148 different genera, and 224 different species. No statistically significant differences between the two groups at baseline were found (**Supplementary Tables 2A–C**; **Supplementary Figures 2A–C**). We further compared the richness, alpha diversity, and beta diversity of the two groups at genus level at the beginning of the study (t1). There were no differences in the richness between the two groups at genus level (richness_OPP_ 47.2 ± 15.2 vs. richness_TAU_ 53.5 ± 11.8, p=0.268). Alpha diversity at genus level was higher in the TAU group, but the difference was statistically not significant (alpha diversity_OPP_ 3.0 ± 0.8 vs. alpha diversity_TAU_ 3.4 ± 0.55, p=0.052). Beta diversity visualization (Unifrac analysis) revealed a high similarity between both groups at baseline (see **Supplementary Figures 3A–C**).

### 3.2 Intervention analysis – impact of the intensive prophylaxis on the composition of the tongue microbiome

#### 3.2.1 Phylum level

**Figure 2:**
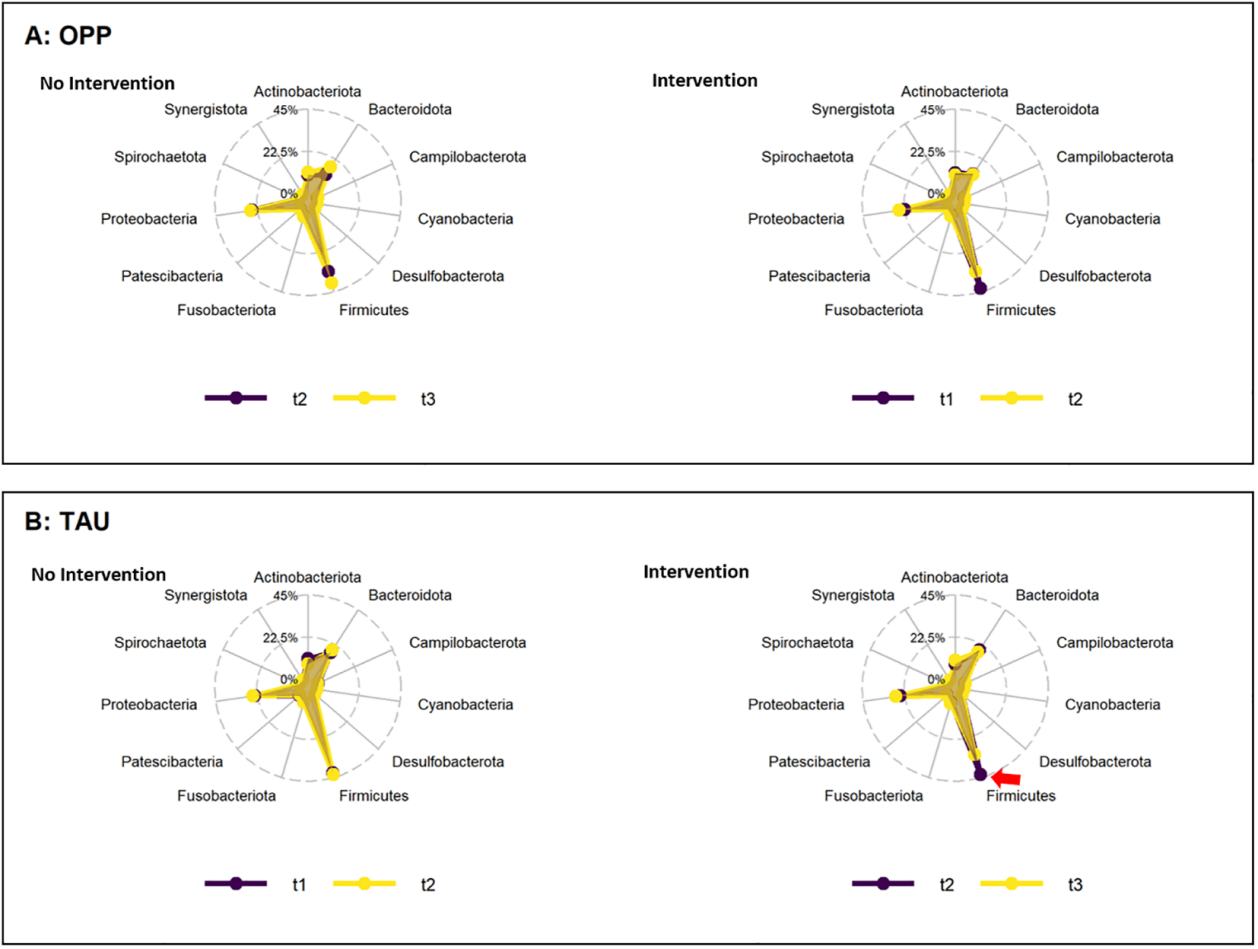
Change in abundance of phyla during the periods of intervention (right) and no intervention (left) in the OPP **(A)** and TAU **(B)** groups. Abbreviations: OPP, intensive oral prophylaxis program; TAU, treatment-as-usual. Red arrow=significant increase.

The main intervention (intensive prophylaxis) was performed in the OPP group between t1 and t2 (n_pairs_=11). At the phylum level, OPP led to a reduction in the average abundance of Firmicutes (44.3 ± 17.9 at t1 vs. 36.3 ± 12.1 at t2) and an increase in Proteobacteria (26.5 ± 18.7 at t1 vs. 31.5 ± 17.9 at t2). The same was seen when comparing baseline (t1) to the follow up at t3 in the OPP group (n_pairs_=13; Firmicutes: 46.7 ± 17.4 at t1 vs. 39.6 ± 13.5 at t3; Proteobacteria: 24.1 ± 18.2 at t1 vs. 27.2 ± 15.5 at t2). During the post-prophylaxis interval in the OPP group (t2 to t3, n_pairs_=12), no difference in the abundance of Firmicutes was observed (39.7 ± 16.3 at t2 vs. 38.9 ± 13.6 at t3; n_pairs_=11). Proteobacteria even slightly decreased from t2 to t3 (29.6 ± 18.3 at t2 vs. 26.4 ± 15.7 at t3). Because of extensive inter-individual variability and low sample count, none of these effects were significant (FDR 1) (**Supplementary Table 3**). In the TAU group (single prophylaxis after t2), treatment also led to a decrease in Firmicutes that reached significance when comparing baseline to post-intervention measurements (42.9 ± 7.1 at t1 to 34.8 ± 4.7 at t3, n_pairs_=14, p=0.003, FDR 0.02; 42.9 ± 13.9 at t2 to 34.8 ± 4.7 at t3, n_pairs_=14, p=0.04, FDR 0.32). The abundance of Proteobacteria displayed subtle fluctuations, none of which were significant (see **Supplementary Table 3**). In **Figure 2**, the average changes were visualized using a radar plot.

#### 3.2.2 Genus and species levels

**Figure 3:**
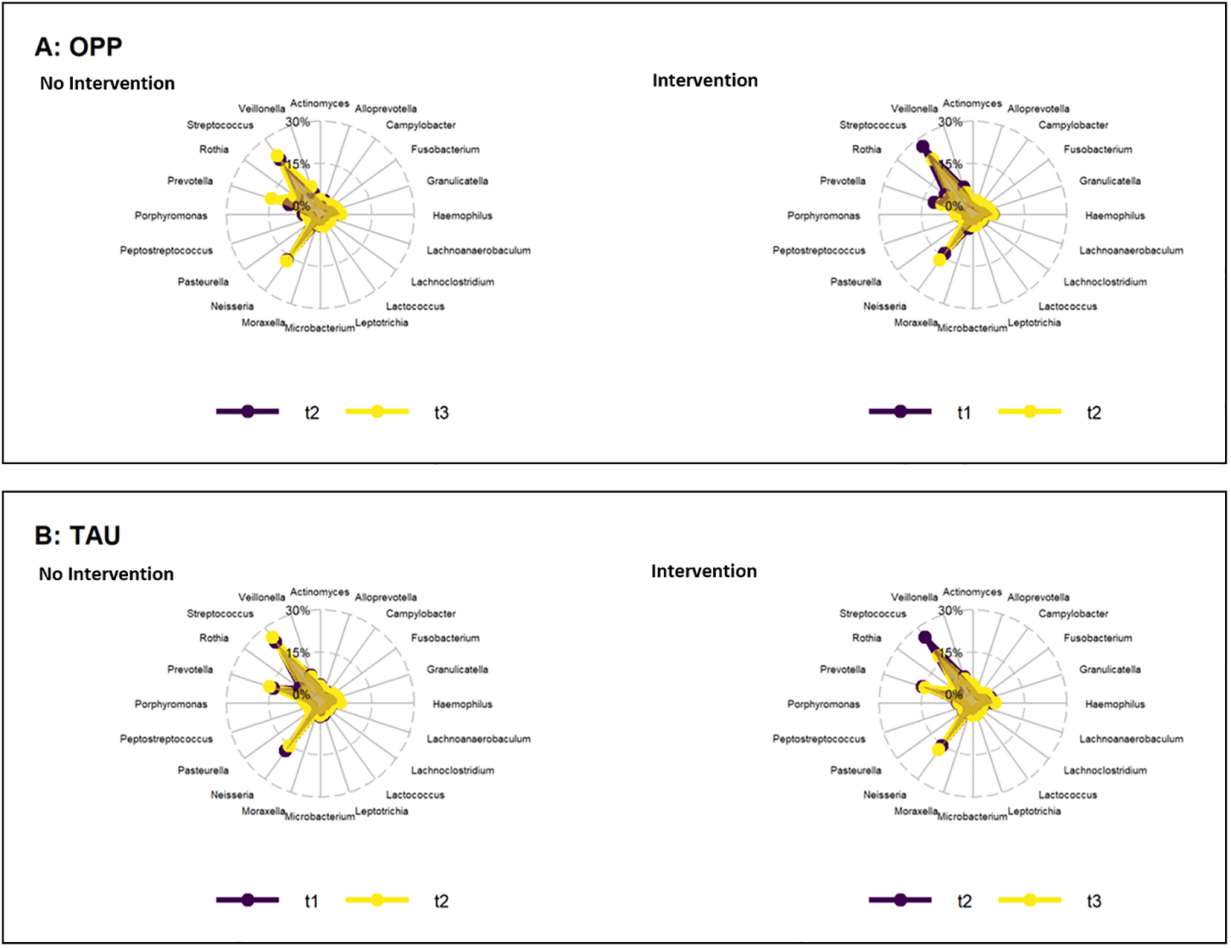
Change in abundance of genera during the periods of intervention (right) and no intervention (left) in the OPP **(A)** and TAU **(B)** groups. OPP, intensive oral prophylaxis program; TAU, treatment-as-usual.

The abundance of several genera and species increased or decreased within the time points and groups, with p-values<0.05 [see **Supplementary Tables 4** (genera) and **5** (species)]. However, when the FDR was determined to account for the rate of type I errors when testing null hypotheses in multiple comparisons, no significant differences were found (**Figure 3**).

##### 3.2.2.1 Effects of intervention on alpha diversity and richness at genus level

**Figure 4:**
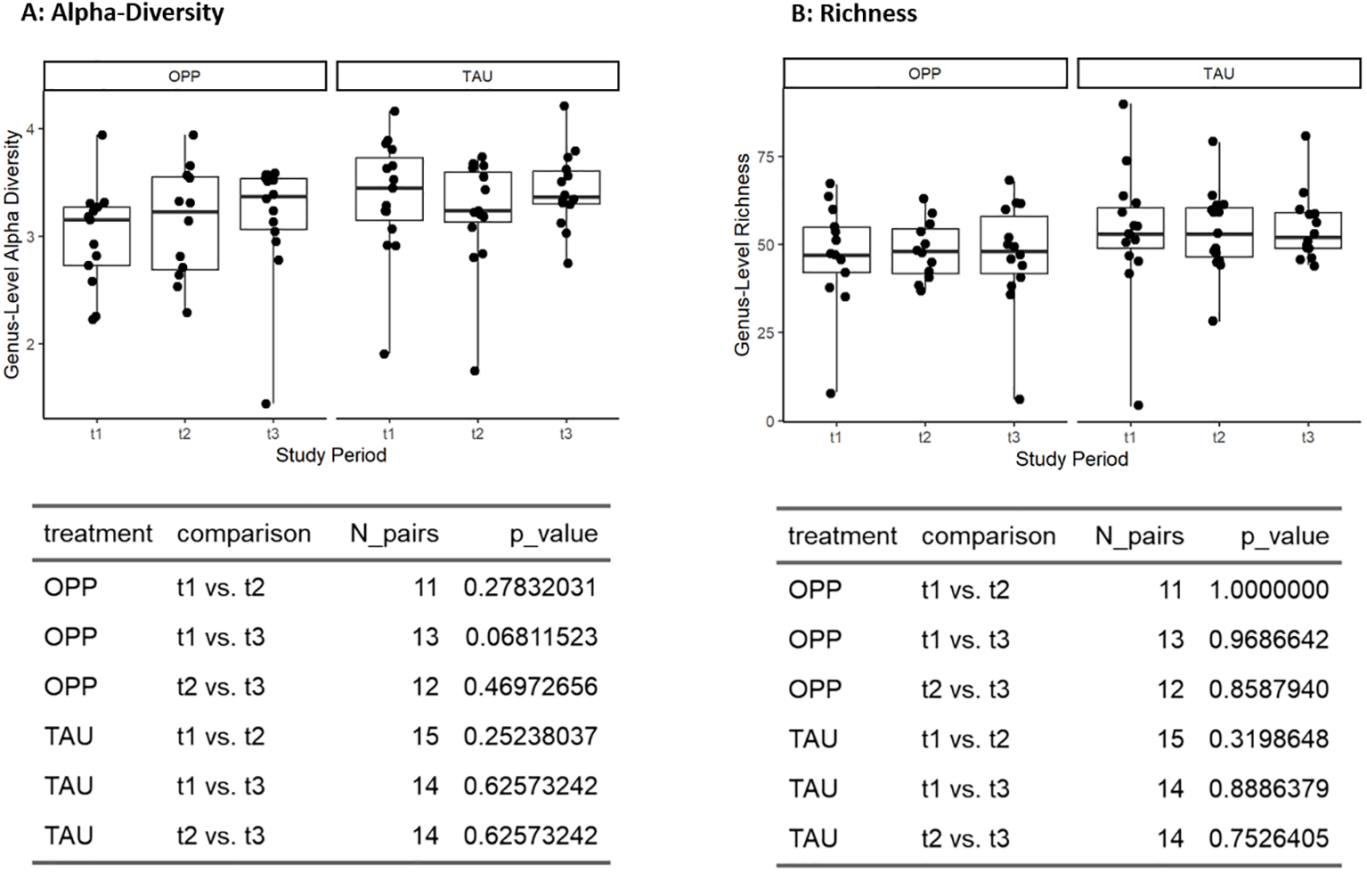
Effects of intervention on alpha diversity **(A)** and richness **(B)** at genus level. OPP, intensive oral prophylaxis program; TAU, treatment-as-usual.

The richness and alpha diversity (Shannon-Index) at genus level remained stable in the OPP and TAU groups, with no statistically significant effects with either intervention (**Figure 4**).

##### 3.2.2.2 Gingivitis and oral health cluster analysis

For further and more specific analysis, we performed comparative intervention analysis of a cluster of known gingivitis species and a cluster of bacteria known for oral health, according to a meta-analysis by Abusleme et al. (19). Of the 27 species associated with gingivitis, we found 14 species/highly similar species in our dataset. Of the 15 species associated with oral health, we found nine species/highly similar species in our dataset. All species used for the cluster analysis can be found in **Supplementary Table 6**.

**Figure 5:**
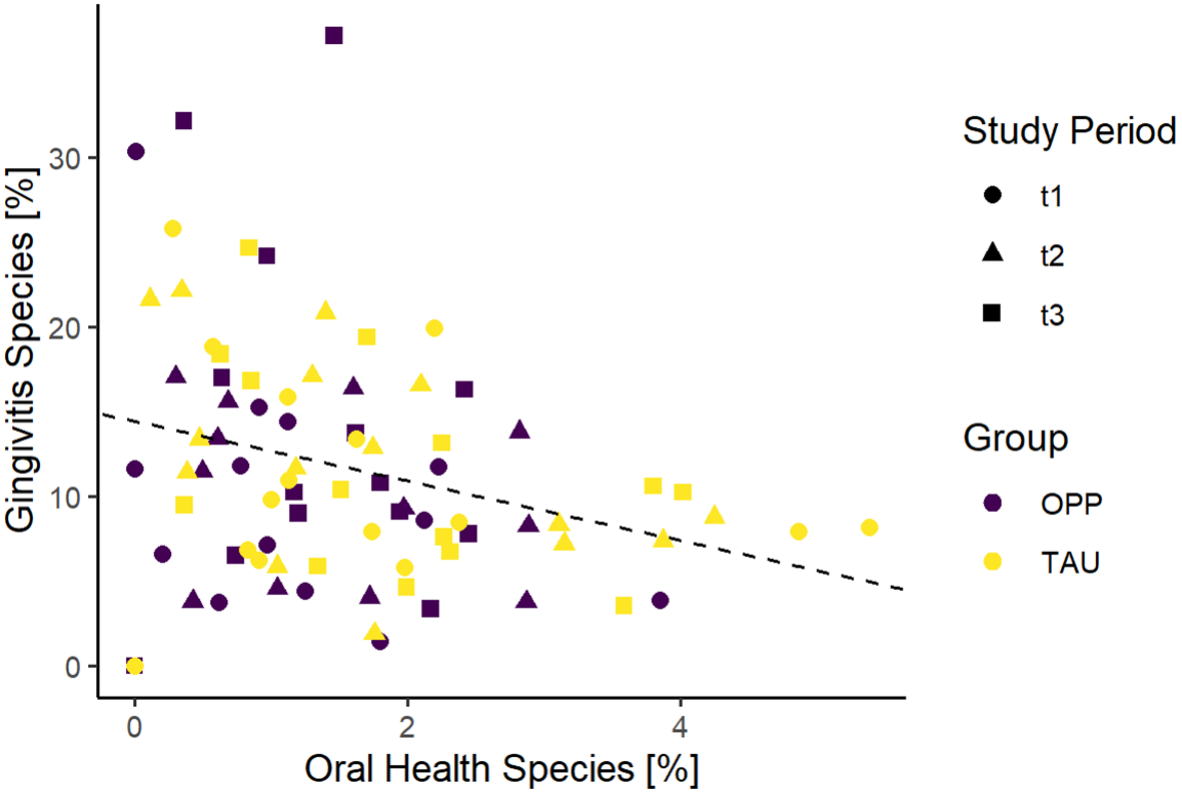
Comparison of gingivitis and oral health scores of all samples in the study. OPP, intensive oral prophylaxis program; TAU, treatment-as-usual.

As expected, comparison of the two clusters in our sample collective (all samples from all time points together) showed a negative correlation in both groups (**Figure 5**).

**Figure 6:**
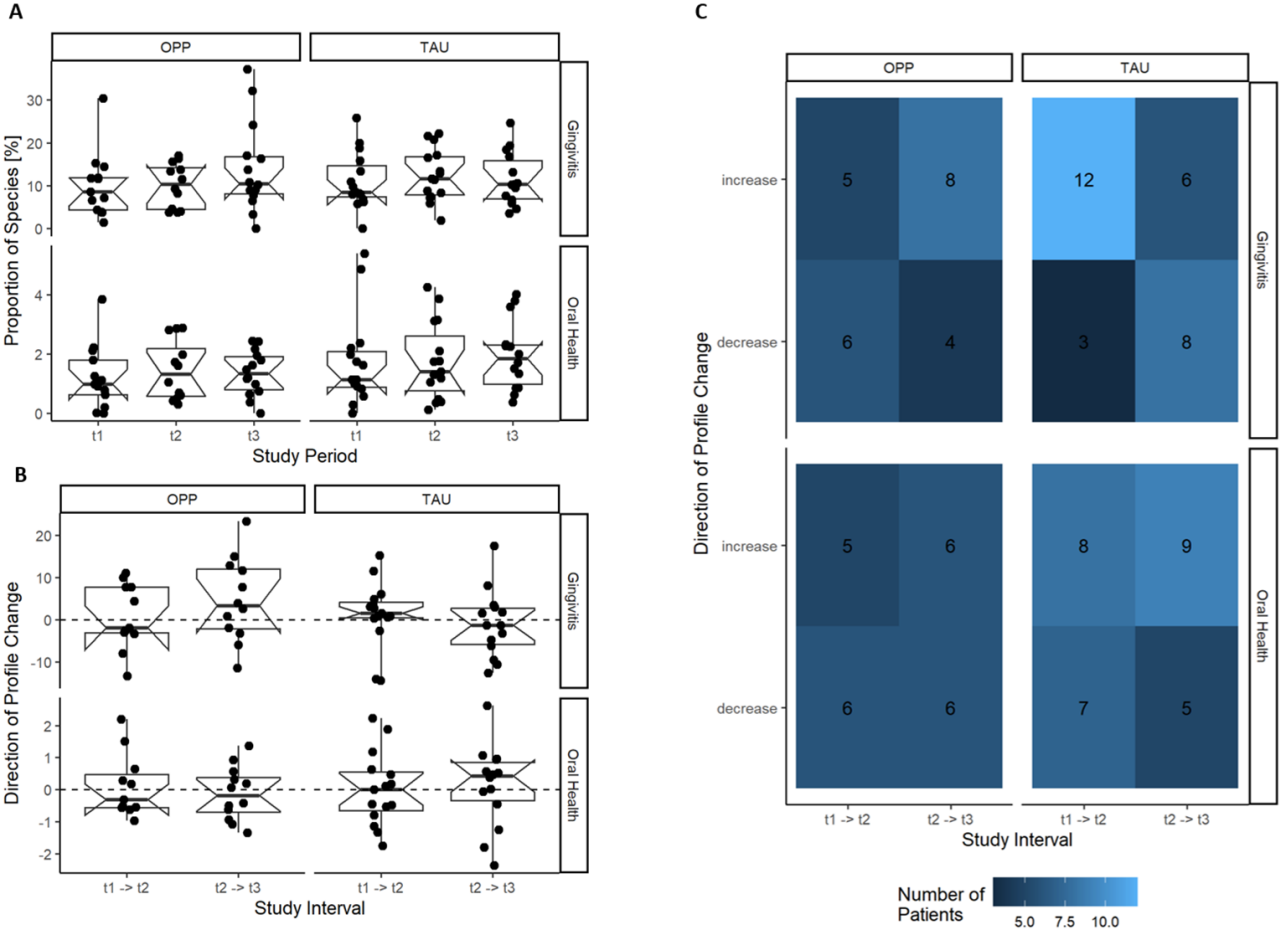
Development of the gingivitis and oral health cluster during OPP and TAU. No effect of the intervention on the development of the gingivitis or health cluster was found. **(A)** Gingivitis and oral health cluster per study time point. **(B)** Change in the gingivitis and health cluster per patient in the study intervals. **(C)** Frequency of the direction of change of the gingivitis and health cluster during the study. OPP, intensive oral prophylaxis program; TAU, treatment-as-usual.

We compared the relative abundance of the gingivitis and health cluster in the two groups at all time points, but there was no statistically significant effect of OPP or TAU on the development of gingivitis or health cluster (see **Figures 6A–C**).

## 4 Discussion

In recent decades, it has become evident that periodontal disease is not caused by a single pathogen. Instead, a critical role in the pathogenic outcome is played by members of the endogenous microbiota and their interactions with the host (26). In 2020, a new theory the “Inflammation-Mediated-Polymicrobial-Emergence and Dysbiotic-Exacerbation” (IMPEDE) model was published (27). In this model, inflammation is regarded as a hallmark of the dysbiotic events that drive the transition from oral health to periodontitis (27). Periodontitis is here defined as a multifactorial disease in which both the oral microbiota and the host immune response play a central role. Irrespective of the primary disease responsible for kidney failure, patients suffering from chronic kidney disease (CKD) exhibit a number of commonalities, including impairments of both the innate and adaptive immune systems (28). Patients diagnosed with CKD often exhibit generalized gingivitis (12). Additionally, it has been demonstrated that in adult patients with CKD, both the gut (9) and oral (15) microbiomes are altered compared to healthy controls. Nevertheless, the precise temporal and causal relationship between the development of dysbiosis in the oral microbiome, the underlying altered immune response in CKD patients and the subsequent clinical manifestations remains unclear. The accumulation of microbial plaque in the oral cavity of this vulnerable patient group gives rise to a complex host-mediated inflammatory immune response, resulting in the clinical picture of gingivitis and periodontitis (29). It would be beneficial to define any changes in the oral microbiome that induce the clinical picture and to ascertain whether the oral microbiome can be altered by prophylaxis to reduce clinical signs of gingivitis. As the tongue is considered to be the reservoir of periodontal disease-related bacteria (30), we aimed to investigate the influence of an intensive prophylaxis program on bacterial abundance in the tongue microbiome in young CKD patients. A group of young patients was subjected to a deliberate examination in order to ascertain whether the observations made in adult CKD patients regarding the dysbiosis in the oral microbiome could also be seen at an early age. This would potentially enable conclusions to be drawn about the temporal relationship between the duration of the disease and the oral microbiome.

While intensive prophylaxis has led to clear clinical improvements in the OPP group (23), analysis of the tongue microbiome in our study showed no statistically significant differences in the abundance of bacteria at a genus or species level compared to TAU in young CKD patients.

Similarly, Hall et al. recently showed that the tongue microbiome did not show any changes and was stable throughout their intervention, while experimentally induced gingivitis led to significant changes in the abundance of several genera and species in subgingival and supragingival plaque (31).

In earlier studies, preventive measures led to a reduction in plaque bacteria and the resulting amount in saliva in healthy patients (32). Since saliva constantly contaminates the tongue surface, our hypothesis was that clinical, successful preventive measures in our study group might have an influence on the tongue microbiome of the immunocompromised young patients. However—probably due to the conserved nature of the tongue microbiome—we did not see any change in our OPP group.

Another explanation could be of a statistical nature. Microbiome profiles display high inter-individual variability in terms of richness as well as relative abundance of taxa, thus impacting the power to detect differences in genus and species levels, especially if they are subtle. Likewise, the cumulation of genera to gingivitis and oral health clusters did not reveal significant differences, meaning that with the current datasets, the treatment effects were too subtle to be detected. In line with this, Yama et al. showed that prophylactic interventions with a focus on periodontal diseases and dental caries in adults have no effect on the oral tongue microbiome (33).

Even if not significant, we found the genus *Neisseria* to be increased after OPP and also after single prophylaxis in the TAU group (see **Supplementary Table 4**). *Neisseria* spp. are involved in nitrate reduction reactions in the oral cavity and are therefore considered to be helpful in the prevention of gingivitis (34). This is in line with the findings of Yama et al., where there was a significantly higher abundance of the genus *Neisseria* in the healthy group compared with the disease groups (33).

At phylum level, OPP resulted in a significant decrease in the phylum Firmicutes in our study. Guo et al. showed that the phylum Firmicutes was higher in CKD patients compared to healthy controls (15). However, since this phylum combines both gingivitis and oral health species, this result is of little clinical significance and does not help to understand the role of the tongue microbiome in gingivitis. Data regarding the role of the phylum Firmicutes in oral health studies are rare; neither Hall et al. nor Yama et al. reported any changes in Firmicutes due to their interventions (31, 33).

Our study has some limitations. First, CKD is extremely rare in childhood; thus, the sample size was low, and the study was underpowered. Given the high-dimensional and sparse nature of microbiome data, future gingivitis investigations should consider a higher number of samples. Furthermore, because no healthy control group was included, we cannot conclusively clarify whether the stability of the tongue microbiome only occurs in CKD children because of the most intensive preventive measures or whether it can also be generalized to healthy children. No fecal samples of the study participants were available to confirm the hypothesis whether gingivitis species are also detectable in the gut. This should also be tested in a further study. Our study only included traditional 16S rDNA microbiome analysis. Metatranscriptome and metabolome analysis provide more comprehensive and functional insights compared to traditional microbiome analysis and could provide a different insight to this research question. In particular, the biochemical activity and functional potential of the microbiome may prove to be more decisive than the composition in terms of its interaction with the immune system. Consequently, further studies are required to analyze the oral metatranscriptome (genes expressed by the entire microbial community) and metabolome (all metabolites associated with the microbiome). Furthermore, analysis of other oral cavity niches, such as saliva, could provide further insights and should be included in future studies.

In conclusion, our data confirm the highly conserved nature of the stable tongue microbiome in young CKD patients receiving prophylaxis of varying intensity. Despite the intensity of dental prophylaxis, combined with decreasing clinical signs of inflammation and plaque, no changes in the tongue microbiome were observed. Preventive dental care for young CKD patients therefore requires a needs-orientated approach, which must be adapted according to their clinical symptoms and aim to reduce the plaque amount instead of changing the composition of the tongue microbiome.

## Data Availability

Sequencing data and sample metadata are available at the Sequence Read Archive (https://www.ncbi.nlm.nih.gov/sra) under accession number PRJNA938485.
