# Supplementary material for: Intensive oral prophylaxis does not alter the tongue microbiome in young patients with chronic kidney disease: longitudinal, randomized, controlled study
Source: Front Immunol. 2024 Aug 19;15:1430655. doi: 10.3389/fimmu.2024.1430655 (PMC11366650; doi:10.3389/fimmu.2024.1430655)
Supplement: Supplementary file 1 [file DataSheet1.pdf]

Supplementary Material

This supplementary material is provided as supporting information alongside the article “Intensive oral prophylaxis does not alter tongue microbiome in young patients with CKD” on behalf of the authors, who remain responsible for the accuracy and appropriateness of the content. The same standards for ethics, copyright, attributions and permissions as for the article apply.

1 Supplementary and Tables

Supplementary table S1: Overwiev of samples taken from the patients at the different study time points

Patient number	Prophylaxcis group	Sampling for microbiome analysis		
		t1	t2	t3
1	OPP	+	+	+
2	OPP	+	+	+
3	TAU	+	+	+
4	drop out			
5	TAU	+	+	+
6	drop out			
7	TAU	+	+	+
8	OPP	+	+	+
9	OPP	+	+	+
10	OPP	+	-	+
11	TAU	+	+	+
12	TAU	+	+	+
13	OPP	+	+	+
14	TAU	+	+	-
15	drop out			
16	OPP	+	+	+
17	TAU	+	+	+
18	TAU	+	+	+
19	OPP	+	+	+
20	TAU	+	+	+
21	OPP	+	+	+
22	TAU	+	+	+
23	OPP	+	+	+
24	OPP	+	+	+
25	TAU	+	+	+
26	TAU	+	+	+
27	drop out			
28	OPP	+	+	
29	OPP	+	-	+
30	TAU	+	+	+
31	TAU	+	+	+
32	OPP	-	+	+
33	OPP	+	+	+
34	TAU	+	+	+

Abbreviations: IP= Intensive prophylaxis, TAU= Treatment as usual, t0=Baseline, t3=After 3 month, t6=After 6 month

Supplementary table 2a: Abundance at phylum level (baseline)

	Intensive oral prophylaxis program (OPP)		Treatment as usual (control group, TAU)		p-value	FDR
**Phylum**	**mean %**	**sd %**	**mean %**	**sd %**		
Firmicutes	46.66403846	17.3739883	42.73463333	6.931930649	0.4562501	0.5537277
Proteobacteria	24.13129231	18.17366846	23.84781333	12.660133	0.9628099	0.9628099
Bacteroidota	14.07895385	9.445816179	17.46755333	10.5487206	0.3781374	0.5537277
Actinobacteriota	11.74608462	11.87167488	10.8616	5.955501903	0.8105957	0.8842862
Fusobacteriota	2.299530769	1.790890987	2.895793333	1.922641483	0.4036348	0.5537277
Campilobacterota	0.6579	0.449161248	1.21074	0.979044372	0.0637026	0.3822157
Patescibacteria	0.419269231	0.587046921	0.96516	0.816685062	0.0509979	0.3822157
Spirochaetota	0.002223077	0.005511829	0.00414	0.007965264	0.4614397	0.5537277
Cyanobacteria	0.000738462	0.001436743	0.011513333	0.028041649	0.1593363	0.5537277
Desulfobacterota	0	0	6.66667E-05	0.000258199	0.3342819	0.5537277
Myxococcota	0	0	2.66667E-05	0.00010328	0.3342819	0.5537277
Synergistota	0	0	0.000933333	0.002680796	0.1989408	0.5537277
Bdellovibrionota	0.000000	0.000000	0.000010	0.000044	0.329257	0.521536
Verrucomicrobiota	0.000000	0.000000	0.000081	0.000371	0.329257	0.521536

Supplementary table 2b: Abundance at genus level (baseline)

Genus	Intensive oral prophylaxis program		Treatment as usual (control group)		p-value	FDR
	mean %	sd %	mean %	sd %		
Firmicutes->Bacilli->Lactobacillales->Streptococcaceae->Streptococcus	28.73543846	12.96495991	23.64317333	5.616792425	0.207742951	0.514260418
Proteobacteria->Gammaproteobacteria->Burkholderiales->Neisseriaceae->Neisseria	14.69667692	12.23704279	17.70122	10.60722523	0.497474291	0.639659555
Bacteroidota->Bacteroidia->Bacteroidales->Prevotellaceae->Prevotella	11.82985385	9.767572703	14.15344667	10.73581868	0.554046791	0.683324376
Actinobacteriota->Actinobacteria->Micrococcales->Micrococcaceae->Rothia	9.459838462	11.65171226	5.646793333	6.339701598	0.306503165	0.514260418
Firmicutes->Negativicutes->Veillonellales-Selenomonadales->Veillonellaceae->Veillonella	7.498053846	6.661065887	7.12994	2.759037063	0.854918455	0.897361215
Proteobacteria->Gammaproteobacteria->Pasteurellales->Pasteurellaceae->Haemophilus	4.130869231	3.345067737	2.729046667	2.05158622	0.204828537	0.514260418
Firmicutes->Bacilli->Lactobacillales->Carnobacteriaceae->Granulicatella	2.689046154	2.121911893	2.831413333	1.623424413	0.845578017	0.89389676
Proteobacteria->Gammaproteobacteria->Pseudomonadales->Moraxellaceae->Moraxella	1.942823077	4.980445941	0.334733333	1.01409315	0.273547793	0.514260418
Actinobacteriota->Actinobacteria->Actinomycetales->Actinomycetaceae->Actinomyces	1.541569231	1.780043823	3.0577	2.671998201	0.086288886	0.514260418
Fusobacteriota->Fusobacteriia->Fusobacteriales->Fusobacteriaceae->Fusobacterium	1.518023077	1.460095016	1.947933333	1.279477799	0.4187853	0.578854263
Firmicutes->Bacilli->Staphylococcales->Gemellaceae->Gemella	1.507923077	1.486382233	2.014613333	1.750971667	0.415125588	0.578854263
Bacteroidota->Bacteroidia->Bacteroidales->Porphyromonadaceae->Porphyromonas	1.116553846	1.368083218	1.446853333	2.01785908	0.612881956	0.719893091
Bacteroidota->Bacteroidia->Bacteroidales->Prevotellaceae->Alloprevotella	0.800523077	1.134899951	1.238026667	1.384062143	0.366717284	0.532099588
Fusobacteriota->Fusobacteriia->Fusobacteriales->Leptotrichiaceae->Leptotrichia	0.780976923	1.018048585	0.9467	1.440673355	0.725659943	0.813618724
Firmicutes->Clostridia->Lachnospirales->Lachnospiraceae->Lachnoanaerobaculum	0.678538462	1.120089405	0.453993333	0.608121624	0.527045477	0.66104009
Firmicutes->Clostridia->Lachnospirales->Lachnospiraceae->Lachnoclostridium	0.661053846	0.871836818	0.526506667	0.643949445	0.651123313	0.758366038
Campilobacterota->Campylobacteria->Campylobacterales->Campylobacteraceae->Campylobacter	0.657830769	0.449241543	1.210646667	0.978870819	0.063684391	0.514260418
Firmicutes->Clostridia->Peptostreptococcales-Tissierellales->Peptostreptococcaceae->Peptostreptococcus	0.624561538	1.682257639	0.362486667	0.52010092	0.597832697	0.713542251
Firmicutes->Bacilli->Lactobacillales->Enterococcaceae->Enterococcus	0.595338462	0.506478933	0.549306667	0.338303428	0.783591573	0.847095539

Proteobacteria->Gammaproteobacteria->Pasteurellales->Pasteurellaceae->Pasteurella	0.541176923	0.691710196	0.4493	0.511320123	0.697021512	0.793532183
Firmicutes->Clostridia->Lachnospirales->Lachnospiraceae->Oribacterium	0.475123077	1.088866159	0.373873333	0.362575227	0.753421274	0.832136929
Proteobacteria->Gammaproteobacteria->Enterobacterales->Yersiniaceae->Serratia	0.350215385	0.504428366	0.337566667	0.517559999	0.948380656	0.954832226
Actinobacteriota->Actinobacteria->Micrococcales->Microbacteriaceae->Microbacterium	0.344007692	0.769964715	1.37536	1.609285706	0.038707331	0.514260418
Firmicutes->Clostridia->Clostridia_UCG-014->Clostridia_UCG-014->Clostridia_UCG-014	0.337930769	0.491185695	0.351106667	0.531885458	0.946218674	0.954832226
Bacteroidota->Bacteroidia->Flavobacteriales->Flavobacteriaceae->Capnocytophaga	0.292361538	0.400818351	0.581053333	0.953153034	0.298200051	0.514260418
Firmicutes->Clostridia->Lachnospirales->Lachnospiraceae->Catonella	0.273884615	0.850103544	0.081393333	0.128735695	0.433992005	0.578854263
Firmicutes->Bacilli->Lactobacillales->Lactobacillaceae->Lactobacillus	0.244869231	0.237772445	0.426693333	0.308300564	0.090492317	0.514260418
Proteobacteria->Gammaproteobacteria->Pseudomonadales->Moraxellaceae->Acinetobacter	0.234423077	0.456364969	0.15888	0.254457996	0.602705608	0.71360344
Firmicutes->Bacilli->Erysipelotrichales->Erysipelotrichaceae->Solobacterium	0.223307692	0.408493012	0.332886667	0.311412569	0.438571636	0.579541091
Firmicutes->Negativicutes->Veillonellales-Selenomonadales->Veillonellaceae->Megasphaera	0.175023077	0.385372387	0.371093333	0.597964813	0.306752721	0.514260418
Patescibacteria->Saccharimonadia->Saccharimonadales->Saccharimonadaceae->TM7x	0.165607692	0.247046323	0.51234	0.596349626	0.053483493	0.514260418
Actinobacteriota->Actinobacteria->Micrococcales->Micrococcaceae->Micrococcus	0.147746154	0.532706333	0.004573333	0.017712444	0.351835218	0.515560517
Firmicutes->Clostridia->Lachnospirales->Lachnospiraceae->Stomatobaculum	0.126415385	0.228402083	0.118593333	0.161480766	0.918817743	0.937827765
Patescibacteria->Saccharimonadia->Saccharimonadales->Saccharimonadales->Saccharimonadales	0.124530769	0.243790294	0.246726667	0.332736027	0.274173102	0.514260418
Actinobacteriota->Coriobacteriia->Coriobacteriales->Atopobiaceae->Atopobium	0.097746154	0.131432274	0.34968	0.41258572	0.038767816	0.514260418
Proteobacteria->Gammaproteobacteria->Pasteurellales->Pasteurellaceae->Rodentibacter	0.088038462	0.221641074	0.059526667	0.139515537	0.693344792	0.793532183
Proteobacteria->Gammaproteobacteria->Burkholderiales->Burkholderiaceae->Lautropia	0.075646154	0.177507176	0.130473333	0.331691051	0.584486216	0.703284227
Proteobacteria->Gammaproteobacteria->Pasteurellales->Pasteurellaceae->Actinobacillus	0.073330769	0.158356199	0.066886667	0.093284946	0.898999604	0.930433156
Actinobacteriota->Actinobacteria->Corynebacteriales->Corynebacteriaceae->Corynebacterium	0.0539	0.09514961	0.02758	0.049505053	0.381600854	0.548319673
Firmicutes->Clostridia->Lachnospirales->Lachnospiraceae->Butyrivibrio	0.050976923	0.113977506	0.251453333	0.683360526	0.281108096	0.514260418
Proteobacteria->Gammaproteobacteria->Pasteurellales->Pasteurellaceae->Aggregatibacter	0.044530769	0.064967022	0.1118	0.142930313	0.116958528	0.514260418

Firmicutes->Negativicutes->Veillonellales-Selenomonadales->Selenomonadaceae->Selenomonas	0.044	0.082748202	0.208193333	0.247574541	0.026790182	0.514260418
Firmicutes->Bacilli->Lactobacillales->Carnobacteriaceae->Carnobacterium	0.039107692	0.060267023	0.028013333	0.03328867	0.562308624	0.685782024
Patescibacteria->Saccharimonadia->Saccharimonadales->Saccharimonadaceae->Saccharimonadaceae	0.037138462	0.070324007	0.054573333	0.064210863	0.502178765	0.639659555
Patescibacteria->Gracilibacteria->Absconditabacteriales_(SR1)->Absconditabacteriales_(SR1)->Absconditabacteriales_(SR1)	0.036138462	0.083864758	0.04058	0.079582421	0.887352306	0.924846065
Patescibacteria->Saccharimonadia->Saccharimonadales->Saccharimonadaceae->Candidatus_Saccharimonas	0.029638462	0.058728791	0.108193333	0.139334728	0.0609894	0.514260418
Firmicutes->Clostridia->Peptostreptococcales-Tissierellales->Anaerovoracaceae->[Eubacterium]_nodatum_group	0.027530769	0.051905963	0.152246667	0.177959104	0.019260609	0.514260418
Patescibacteria->Saccharimonadia->Saccharimonadales->Saccharimonadaceae->TM7a	0.025738462	0.075466312	0.000413333	0.000876573	0.249611887	0.514260418
Firmicutes->Clostridia->Peptostreptococcales-Tissierellales->Anaerovoracaceae->Mogibacterium	0.020530769	0.023393994	0.040706667	0.04485584	0.143014093	0.514260418
Bacteroidota->Bacteroidia->Bacteroidales->Bacteroidaceae->Bacteroides	0.020230769	0.043468022	0.007033333	0.009572182	0.30303562	0.514260418
Firmicutes->Clostridia->Peptostreptococcales-Tissierellales->Peptostreptococcaceae->[Eubacterium]_yurii_group	0.015238462	0.033544561	0.033506667	0.115101412	0.565306803	0.685782024
Firmicutes->Clostridia->Lachnospirales->Lachnospiraceae->Johnsonella	0.014069231	0.033660719	0.00784	0.012095088	0.536613795	0.667385224
Bacteroidota->Bacteroidia->Flavobacteriales->Flavobacteriaceae->Flavobacterium	0.013807692	0.031751521	0.00246	0.0053684	0.226041328	0.514260418
Proteobacteria->Gammaproteobacteria->Pasteurellales->Pasteurellaceae->Mannheimia	0.011207692	0.018094956	0.027726667	0.03565334	0.129834753	0.514260418
Proteobacteria->Gammaproteobacteria->Burkholderiales->Comamonadaceae->Brachymonas	0.010276923	0.032301371	0.002806667	0.007386036	0.429336287	0.578854263
Proteobacteria->Gammaproteobacteria->Alteromonadales->Shewanellaceae->Shewanella	0.009653846	0.012245381	0.00772	0.010115631	0.655884141	0.758366038
Proteobacteria->Gammaproteobacteria->Burkholderiales->Comamonadaceae->Acidovorax	0.005661538	0.014145997	0.002726667	0.006963729	0.50567681	0.639659555
Firmicutes->Bacilli->Lactobacillales->Carnobacteriaceae->Alkalibacterium	0.004853846	0.008620868	0.00212	0.00428289	0.313899518	0.514260418
Proteobacteria->Gammaproteobacteria->Burkholderiales->Hydrogenophilaceae->Thiobacillus	0.004476923	0.015008728	0.024086667	0.063154548	0.261218528	0.514260418
Firmicutes->Clostridia->Peptostreptococcales-Tissierellales->Peptostreptococcales-Tissierellales->Parvimonas	0.003323077	0.006145615	0.007666667	0.013264758	0.269416135	0.514260418
Bacteroidota->Bacteroidia->Bacteroidales->Tannerellaceae->Tannerella	0.003192308	0.006599553	0.022353333	0.04358843	0.113935137	0.514260418
Proteobacteria->Gammaproteobacteria->Burkholderiales->Neisseriaceae->Kingella	0.003192308	0.011510029	0.001826667	0.005742382	0.702826544	0.794033042
Proteobacteria->Gammaproteobacteria->Burkholderiales->Neisseriaceae->Simonsiella	0.002853846	0.005489325	0.00674	0.017838914	0.434140697	0.578854263

Proteobacteria->Gammaproteobacteria->Cardiobacteriales->Cardiobacteriaceae->Cardiobacterium	0.002746154	0.004325046	0.011446667	0.011384379	0.01323412	0.514260418
Firmicutes->Bacilli->Lactobacillales->Aerococcaceae->Abiotrophia	0.002569231	0.004573544	0.001913333	0.005512177	0.733588951	0.816324546
Firmicutes->Clostridia->Lachnospirales->Defluviitaleaceae->Defluviitaleaceae_UCG-011	0.002515385	0.009069348	0.003173333	0.006942464	0.833246899	0.891597066
Firmicutes->Clostridia->Lachnospirales->Lachnospiraceae->Lachnobacterium	0.002453846	0.008847468	0	0	0.337049058	0.514260418
Spirochaetota->Spirochaetia->Spirochaetales->Spirochaetaceae->Treponema	0.002223077	0.005511829	0.00414	0.007965264	0.461439719	0.604363525
Bacteroidota->Bacteroidia->Flavobacteriales->Weeksellaceae->Chryseobacterium	0.002176923	0.0055531	0.009466667	0.028506607	0.347496701	0.514295118
Firmicutes->Bacilli->Lactobacillales->Aerococcaceae->Globicatella	0.001684615	0.003649622	0.001306667	0.003547125	0.784135736	0.847095539
Firmicutes->Bacilli->RF39->RF39->RF39	0.001669231	0.006018497	0.001086667	0.004208642	0.772775462	0.847095539
Firmicutes->Bacilli->Lactobacillales->Streptococcaceae->Lactococcus	0.001638462	0.00502453	0.0122	0.047112252	0.402529142	0.567374409
Firmicutes->Negativicutes->Veillonellales-Selenomonadales->Veillonellaceae->Dialister	0.001492308	0.003462047	0.34954	1.202157525	0.281026193	0.514260418
Firmicutes->Clostridia->Lachnospirales->Lachnospiraceae->Moryella	0.000715385	0.002579356	0	0	0.337049058	0.514260418
Proteobacteria->Gammaproteobacteria->Vibrionales->Vibrionaceae->Vibrio	0.000569231	0.000934866	0.000113333	0.000438938	0.126111425	0.514260418
Proteobacteria->Gammaproteobacteria->Burkholderiales->Burkholderiaceae->Burkholderia-Caballeronia-Paraburkholderia	0.000569231	0.002052391	0	0	0.337049058	0.514260418
Cyanobacteria->Cyanobacteriia->Chloroplast->Chloroplast->Chloroplast	0.000523077	0.001305855	0.011513333	0.028041649	0.151653616	0.514260418
Proteobacteria->Gammaproteobacteria->Pseudomonadales->Pseudomonadaceae->Pseudomonas	0.000492308	0.001603362	0.00002	7.74597E-05	0.309483883	0.514260418
Proteobacteria->Gammaproteobacteria->Burkholderiales->Neisseriaceae->Snodgrassella	0.000476923	0.001719571	0	0	0.337049058	0.514260418
Patescibacteria->Gracilibacteria->Gracilibacteria->Gracilibacteria->Gracilibacteria	0.000407692	0.00100537	0.0001	0.000387298	0.315322888	0.514260418
Actinobacteriota->Actinobacteria->Micrococcales->Micrococcaceae->Paenarthrobacter	0.0004	0.001442221	0	0	0.337049058	0.514260418
Proteobacteria->Alphaproteobacteria->Sphingomonadales->Sphingomonadaceae->Sphingomonas	0.000346154	0.001189268	0.0003	0.001161895	0.918371974	0.937827765
Proteobacteria->Gammaproteobacteria->Burkholderiales->Comamonadaceae->Pelomonas	0.000307692	0.0011094	0	0	0.337049058	0.514260418
Proteobacteria->Gammaproteobacteria->Burkholderiales->Neisseriaceae->Alysiella	0.0003	0.001081665	0.00166	0.006429152	0.432788551	0.578854263
Proteobacteria->Gammaproteobacteria->Burkholderiales->Neisseriaceae->Eikenella	0.000253846	0.000915255	2.66667E-05	0.00010328	0.390562553	0.555800556
Cyanobacteria->Cyanobacteriia->Cyanobacteriales->Nostocaceae->Nostoc_PCC-73102	0.000215385	0.00077658	0	0	0.337049058	0.514260418

Proteobacteria->Gammaproteobacteria->Burkholderiales->Burkholderiaceae->Polynucleobacter	0.000192308	0.000693375	0	0	0.337049058	0.514260418
Proteobacteria->Gammaproteobacteria->Enterobacterales->Enterobacteriaceae->Enterobacter	0.000169231	0.00061017	0.00004	0.000154919	0.470269144	0.610524854
Firmicutes->Clostridia->Peptostreptococcales-Tissierellales->Anaerovoracaceae->[Eubacterium]_brachy_group	0.000153846	0.0005547	0.002893333	0.010151669	0.314349083	0.514260418
Bacteroidota->Bacteroidia->Bacteroidales->Paludibacteraceae->F0058	0.000138462	0.00049923	0.001233333	0.004050338	0.316339609	0.514260418
Proteobacteria->Gammaproteobacteria->Burkholderiales->Comamonadaceae->Comamonas	0.000138462	0.00049923	0	0	0.337049058	0.514260418
Proteobacteria->Gammaproteobacteria->Legionellales->Legionellaceae->Legionella	0.000138462	0.00049923	0	0	0.337049058	0.514260418
Actinobacteriota->Actinobacteria->Bifidobacteriales->Bifidobacteriaceae->Bifidobacterium	0.000138462	0.00049923	0.006473333	0.025071112	0.344473164	0.514295118
Actinobacteriota->Actinobacteria->Micrococcales->Microbacteriaceae->Candidatus_Aquiluna	0.000123077	0.000324432	0	0	0.196442844	0.514260418
Proteobacteria->Gammaproteobacteria->Enterobacterales->Enterobacteriaceae->Buttiauxella	0.000123077	0.00044376	0	0	0.337049058	0.514260418
Actinobacteriota->Acidimicrobiia->Microtrichales->Ilumatobacteraceae->Ilumatobacter	9.23077E-05	0.00033282	0	0	0.337049058	0.514260418
Bacteroidota->Bacteroidia->Flavobacteriales->Crocinitomicaceae->Fluviicola	9.23077E-05	0.00033282	0	0	0.337049058	0.514260418
Firmicutes->Clostridia->Clostridiales->Clostridiaceae->Clostridium	9.23077E-05	0.00033282	0	0	0.337049058	0.514260418
Proteobacteria->Gammaproteobacteria->Pseudomonadales->Moraxellaceae->Psychrobacter	7.69231E-05	0.00027735	0.00008	0.000309839	0.978094015	0.978094015
Patescibacteria->Gracilibacteria->JGI_0000069-P22->JGI_0000069-P22->JGI_0000069-P22	6.92308E-05	0.000249615	9.33333E-05	0.000361478	0.837378326	0.891597066
Firmicutes->Bacilli->Lactobacillales->Carnobacteriaceae->Trichococcus	6.15385E-05	0.00022188	0.032146667	0.124503505	0.335177823	0.514260418
Actinobacteriota->Actinobacteria->PeM15->PeM15->PeM15	6.15385E-05	0.00022188	0	0	0.337049058	0.514260418
Firmicutes->Clostridia->Peptostreptococcales-Tissierellales->Peptostreptococcales-Tissierellales->Anaerococcus	6.15385E-05	0.00022188	0	0	0.337049058	0.514260418
Actinobacteriota->Actinobacteria->Actinomycetales->Actinomycetaceae->Actinotignum	5.38462E-05	0.000194145	0.000613333	0.001493255	0.171833734	0.514260418
Proteobacteria->Gammaproteobacteria->Burkholderiales->Comamonadaceae->Limnohabitans	4.61538E-05	0.00016641	0	0	0.337049058	0.514260418
Firmicutes->Bacilli->Staphylococcales->Staphylococcaceae->Staphylococcus	3.84615E-05	0.000138675	0.034646667	0.132863834	0.33017543	0.514260418
Actinobacteriota->Actinobacteria->Frankiales->Sporichthyaceae->hgcI_clade	3.84615E-05	0.000138675	0	0	0.337049058	0.514260418
Actinobacteriota->Actinobacteria->Bifidobacteriales->Bifidobacteriaceae->Alloscardovia	3.84615E-05	0.000138675	0.00204	0.007900886	0.343244137	0.514295118
Firmicutes->Bacilli->Paenibacillales->Paenibacillaceae->Paenibacillus	3.07692E-05	0.00011094	0	0	0.337049058	0.514260418

Proteobacteria->Alphaproteobacteria->Acetobacterales->Acetobacteraceae->Rhodovastum	3.07692E-05	0.00011094	0	0	0.337049058	0.514260418
Bacteroidota->Bacteroidia->Flavobacteriales->Weeksellaceae->Bergeyella	2.30769E-05	8.3205E-05	0.001206667	0.004033514	0.274928477	0.514260418
Actinobacteriota->Actinobacteria->Actinomycetales->Actinomycetaceae->F0332	0	0	0.002446667	0.004959531	0.076748064	0.514260418
Firmicutes->Clostridia->Peptostreptococcales-Tissierellales->Peptostreptococcaceae->Filifactor	0	0	0.015846667	0.034852892	0.100064555	0.514260418
Firmicutes->Clostridia->Clostridia_vadinBB60_group->Clostridia_vadinBB60_group->Clostridia_vadinBB60_group	0	0	0.000866667	0.002097504	0.131854324	0.514260418
Fusobacteriota->Fusobacteriia->Fusobacteriales->Leptotrichiaceae->Streptobacillus	0	0	0.000826667	0.002184513	0.16484875	0.514260418
Firmicutes->Bacilli->Mycoplasmatales->Mycoplasmataceae->Mycoplasma	0	0	0.000673333	0.0018847	0.188124003	0.514260418
Actinobacteriota->Actinobacteria->Propionibacteriales->Propionibacteriaceae->Tessaracoccus	0	0	0.00012	0.000336367	0.188716492	0.514260418
Synergistota->Synergistia->Synergistales->Synergistaceae->Fretibacterium	0	0	0.000933333	0.002680796	0.198940847	0.514260418
Bacteroidota->Bacteroidia->Bacteroidales->Rikenellaceae->Rikenellaceae_RC9_gut_group	0	0	0.000986667	0.002871004	0.204455545	0.514260418
Actinobacteriota->Actinobacteria->Bifidobacteriales->Bifidobacteriaceae->Scardovia	0	0	0.000546667	0.001628701	0.21461403	0.514260418
Bacteroidota->Bacteroidia->Sphingobacteriales->Lentimicrobiaceae->Lentimicrobium	0	0	0.000166667	0.00061837	0.314236265	0.514260418
Firmicutes->Clostridia->Peptostreptococcales-Tissierellales->Anaerovoracaceae->Family_XIII_UCG-001	0	0	0.007673333	0.029498098	0.330801456	0.514260418
Actinobacteriota->Actinobacteria->Actinomycetales->Actinomycetaceae->Mobiluncus	0	0	0.00024	0.000929516	0.334281943	0.514260418
Proteobacteria->Alphaproteobacteria->Sphingomonadales->Sphingomonadaceae->Novosphingobium	0	0	0.00006	0.000232379	0.334281943	0.514260418
Actinobacteriota->Actinobacteria->Actinomycetales->Actinomycetaceae->Arcanobacterium	0	0	0.00664	0.025716609	0.334281943	0.514260418
Actinobacteriota->Coriobacteriia->Coriobacteriales->Atopobiaceae->Olsenella	0	0	0.00014	0.000542218	0.334281943	0.514260418
Actinobacteriota->Coriobacteriia->Coriobacteriales->Eggerthellaceae->Cryptobacterium	0	0	0.000153333	0.000593857	0.334281943	0.514260418
Actinobacteriota->Coriobacteriia->Coriobacteriales->Eggerthellaceae->Slackia	0	0	0.000453333	0.001755752	0.334281943	0.514260418
Desulfobacterota->Desulfovibrionia->Desulfovibrionales->Desulfovibrionaceae->Desulfovibrio	0	0	6.66667E-05	0.000258199	0.334281943	0.514260418
Firmicutes->Clostridia->Lachnospirales->Lachnospiraceae->Lachnospiraceae_NK4A136_group	0	0	0.001286667	0.004983239	0.334281943	0.514260418

Firmicutes->Clostridia->Lachnospirales->Lachnospiraceae->Shuttleworthia	0	0	0.000826667	0.003201666	0.334281943	0.514260418
Firmicutes->Clostridia->Peptostreptococcales-Tissierellales->Anaerovoracaceae->Amnipila	0	0	0.00428	0.016576369	0.334281943	0.514260418
Firmicutes->Clostridia->Peptostreptococcales-Tissierellales->Anaerovoracaceae->[Eubacterium]_saphenum_group	0	0	3.33333E-05	0.000129099	0.334281943	0.514260418
Firmicutes->Clostridia->Peptostreptococcales-Tissierellales->Peptostreptococcaceae->Peptoanaerobacter	0	0	0.000533333	0.002065591	0.334281943	0.514260418
Firmicutes->Negativicutes->Veillonellales-Selenomonadales->Veillonellaceae->Anaeroglobus	0	0	0.00028	0.001084435	0.334281943	0.514260418
Fusobacteriota->Fusobacteriia->Fusobacteriales->Leptotrichiaceae->Oceanivirga	0	0	0.000333333	0.001290994	0.334281943	0.514260418
Proteobacteria->Alphaproteobacteria->Rhodobacterales->Rhodobacteraceae->Paracoccus	0	0	3.33333E-05	0.000129099	0.334281943	0.514260418
Proteobacteria->Gammaproteobacteria->Acidiferrobacterales->Acidiferrobacteraceae->Sulfurifustis	0	0	0.00004	0.000154919	0.334281943	0.514260418
Proteobacteria->Gammaproteobacteria->Pseudomonadales->Moraxellaceae->Enhydrobacter	0	0	0.000253333	0.000981156	0.334281943	0.514260418
Actinobacteriota->Actinobacteria->Micrococcales->Microbacteriaceae->Agreia	0	0	6.66667E-06	2.58199E-05	0.334281943	0.514260418
Actinobacteriota->Actinobacteria->Propionibacteriales->Propionibacteriaceae->Pseudopropionibacterium	0	0	1.33333E-05	5.16398E-05	0.334281943	0.514260418
Bacteroidota->Bacteroidia->Bacteroidales->Bacteroidales_Incertae_Sedis->Phocaeicola	0	0	9.33333E-05	0.000361478	0.334281943	0.514260418
Myxococcota->Polyangia->Nannocystales->Nannocystaceae->Nannocystis	0	0	2.66667E-05	0.00010328	0.334281943	0.514260418
Proteobacteria->Alphaproteobacteria->Caulobacterales->Caulobacteraceae->Caulobacter	0	0	6.66667E-06	2.58199E-05	0.334281943	0.514260418
Firmicutes->Bacilli->Lactobacillales->P5D1-392->P5D1-392	0	0	0.000293333	0.001136075	0.334281943	0.514260418
Firmicutes->Clostridia->Lachnospirales->Lachnospiraceae->Howardella	0	0	0.000246667	0.000955336	0.334281943	0.514260418
Proteobacteria->Alphaproteobacteria->Rickettsiales->Mitochondria->Mitochondria	0	0	8.66667E-05	0.000335659	0.334281943	0.514260418
Proteobacteria->Gammaproteobacteria->Aeromonadales->Aeromonadaceae->Aeromonas	0	0	0.001133333	0.004389381	0.334281943	0.514260418

Supplementary Table 2c: Abundance at species level (baseline)

Species	Intensive oral prophylaxis program		Treatment as usual (control group)		p-value	FDR
	mean %	sd %	mean %	sd %		
Firmicutes->Bacilli->Lactobacillales->Streptococcaceae->Streptococcus->Streptococcus_salivarius	14.02418462	12.20380708	6.287313333	6.321928496	0.05477	0.5358
Bacteroidota->Bacteroidia->Bacteroidales->Prevotellaceae->Prevotella->Prevotella_melaninogenica	7.700492308	7.847739558	8.296646667	6.935079386	0.83425	0.9227
Actinobacteriota->Actinobacteria->Micrococcales->Micrococcaceae->Rothia->Rothia_mucilaginosa	7.255807692	10.54367139	3.918473333	6.074743827	0.32747	0.5358
Proteobacteria->Gammaproteobacteria->Burkholderiales->Neisseriaceae->Neisseria->Neisseria_meningitidis	7.127992308	6.543698789	7.611966667	4.472150196	0.82419	0.9227
Firmicutes->Negativicutes->Veillonellales-Selenomonadales->Veillonellaceae->Veillonella->Veillonella_parvula	3.616130769	4.124147546	3.34346	2.670391603	0.84029	0.9227
Proteobacteria->Gammaproteobacteria->Pasteurellales->Pasteurellaceae->Haemophilus->Haemophilus_parainfluenzae	1.456646154	1.52573329	0.917973333	0.728134336	0.2611	0.5358
Firmicutes->Negativicutes->Veillonellales-Selenomonadales->Veillonellaceae->Veillonella->Veillonella_atypica	1.2368	1.921178266	1.127606667	1.385874776	0.8665	0.927
Fusobacteriota->Fusobacteriia->Fusobacteriales->Fusobacteriaceae->Fusobacterium->Fusobacterium_periodonticum	1.142707692	1.159520307	1.270186667	0.86887486	0.74816	0.8639
Firmicutes->Bacilli->Lactobacillales->Streptococcaceae->Streptococcus->Streptococcus_parasanguinis	1.118853846	0.732565974	1.167426667	0.811364168	0.8691	0.927
Firmicutes->Bacilli->Staphylococcales->Gemellaceae->Gemella->Gemella_haemolysans	1.040623077	0.98210625	1.500213333	1.336948371	0.30579	0.5358
Bacteroidota->Bacteroidia->Bacteroidales->Porphyromonadaceae->Porphyromonas->Porphyromonas_pasteri	0.754892308	0.943780232	0.947886667	1.28283192	0.65145	0.7931
Proteobacteria->Gammaproteobacteria->Pasteurellales->Pasteurellaceae->Haemophilus->Haemophilus_pittmaniae	0.752523077	0.766662865	0.507786667	0.568048608	0.35376	0.544
Proteobacteria->Gammaproteobacteria->Pasteurellales->Pasteurellaceae->Pasteurella->Pasteurella_multocida	0.540684615	0.692082684	0.4493	0.511320123	0.69867	0.8328
Bacteroidota->Bacteroidia->Bacteroidales->Prevotellaceae->Prevotella->Prevotella_nanceiensis	0.463638462	0.576363186	0.38176	0.74771109	0.74657	0.8639
Actinobacteriota->Actinobacteria->Actinomycetales->Actinomycetaceae->Actinomyces->Actinomyces_graevenitzii	0.444638462	0.800346003	1.046773333	1.283316936	0.14427	0.5358
Bacteroidota->Bacteroidia->Bacteroidales->Prevotellaceae->Alloprevotella->Prevotellaceae_bacterium	0.411876923	0.725256783	0.672813333	0.967594907	0.42344	0.608
Fusobacteriota->Fusobacteriia->Fusobacteriales->Fusobacteriaceae->Fusobacterium->Fusobacterium_nucleatum	0.375315385	0.382590505	0.669333333	0.536204245	0.10425	0.5358
Firmicutes->Clostridia->Clostridia_UCG-014->Clostridia_UCG-014->Clostridia_UCG-014->Clostridiales_bacterium	0.337307692	0.489674484	0.349586667	0.53153077	0.94978	0.979
Bacteroidota->Bacteroidia->Bacteroidales->Prevotellaceae->Prevotella->Prevotella_salivae	0.329684615	0.604116752	0.78488	0.758359724	0.08916	0.5358

Firmicutes->Clostridia->Lachnospirales->Lachnospiraceae->Lachnoanaerobaculum->Lachnoanaerobaculum_umeaense	0.320561538	0.568339591	0.235893333	0.340797268	0.64439	0.7888
Firmicutes->Bacilli->Lactobacillales->Streptococcaceae->Streptococcus->Streptococcus_pneumoniae	0.253561538	0.290743823	0.70732	0.854600173	0.06967	0.5358
Bacteroidota->Bacteroidia->Bacteroidales->Prevotellaceae->Prevotella->Prevotella_histicola	0.238692308	0.46840294	0.239366667	0.423542381	0.99686	0.9988
Firmicutes->Bacilli->Lactobacillales->Streptococcaceae->Streptococcus->Streptococcus_suis	0.232407692	0.248474323	0.6711	1.556444765	0.29917	0.5358
Firmicutes->Bacilli->Erysipelotrichales->Erysipelotrichaceae->Solobacterium->Solobacterium_moorei	0.223307692	0.408493012	0.332886667	0.311412569	0.43857	0.614
Fusobacteriota->Fusobacteriia->Fusobacteriales->Leptotrichiaceae->Leptotrichia->Leptotrichia_buccalis	0.216715385	0.321731477	0.229726667	0.383765597	0.92302	0.9661
Fusobacteriota->Fusobacteriia->Fusobacteriales->Leptotrichiaceae->Leptotrichia->Leptotrichia_wadei	0.212415385	0.329700225	0.271733333	0.502676898	0.71193	0.8393
Bacteroidota->Bacteroidia->Bacteroidales->Prevotellaceae->Prevotella->Prevotella_pallens	0.191976923	0.26280964	0.322713333	0.261313312	0.19985	0.5358
Firmicutes->Negativicutes->Veillonellales-Selenomonadales->Veillonellaceae->Megasphaera->Megasphaera_micronuciformis	0.174238462	0.384568857	0.370513333	0.59652169	0.30516	0.5358
Bacteroidota->Bacteroidia->Bacteroidales->Prevotellaceae->Alloprevotella->Alloprevotella_rava	0.169661538	0.420304569	0.24802	0.634956778	0.70026	0.8328
Bacteroidota->Bacteroidia->Flavobacteriales->Flavobacteriaceae->Capnocytophaga->Capnocytophaga_sputigena	0.152815385	0.280676759	0.09206	0.284595585	0.5753	0.724
Actinobacteriota->Actinobacteria->Actinomycetales->Actinomycetaceae->Actinomyces->Schaalia_odontolytica	0.137369231	0.143788325	0.272273333	0.329242048	0.16648	0.5358
Patescibacteria->Saccharimonadia->Saccharimonadales->Saccharimonadales->Saccharimonadales->TM7_phylum	0.124530769	0.243790294	0.245873333	0.333043984	0.27773	0.5358
Bacteroidota->Bacteroidia->Bacteroidales->Prevotellaceae->Prevotella->Prevotella_loescheii	0.121161538	0.212860156	0.19686	0.343301874	0.48415	0.6544
Firmicutes->Bacilli->Lactobacillales->Streptococcaceae->Streptococcus->Streptococcus_sanguinis	0.104607692	0.199957785	0.063913333	0.113162518	0.52425	0.6768
Actinobacteriota->Actinobacteria->Micrococcales->Micrococcaceae->Rothia->Rothia_aeria	0.099815385	0.088594816	0.097486667	0.136234042	0.95716	0.979
Firmicutes->Negativicutes->Veillonellales-Selenomonadales->Veillonellaceae->Veillonella->Veillonella_rogosae	0.091023077	0.218714704	0.06226	0.166768067	0.70268	0.8328
Firmicutes->Clostridia->Lachnospirales->Lachnospiraceae->Stomatobaculum->Stomatobaculum_longum	0.087030769	0.140474911	0.096106667	0.11723891	0.85563	0.927
Patescibacteria->Saccharimonadia->Saccharimonadales->Saccharimonadaceae->TM7x->TM7_phylum	0.065523077	0.084875931	0.270113333	0.335865436	0.03685	0.5358
Patescibacteria->Saccharimonadia->Saccharimonadales->Saccharimonadaceae->TM7x->Candidatus_Saccharibacteria	0.065438462	0.152404377	0.1484	0.255357632	0.30034	0.5358
Bacteroidota->Bacteroidia->Bacteroidales->Prevotellaceae->Prevotella->Prevotella_intermedia	0.062707692	0.13190356	0.227233333	0.382041744	0.13554	0.5358
Bacteroidota->Bacteroidia->Bacteroidales->Prevotellaceae->Prevotella->Prevotella_veroralis	0.054276923	0.195698229	0.000566667	0.001195627	0.34193	0.5358
Firmicutes->Bacilli->Staphylococcales->Gemellaceae->Gemella->Gemella_sanguinis	0.053530769	0.086983115	0.060893333	0.058472319	0.79844	0.9033

Actinobacteriota->Actinobacteria->Actinomycetales->Actinomycetaceae->Actinomyces->Actinomyces_naeslundii	0.048976923	0.121866896	0.047326667	0.080319607	0.96721	0.9848
Proteobacteria->Gammaproteobacteria->Burkholderiales->Burkholderiaceae->Lautropia->Lautropia_mirabilis	0.0404	0.086956483	0.086173333	0.206407013	0.44339	0.6154
Bacteroidota->Bacteroidia->Bacteroidales->Prevotellaceae->Prevotella->Prevotella_shahii	0.039607692	0.085557578	0.068486667	0.147109696	0.52543	0.6768
Bacteroidota->Bacteroidia->Bacteroidales->Prevotellaceae->Prevotella->Prevotella_jejuni	0.034892308	0.102790892	0.2737	0.690339814	0.20604	0.5358
Bacteroidota->Bacteroidia->Flavobacteriales->Flavobacteriaceae->Capnocytophaga->Capnocytophaga_gingivalis	0.0344	0.062407331	0.06398	0.092172053	0.32456	0.5358
Proteobacteria->Gammaproteobacteria->Pasteurellales->Pasteurellaceae->Haemophilus->Haemophilus_haemolyticus	0.032492308	0.055201366	0.0572	0.079396186	0.34349	0.5358
Firmicutes->Clostridia->Lachnospirales->Lachnospiraceae->Catonella->Catonella_morbi	0.032415385	0.031001286	0.061033333	0.082275762	0.22743	0.5358
Firmicutes->Bacilli->Lactobacillales->Streptococcaceae->Streptococcus->Streptococcus_cristatus	0.031876923	0.071482844	0.021486667	0.03202782	0.63511	0.7847
Proteobacteria->Gammaproteobacteria->Pasteurellales->Pasteurellaceae->Aggregatibacter->Aggregatibacter_aphrophilus	0.030423077	0.048462806	0.071253333	0.094108971	0.15593	0.5358
Firmicutes->Bacilli->Lactobacillales->Streptococcaceae->Streptococcus->Streptococcus_gordonii	0.029638462	0.032099027	0.042806667	0.050607488	0.41316	0.6034
Patescibacteria->Saccharimonadia->Saccharimonadales->Saccharimonadaceae->Candidatus_Saccharimonas->TM7_phylum	0.029392308	0.05791775	0.107993333	0.138706754	0.05949	0.5358
Firmicutes->Bacilli->Lactobacillales->Streptococcaceae->Streptococcus->Streptococcus_infantis	0.027061538	0.035510293	0.0287	0.031399113	0.89886	0.9497
Bacteroidota->Bacteroidia->Flavobacteriales->Flavobacteriaceae->Capnocytophaga->Capnocytophaga_leadbetteri	0.026992308	0.036994514	0.253973333	0.574373245	0.14886	0.5358
Bacteroidota->Bacteroidia->Bacteroidales->Prevotellaceae->Prevotella->Prevotella_aurantiaca	0.0266	0.095907664	0.198606667	0.524045019	0.2313	0.5358
Proteobacteria->Gammaproteobacteria->Pasteurellales->Pasteurellaceae->Haemophilus->Haemophilus_influenzae	0.025807692	0.041820379	0.043106667	0.052054346	0.33887	0.5358
Actinobacteriota->Actinobacteria->Corynebacteriales->Corynebacteriaceae->Corynebacterium->Corynebacterium_durum	0.025607692	0.056218168	0.010906667	0.024764504	0.39593	0.5873
Bacteroidota->Bacteroidia->Flavobacteriales->Flavobacteriaceae->Capnocytophaga->Capnocytophaga_ochracea	0.025215385	0.043895593	0.122966667	0.328488403	0.27234	0.5358
Campilobacterota->Campylobacteria->Campylobacterales->Campylobacteraceae->Campylobacter->Campylobacter_concisus	0.024761538	0.026989613	0.032213333	0.030750235	0.50071	0.6716
Proteobacteria->Gammaproteobacteria->Pasteurellales->Pasteurellaceae->Haemophilus->Haemophilus_sputorum	0.023476923	0.044752396	0.023453333	0.03990143	0.99885	0.9988
Bacteroidota->Bacteroidia->Bacteroidales->Prevotellaceae->Prevotella->Prevotella_oulorum	0.022176923	0.050023131	0.037213333	0.070679012	0.51785	0.6768
Bacteroidota->Bacteroidia->Bacteroidales->Bacteroidaceae->Bacteroides->Bacteroidaceae_bacterium	0.020230769	0.043468022	0.007033333	0.009572182	0.30304	0.5358
Proteobacteria->Gammaproteobacteria->Burkholderiales->Neisseriaceae->Neisseria->Neisseria_elongata	0.018261538	0.021518424	0.016793333	0.024068131	0.86601	0.927
Firmicutes->Bacilli->Lactobacillales->Carnobacteriaceae->Granulicatella->Granulicatella_elegans	0.018015385	0.026746085	0.019866667	0.023256387	0.84782	0.9264

Bacteroidota->Bacteroidia->Bacteroidales->Porphyromonadaceae->Porphyromonas->Porphyromonas_endodontalis	0.016438462	0.044292937	0.015666667	0.027530234	0.95719	0.979
Firmicutes->Clostridia->Peptostreptococcales-Tissierellales->Peptostreptococcaceae->[Eubacterium]_yurii_group->[Eubacterium]_yurii	0.015238462	0.033544561	0.033506667	0.115101412	0.56531	0.7154
Bacteroidota->Bacteroidia->Bacteroidales->Prevotellaceae->Alloprevotella->Alloprevotella_tannerae	0.013792308	0.025032328	0.1659	0.392719589	0.1565	0.5358
Proteobacteria->Gammaproteobacteria->Pasteurellales->Pasteurellaceae->Actinobacillus->Haemophilus_parahaemolyticus	0.012892308	0.023722052	0.0138	0.032450072	0.93277	0.9673
Firmicutes->Bacilli->Staphylococcales->Gemellaceae->Gemella->Gemella_morbillorum	0.012730769	0.025967877	0.018386667	0.02118122	0.53779	0.6884
Firmicutes->Bacilli->Lactobacillales->Carnobacteriaceae->Granulicatella->Granulicatella_adiacens	0.012692308	0.017910679	0.008106667	0.019116129	0.51836	0.6768
Firmicutes->Clostridia->Lachnospirales->Lachnospiraceae->Johnsonella->Lachnospiraceae_bacterium	0.012615385	0.030480673	0.0056	0.011281274	0.44504	0.6154
Patescibacteria->Gracilibacteria->Absconditabacteriales_(SR1)->Absconditabacteriales_(SR1)->Absconditabacteriales_(SR1)->SR1_bacterium	0.011676923	0.025918756	0.008153333	0.017957961	0.68459	0.8244
Patescibacteria->Saccharimonadia->Saccharimonadales->Saccharimonadaceae->Saccharimonadaceae->TM7_bacterium	0.011315385	0.026034491	0.004686667	0.006256296	0.38636	0.5808
Proteobacteria->Gammaproteobacteria->Pasteurellales->Pasteurellaceae->Actinobacillus->Actinobacillus_pleuropneumoniae	0.010846154	0.032987134	0.019893333	0.039003452	0.51194	0.6768
Fusobacteriota->Fusobacteriia->Fusobacteriales->Leptotrichiaceae->Leptotrichia->Leptotrichia_shahii	0.010661538	0.022842962	0.028166667	0.071709898	0.3834	0.5803
Proteobacteria->Gammaproteobacteria->Pasteurellales->Pasteurellaceae->Aggregatibacter->Aggregatibacter_segnis	0.010376923	0.01409191	0.028266667	0.035922569	0.09175	0.5358
Proteobacteria->Gammaproteobacteria->Burkholderiales->Comamonadaceae->Brachymonas->Brachymonas_denitrificans	0.010276923	0.032301371	0.002806667	0.007386036	0.42934	0.6126
Bacteroidota->Bacteroidia->Bacteroidales->Porphyromonadaceae->Porphyromonas->Porphyromonas_catoniae	0.009984615	0.014507346	0.0258	0.0516609	0.27253	0.5358
Firmicutes->Bacilli->Lactobacillales->Streptococcaceae->Streptococcus->Streptococcus_oralis	0.009838462	0.016402568	0.00296	0.005230242	0.16906	0.5358
Actinobacteriota->Coriobacteriia->Coriobacteriales->Atopobiaceae->Atopobium->Lancefieldella_parvula	0.009246154	0.017183646	0.034193333	0.057675774	0.12923	0.5358
Firmicutes->Bacilli->Lactobacillales->Streptococcaceae->Streptococcus->Streptococcus_australis	0.009169231	0.019506767	0.003606667	0.006815368	0.3442	0.5358
Patescibacteria->Saccharimonadia->Saccharimonadales->Saccharimonadaceae->TM7a->candidate_division	0.0079	0.022781059	0.000133333	0.000516398	0.24262	0.5358
Bacteroidota->Bacteroidia->Bacteroidales->Prevotellaceae->Prevotella->Prevotella_nigrescens	0.0064	0.017533159	0.03286	0.051634288	0.079	0.5358
Firmicutes->Bacilli->Lactobacillales->Lactobacillaceae->Lactobacillus->Lactobacillus_plantarum	0.006384615	0.01628828	0.012666667	0.026524912	0.45145	0.6204
Firmicutes->Bacilli->Lactobacillales->Streptococcaceae->Streptococcus->Streptococcus_mitis	0.005361538	0.011051586	0.016393333	0.058490554	0.48496	0.6544
Firmicutes->Negativicutes->Veillonellales-Selenomonadales->Selenomonadaceae->Selenomonas->Selenomonas_sputigena	0.004507692	0.011704092	0.051666667	0.100043938	0.09092	0.5358

Proteobacteria->Gammaproteobacteria->Alteromonadales->Shewanellaceae->Shewanella->Shewanella_putrefaciens	0.004315385	0.007315947	0.003466667	0.006355725	0.74783	0.8639
Patescibacteria->Saccharimonadia->Saccharimonadales->Saccharimonadaceae->Saccharimonadaceae->TM7_phylum	0.004107692	0.014810495	0.00062	0.001751	0.41481	0.6034
Fusobacteriota->Fusobacteriia->Fusobacteriales->Leptotrichiaceae->Leptotrichia->Leptotrichia_hofstadii	0.004076923	0.009610684	0.005346667	0.009151102	0.72448	0.8496
Bacteroidota->Bacteroidia->Bacteroidales->Prevotellaceae->Prevotella->Prevotella_fusca	0.003653846	0.012817866	0.007453333	0.027513578	0.63753	0.7847
Firmicutes->Bacilli->Lactobacillales->Streptococcaceae->Streptococcus->Streptococcus_anginosus	0.003546154	0.009348673	0.040486667	0.135482898	0.30994	0.5358
Bacteroidota->Bacteroidia->Bacteroidales->Tannerellaceae->Tannerella->Tannerella_forsythia	0.003192308	0.006599553	0.022353333	0.04358843	0.11394	0.5358
Firmicutes->Negativicutes->Veillonellales-Selenomonadales->Selenomonadaceae->Selenomonas->Selenomonas_noxia	0.003115385	0.011232679	0.01658	0.028211122	0.10565	0.5358
Proteobacteria->Gammaproteobacteria->Burkholderiales->Neisseriaceae->Simonsiella->Simonsiella_muelleri	0.002853846	0.005489325	0.00674	0.017838914	0.43414	0.614
Bacteroidota->Bacteroidia->Flavobacteriales->Flavobacteriaceae->Capnocytophaga->Capnocytophaga_granulosa	0.002792308	0.006188762	0.002733333	0.006228353	0.98019	0.989
Actinobacteriota->Actinobacteria->Corynebacteriales->Corynebacteriaceae->Corynebacterium->Corynebacterium_diphtheriae	0.002546154	0.008138757	0.004313333	0.010450281	0.6197	0.7712
Firmicutes->Clostridia->Lachnospirales->Defluviitaleaceae->Defluviitaleaceae_UCG-011->Lachnospiraceae_bacterium	0.002515385	0.009069348	0.003173333	0.006942464	0.83325	0.9227
Firmicutes->Clostridia->Lachnospirales->Lachnospiraceae->Lachnobacterium->Lachnobacterium_bovis	0.002453846	0.008847468	0	0	0.33705	0.5358
Firmicutes->Bacilli->Lactobacillales->Lactobacillaceae->Lactobacillus->Lactobacillus_sakei	0.002284615	0.008237298	0.002586667	0.010018117	0.93093	0.9673
Spirochaetota->Spirochaetia->Spirochaetales->Spirochaetaceae->Treponema->Treponema_medium	0.002184615	0.005439033	0.002826667	0.006867258	0.7849	0.8925
Patescibacteria->Saccharimonadia->Saccharimonadales->Saccharimonadaceae->TM7a->TM7_phylum	0.002015385	0.007266573	0	0	0.33705	0.5358
Proteobacteria->Gammaproteobacteria->Pasteurellales->Pasteurellaceae->Rodentibacter->Pasteurellaceae_bacterium	0.001976923	0.00483083	0.004493333	0.011901709	0.46206	0.6311
Firmicutes->Bacilli->Lactobacillales->Streptococcaceae->Streptococcus->Streptococcus_peroris	0.001838462	0.006628667	0	0	0.33705	0.5358
Proteobacteria->Gammaproteobacteria->Burkholderiales->Neisseriaceae->Kingella->Kingella_oralis	0.001830769	0.006600932	0.001353333	0.004595163	0.82888	0.9227
Proteobacteria->Gammaproteobacteria->Cardiobacteriales->Cardiobacteriaceae->Cardiobacterium->Cardiobacterium_valvarum	0.001746154	0.00287304	0.007206667	0.008861995	0.03746	0.5358
Firmicutes->Bacilli->Lactobacillales->Aerococcaceae->Globicatella->Aerococcaceae_bacterium	0.001684615	0.003649622	0.001306667	0.003547125	0.78414	0.8925
Firmicutes->Bacilli->RF39->RF39->RF39->Firmicutes_oral	0.001669231	0.006018497	0.001086667	0.004208642	0.77278	0.8877
Firmicutes->Bacilli->Lactobacillales->Streptococcaceae->Lactococcus->Lactococcus_lactis	0.001638462	0.00502453	0.0122	0.047112252	0.40253	0.5932
Proteobacteria->Gammaproteobacteria->Pasteurellales->Pasteurellaceae->Haemophilus->[Haemophilus]_ducreyi	0.001638462	0.004008229	0.001953333	0.005488672	0.86261	0.927

Proteobacteria->Gammaproteobacteria->Burkholderiales->Neisseriaceae->Neisseria->Neisseria_perflava	0.001607692	0.003537528	0.00232	0.003825329	0.61324	0.7674
Proteobacteria->Gammaproteobacteria->Pasteurellales->Pasteurellaceae->Mannheimia->Mannheimia_haemolytica	0.001569231	0.003997371	0.01988	0.030412385	0.03608	0.5358
Firmicutes->Bacilli->Lactobacillales->Lactobacillaceae->Lactobacillus->Lactobacillus_curvatus	0.001523077	0.005491532	0	0	0.33705	0.5358
Proteobacteria->Gammaproteobacteria->Burkholderiales->Neisseriaceae->Neisseria->Neisseria_bacilliformis	0.001492308	0.004155812	0.001546667	0.004669333	0.97425	0.9875
Firmicutes->Clostridia->Peptostreptococcales-Tissierellales->Anaerovoracaceae->[Eubacterium]_nodatum_group->Eubacterium_sulci	0.001446154	0.005214182	0	0	0.33705	0.5358
Proteobacteria->Gammaproteobacteria->Enterobacterales->Yersiniaceae->Serratia->Hafnia_psychrotolerans	0.001192308	0.002962111	0.0006	0.001925765	0.5443	0.6927
Actinobacteriota->Actinobacteria->Actinomycetales->Actinomycetaceae->Actinomyces->Actinomyces_lingnae	0.001107692	0.003077461	0	0	0.21876	0.5358
Firmicutes->Clostridia->Peptostreptococcales-Tissierellales->Peptostreptococcales-Tissierellales->Parvimonas->Parvimonas_micra	0.001023077	0.001851645	0.001573333	0.002632887	0.52425	0.6768
Actinobacteriota->Actinobacteria->Corynebacteriales->Corynebacteriaceae->Corynebacterium->Corynebacterium_matruchotii	0.001007692	0.003010111	0.010513333	0.025131451	0.16759	0.5358
Proteobacteria->Gammaproteobacteria->Cardiobacteriales->Cardiobacteriaceae->Cardiobacterium->Cardiobacterium_hominis	0.001	0.00152698	0.00424	0.005893071	0.05682	0.5358
Firmicutes->Negativicutes->Veillonellales-Selenomonadales->Veillonellaceae->Dialister->Dialister_invisus	0.000984615	0.003063997	0.296933333	1.035572229	0.28702	0.5358
Proteobacteria->Gammaproteobacteria->Alteromonadales->Shewanellaceae->Shewanella->Shewanella_baltica	0.000946154	0.002276919	0.0004	0.00101207	0.43588	0.614
Actinobacteriota->Actinobacteria->Corynebacteriales->Corynebacteriaceae->Corynebacterium->Corynebacterium_argentoratense	0.000907692	0.003272731	0	0	0.33705	0.5358
Firmicutes->Bacilli->Lactobacillales->Lactobacillaceae->Lactobacillus->Lactobacillus_iners	0.000838462	0.003023116	0	0	0.33705	0.5358
Firmicutes->Clostridia->Lachnospirales->Lachnospiraceae->Moryella->Moryella_indoligenes	0.000715385	0.002579356	0	0	0.33705	0.5358
Fusobacteriota->Fusobacteriia->Fusobacteriales->Leptotrichiaceae->Leptotrichia->Leptotrichia_trevisanii	0.000623077	0.002246536	0.000206667	0.000548331	0.52575	0.6768
Firmicutes->Negativicutes->Veillonellales-Selenomonadales->Selenomonadaceae->Selenomonas->Selenomonas_massiliensis	0.000592308	0.002135596	0.008953333	0.020028225	0.12995	0.5358
Proteobacteria->Gammaproteobacteria->Burkholderiales->Burkholderiaceae->Burkholderia-Caballeronia-Paraburkholderia->Paraburkholderia_kururiensis	0.000569231	0.002052391	0	0	0.33705	0.5358
Bacteroidota->Bacteroidia->Bacteroidales->Prevotellaceae->Prevotella->unidentified_eubacterium	0.000553846	0.001996921	0	0	0.33705	0.5358
Bacteroidota->Bacteroidia->Flavobacteriales->Flavobacteriaceae->Flavobacterium->Flavobacterium_branchiophilum	0.000507692	0.00125596	0	0	0.17066	0.5358
Campilobacterota->Campylobacteria->Campylobacterales->Campylobacteraceae->Campylobacter->Campylobacter_showae	0.000492308	0.000838114	0.00138	0.003012403	0.28992	0.5358
Proteobacteria->Gammaproteobacteria->Burkholderiales->Neisseriaceae->Snodgrassella->Snodgrassella_alvi	0.000476923	0.001719571	0	0	0.33705	0.5358

Firmicutes->Negativicutes->Veillonellales-Selenomonadales->Veillonellaceae->Dialister->Dialister_pneumosintes	0.000461538	0.001664101	0.00312	0.009484966	0.30297	0.5358
Bacteroidota->Bacteroidia->Flavobacteriales->Weeksellaceae->Chryseobacterium->Chryseobacterium_reticulitermitis	0.000423077	0.001151921	0.00048	0.001302306	0.90327	0.9499
Patescibacteria->Gracilibacteria->Gracilibacteria->Gracilibacteria->Gracilibacteria->Gracilibacteria_bacterium	0.000407692	0.00100537	0.0001	0.000387298	0.31532	0.5358
Firmicutes->Negativicutes->Veillonellales-Selenomonadales->Selenomonadaceae->Selenomonas->Selenomonas_flueggei	0.000376923	0.001359015	0.002606667	0.006880248	0.23813	0.5358
Fusobacteriota->Fusobacteriia->Fusobacteriales->Leptotrichiaceae->Leptotrichia->Leptotrichia_hongkongensis	0.000261538	0.00041741	0.00066	0.001804676	0.41903	0.6056
Proteobacteria->Gammaproteobacteria->Burkholderiales->Neisseriaceae->Eikenella->Eikenella_corrodens	0.000253846	0.000915255	2.66667E-05	0.00010328	0.39056	0.5832
Patescibacteria->Saccharimonadia->Saccharimonadales->Saccharimonadaceae->Candidatus_Saccharimonas->Candidatus_Saccharimonas	0.000246154	0.00088752	0.0002	0.000774597	0.88551	0.9401
Proteobacteria->Gammaproteobacteria->Enterobacterales->Yersiniaceae->Serratia->Serratia_fonticola	0.000238462	0.000623884	0	0	0.19332	0.5358
Proteobacteria->Gammaproteobacteria->Burkholderiales->Neisseriaceae->Neisseria->Neisseria_oralis	0.000215385	0.00077658	0.009306667	0.01858073	0.07912	0.5358
Bacteroidota->Bacteroidia->Bacteroidales->Prevotellaceae->Prevotella->Massiliprevotella_massiliensis	0.000215385	0.000550524	0	0	0.18375	0.5358
Firmicutes->Bacilli->Lactobacillales->Carnobacteriaceae->Alkalibacterium->Alkalibacterium_olivapovliticus	0.000176923	0.000637905	0	0	0.33705	0.5358
Firmicutes->Negativicutes->Veillonellales-Selenomonadales->Selenomonadaceae->Selenomonas->Selenomonas_infelix	0.000153846	0.0005547	0.010126667	0.026920106	0.17339	0.5358
Firmicutes->Clostridia->Peptostreptococcales-Tissierellales->Anaerovoracaceae->[Eubacterium]_brachy_group->[Eubacterium]_brachy	0.000153846	0.0005547	0.002893333	0.010151669	0.31435	0.5358
Bacteroidota->Bacteroidia->Bacteroidales->Paludibacteraceae->F0058->Bacteroidetes_oral	0.000138462	0.00049923	0.001066667	0.003408742	0.31434	0.5358
Actinobacteriota->Actinobacteria->Bifidobacteriales->Bifidobacteriaceae->Bifidobacterium->Bifidobacterium_longum	0.000138462	0.00049923	0.006473333	0.025071112	0.34447	0.5358
Firmicutes->Negativicutes->Veillonellales-Selenomonadales->Veillonellaceae->Megasphaera->Megasphaera_elsdenii	0.000130769	0.000471495	0	0	0.33705	0.5358
Bacteroidota->Bacteroidia->Bacteroidales->Porphyromonadaceae->Porphyromonas->Porphyromonas_gingivalis	0.000130769	0.000471495	0.001493333	0.005783655	0.37853	0.5768
Actinobacteriota->Actinobacteria->Micrococcales->Microbacteriaceae->Candidatus_Aquiluna->Candidatus_Aquiluna	0.000123077	0.000324432	0	0	0.19644	0.5358
Actinobacteriota->Acidimicrobiia->Microtrichales->Ilumatobacteraceae->Ilumatobacter->Ilumatobacter_nonamiensis	9.23077E-05	0.00033282	0	0	0.33705	0.5358
Proteobacteria->Gammaproteobacteria->Pseudomonadales->Moraxellaceae->Acinetobacter->Eogystia_hippophaecolus	9.23077E-05	0.00033282	0	0	0.33705	0.5358
Firmicutes->Bacilli->Lactobacillales->Streptococcaceae->Streptococcus->Streptococcus_mutans	7.69231E-05	0.00027735	0.000126667	0.000359497	0.6833	0.8244

Bacteroidota->Bacteroidia->Bacteroidales->Prevotellaceae->Prevotella->Prevotella_denticola	6.92308E-05	0.000249615	0.003213333	0.010474791	0.26459	0.5358
Proteobacteria->Gammaproteobacteria->Pseudomonadales->Pseudomonadaceae->Pseudomonas->Pseudomonas_fluorescens	6.92308E-05	0.000249615	0	0	0.33705	0.5358
Patescibacteria->Gracilibacteria->JGI_0000069-P22->JGI_0000069-P22->JGI_0000069-P22->Gracilibacteria_bacterium	6.92308E-05	0.000249615	9.33333E-05	0.000361478	0.83738	0.9227
Bacteroidota->Bacteroidia->Bacteroidales->Prevotellaceae->Prevotella->Prevotella_buccae	6.15385E-05	0.00022188	0.001286667	0.004610061	0.32135	0.5358
Bacteroidota->Bacteroidia->Bacteroidales->Prevotellaceae->Prevotella->Prevotella_pleuritidis	4.61538E-05	0.00016641	0.010446667	0.029962664	0.20021	0.5358
Actinobacteriota->Actinobacteria->Frankiales->Sporichthyaceae->hgcI_clade->Candidatus_Planktophila	3.84615E-05	0.000138675	0	0	0.33705	0.5358
Actinobacteriota->Actinobacteria->Bifidobacteriales->Bifidobacteriaceae->Alloscardovia->Alloscardovia_omnicolens	3.84615E-05	0.000138675	0.00092	0.003563145	0.35457	0.544
Firmicutes->Bacilli->Paenibacillales->Paenibacillaceae->Paenibacillus->Paenibacillus_turicensis	3.07692E-05	0.00011094	0	0	0.33705	0.5358
Proteobacteria->Alphaproteobacteria->Acetobacterales->Acetobacteraceae->Rhodovastum->Acetobacteraceae_bacterium	3.07692E-05	0.00011094	0	0	0.33705	0.5358
Bacteroidota->Bacteroidia->Flavobacteriales->Weeksellaceae->Bergeyella->Flavobacteriaceae_bacterium	2.30769E-05	8.3205E-05	0.001206667	0.004033514	0.27493	0.5358
Proteobacteria->Gammaproteobacteria->Vibrionales->Vibrionaceae->Vibrio->Vibrio_litoralis	2.30769E-05	8.3205E-05	0	0	0.33705	0.5358
Spirochaetota->Spirochaetia->Spirochaetales->Spirochaetaceae->Treponema->Treponema_socranskii	2.30769E-05	8.3205E-05	0	0	0.33705	0.5358
Fusobacteriota->Fusobacteriia->Fusobacteriales->Leptotrichiaceae->Leptotrichia->Leptotrichia_goodfellowii	1.53846E-05	5.547E-05	0.001966667	0.006790505	0.2845	0.5358
Firmicutes->Bacilli->Lactobacillales->Lactobacillaceae->Lactobacillus->Lactobacillus_reuteri	1.53846E-05	5.547E-05	0.000866667	0.003356586	0.34271	0.5358
Actinobacteriota->Actinobacteria->Actinomycetales->Actinomycetaceae->Actinomyces->Actinomyces_oris	0	0	0.00592	0.012299373	0.0834	0.5358
Firmicutes->Clostridia->Peptostreptococcales-Tissierellales->Peptostreptococcaceae->Filifactor->Filifactor_alocis	0	0	0.015846667	0.034852892	0.10006	0.5358
Firmicutes->Clostridia->Clostridia_vadinBB60_group->Clostridia_vadinBB60_group->Clostridia_vadinBB60_group->Clostridiales_bacterium	0	0	0.000866667	0.002097504	0.13185	0.5358
Actinobacteriota->Actinobacteria->Bifidobacteriales->Bifidobacteriaceae->Scardovia->Scardovia_wiggsiae	0	0	0.00008	0.000214476	0.17057	0.5358
Firmicutes->Clostridia->Lachnospirales->Lachnospiraceae->Johnsonella->Firmicutes_oral	0	0	0.001306667	0.003530169	0.17365	0.5358
Actinobacteriota->Actinobacteria->Actinomycetales->Actinomycetaceae->Actinomyces->Actinomyces_dentalis	0	0	0.00004	0.000112122	0.18872	0.5358
Firmicutes->Clostridia->Lachnospirales->Lachnospiraceae->Catonella->Catonella_genomosp.	0	0	0.001526667	0.004426296	0.20292	0.5358
Synergistota->Synergistia->Synergistales->Synergistaceae->Fretibacterium->Synergistales_bacterium	0	0	0.000533333	0.001580084	0.21219	0.5358
Patescibacteria->Saccharimonadia->Saccharimonadales->Saccharimonadales->Saccharimonadales->Candidatus_Saccharibacteria	0	0	0.000853333	0.00275522	0.25023	0.5358

Bacteroidota->Bacteroidia->Bacteroidales->Prevotellaceae->Prevotella->Prevotella_baroniae	0	0	0.0002	0.000649175	0.25262	0.5358
Proteobacteria->Gammaproteobacteria->Pseudomonadales->Moraxellaceae->Acinetobacter->Acinetobacter_johnsonii	0	0	0.000526667	0.00171317	0.25359	0.5358
Proteobacteria->Gammaproteobacteria->Burkholderiales->Neisseriaceae->Kingella->Kingella_denitrificans	0	0	0.00032	0.001108538	0.28238	0.5358
Actinobacteriota->Actinobacteria->Bifidobacteriales->Bifidobacteriaceae->Scardovia->Scardovia_inopinata	0	0	0.000466667	0.001623782	0.28442	0.5358
Firmicutes->Clostridia->Lachnospirales->Lachnospiraceae->Johnsonella->Johnsonella_ignava	0	0	0.000226667	0.000824159	0.30482	0.5358
Spirochaetota->Spirochaetia->Spirochaetales->Spirochaetaceae->Treponema->Treponema_denticola	0	0	0.00054	0.001983431	0.30954	0.5358
Firmicutes->Bacilli->Staphylococcales->Staphylococcaceae->Staphylococcus->Staphylococcus_aureus	0	0	0.03226	0.123620744	0.32931	0.5358
Firmicutes->Clostridia->Peptostreptococcales-Tissierellales->Anaerovoracaceae->Family_XIII_UCG-001->Peptostreptococcaceae_bacterium	0	0	0.007673333	0.029498098	0.3308	0.5358
Actinobacteriota->Actinobacteria->Actinomycetales->Actinomycetaceae->Actinomyces->Actinomyces_viscosus	0	0	0.00054	0.002091411	0.33428	0.5358
Actinobacteriota->Actinobacteria->Actinomycetales->Actinomycetaceae->Mobiluncus->Mobiluncus_curtisii	0	0	0.00024	0.000929516	0.33428	0.5358
Firmicutes->Bacilli->Lactobacillales->Lactobacillaceae->Lactobacillus->Lactobacillus_crispatus	0	0	0.000473333	0.001833212	0.33428	0.5358
Actinobacteriota->Actinobacteria->Actinomycetales->Actinomycetaceae->Arcanobacterium->Arcanobacterium_phocae	0	0	0.00664	0.025716609	0.33428	0.5358
Actinobacteriota->Coriobacteriia->Coriobacteriales->Eggerthellaceae->Cryptobacterium->Cryptobacterium_curtum	0	0	0.000153333	0.000593857	0.33428	0.5358
Actinobacteriota->Coriobacteriia->Coriobacteriales->Eggerthellaceae->Slackia->Slackia_exigua	0	0	0.000453333	0.001755752	0.33428	0.5358
Bacteroidota->Bacteroidia->Bacteroidales->Prevotellaceae->Prevotella->Prevotella_dentalis	0	0	0.001773333	0.00686809	0.33428	0.5358
Bacteroidota->Bacteroidia->Bacteroidales->Prevotellaceae->Prevotella->Prevotella_enoeca	0	0	3.33333E-05	0.000129099	0.33428	0.5358
Bacteroidota->Bacteroidia->Bacteroidales->Prevotellaceae->Prevotella->Prevotella_multiformis	0	0	0.000353333	0.001368454	0.33428	0.5358
Bacteroidota->Bacteroidia->Bacteroidales->Rikenellaceae->Rikenellaceae_RC9_gut_group->Bacteroidales_oral	0	0	0.000613333	0.00237543	0.33428	0.5358
Cyanobacteria->Cyanobacteriia->Chloroplast->Chloroplast->Chloroplast->Arachis_hypogaea	0	0	0.00064	0.002478709	0.33428	0.5358
Desulfobacterota->Desulfovibrionia->Desulfovibrionales->Desulfovibrionaceae->Desulfovibrio->Desulfovibrio_fairfieldensis	0	0	6.66667E-05	0.000258199	0.33428	0.5358
Firmicutes->Bacilli->Lactobacillales->Lactobacillaceae->Lactobacillus->Lactobacillus_amylovorus	0	0	0.00104	0.004027903	0.33428	0.5358

Firmicutes->Bacilli->Lactobacillales->Lactobacillaceae->Lactobacillus->Lactobacillus_fermentum	0	0	0.00218	0.008443104	0.33428	0.5358
Firmicutes->Bacilli->Lactobacillales->Lactobacillaceae->Lactobacillus->Lactobacillus_gasseri	0	0	0.00004	0.000154919	0.33428	0.5358
Firmicutes->Bacilli->Lactobacillales->Lactobacillaceae->Lactobacillus->Lactobacillus_helveticus	0	0	0.003073333	0.011902969	0.33428	0.5358
Firmicutes->Bacilli->Lactobacillales->Streptococcaceae->Streptococcus->Coregonus_clupeaformis	0	0	0.000206667	0.000800417	0.33428	0.5358
Firmicutes->Clostridia->Lachnospirales->Lachnospiraceae->Butyrivibrio->Firmicutes_oral	0	0	3.33333E-05	0.000129099	0.33428	0.5358
Firmicutes->Clostridia->Lachnospirales->Lachnospiraceae->Shuttleworthia->Shuttleworthia_satelles	0	0	0.000826667	0.003201666	0.33428	0.5358
Firmicutes->Clostridia->Peptostreptococcales-Tissierellales->Anaerovoracaceae->Amnipila->Peptostreptococcaceae_bacterium	0	0	0.00428	0.016576369	0.33428	0.5358
Firmicutes->Clostridia->Peptostreptococcales-Tissierellales->Anaerovoracaceae->[Eubacterium]_saphenum_group->Eubacterium_saphenum	0	0	3.33333E-05	0.000129099	0.33428	0.5358
Firmicutes->Clostridia->Peptostreptococcales-Tissierellales->Peptostreptococcaceae->Peptoanaerobacter->Peptoanaerobacter_stomatis	0	0	0.000533333	0.002065591	0.33428	0.5358
Firmicutes->Negativicutes->Veillonellales-Selenomonadales->Veillonellaceae->Anaeroglobus->Anaeroglobus_geminatus	0	0	0.00028	0.001084435	0.33428	0.5358
Firmicutes->Negativicutes->Veillonellales-Selenomonadales->Veillonellaceae->Dialister->Dialister_micraerophilus	0	0	0.000386667	0.001497554	0.33428	0.5358
Fusobacteriota->Fusobacteriia->Fusobacteriales->Leptotrichiaceae->Oceanivirga->Oceanivirga_salmonicida	0	0	0.000333333	0.001290994	0.33428	0.5358
Proteobacteria->Gammaproteobacteria->Pseudomonadales->Moraxellaceae->Enhydrobacter->Moraxella_osloensis	0	0	0.000253333	0.000981156	0.33428	0.5358
Proteobacteria->Gammaproteobacteria->Pseudomonadales->Pseudomonadaceae->Pseudomonas->Pseudomonas_mendocina	0	0	0.00002	7.74597E-05	0.33428	0.5358
Spirochaetota->Spirochaetia->Spirochaetales->Spirochaetaceae->Treponema->Treponema_maltophilum	0	0	0.000126667	0.000490578	0.33428	0.5358
Actinobacteriota->Actinobacteria->Propionibacteriales->Propionibacteriaceae->Pseudopropionibacterium->Pseudopropionibacterium_propionicum	0	0	1.33333E-05	5.16398E-05	0.33428	0.5358
Bacteroidota->Bacteroidia->Bacteroidales->Bacteroidales_Incertae_Sedis->Phocaeicola->Phocaeicola_abscessus	0	0	9.33333E-05	0.000361478	0.33428	0.5358
Bacteroidota->Bacteroidia->Bacteroidales->Prevotellaceae->Prevotella->Prevotella_maculosa	0	0	0.000213333	0.000826236	0.33428	0.5358
Bacteroidota->Bacteroidia->Bacteroidales->Prevotellaceae->Prevotella->Prevotella_oralis	0	0	2.66667E-05	0.00010328	0.33428	0.5358
Bacteroidota->Bacteroidia->Bacteroidales->Prevotellaceae->Prevotella->Prevotella_saccharolytica	0	0	4.66667E-05	0.000180739	0.33428	0.5358
Campilobacterota->Campylobacteria->Campylobacterales->Campylobacteraceae->Campylobacter->Campylobacter_rectus	0	0	5.33333E-05	0.000206559	0.33428	0.5358
Firmicutes->Bacilli->Lactobacillales->Lactobacillaceae->Lactobacillus->Lactobacillus_acidophilus	0	0	6.66667E-06	2.58199E-05	0.33428	0.5358
Spirochaetota->Spirochaetia->Spirochaetales->Spirochaetaceae->Treponema->Treponema_refringens	0	0	2.66667E-05	0.00010328	0.33428	0.5358

Bacteroidota->Bacteroidia->Bacteroidales->Porphyromonadaceae->Porphyromonas->Porphyromonas_asaccharolytica	0	0	7.33333E-05	0.000284019	0.33428	0.5358
Firmicutes->Bacilli->Lactobacillales->Lactobacillaceae->Lactobacillus->Lactobacillus_vaginalis	0	0	0.000173333	0.000671317	0.33428	0.5358
Firmicutes->Bacilli->Lactobacillales->P5D1-392->P5D1-392->bacterium_enrichment	0	0	0.000293333	0.001136075	0.33428	0.5358
Firmicutes->Clostridia->Lachnospirales->Lachnospiraceae->Howardella->Howardella_ureilytica	0	0	0.000246667	0.000955336	0.33428	0.5358

Supplementary Table S3: Change in abundance of phyla during the periods of intervention in the OPP and TAU groups

treatment group	comparison	group1	group2	N_pairs	phylum	group1_mean	group1_sd	group2_mean	group2_sd	p_value	FDR
OPP	t0 vs. t3	t0	t3	11	Actinobacteriota	12.474	12.740	11.373	13.256	0.832	1
OPP	t0 vs. t3	t0	t3	11	Bacteroidota	13.288	9.662	15.784	6.784	0.358	1
OPP	t0 vs. t3	t0	t3	11	Campilobacterota	0.660	0.399	1.117	0.944	0.164	1
OPP	t0 vs. t3	t0	t3	11	Cyanobacteria	0.001	0.002	0.001	0.003	0.982	1
OPP	t0 vs. t3	t0	t3	11	Firmicutes	44.301	17.953	36.324	12.056	0.132	1
OPP	t0 vs. t3	t0	t3	11	Fusobacteriota	2.444	1.802	3.333	2.707	0.239	1
OPP	t0 vs. t3	t0	t3	11	Patescibacteria	0.336	0.457	0.544	0.570	0.285	1
OPP	t0 vs. t3	t0	t3	11	Proteobacteria	26.493	18.694	31.522	17.948	0.466	1
OPP	t0 vs. t3	t0	t3	11	Spirochaetota	0.003	0.006	0.003	0.006	0.780	1
OPP	t0 vs. t6	t0	t6	13	Actinobacteriota	11.746	11.872	10.526	6.356	0.675	1
OPP	t0 vs. t6	t0	t6	13	Bacteroidota	14.079	9.446	17.709	10.001	0.253	1
OPP	t0 vs. t6	t0	t6	13	Campilobacterota	0.658	0.449	1.003	0.988	0.322	1
OPP	t0 vs. t6	t0	t6	13	Cyanobacteria	0.001	0.001	0.018	0.061	0.335	1
OPP	t0 vs. t6	t0	t6	13	Firmicutes	46.664	17.374	39.653	13.508	0.255	1
OPP	t0 vs. t6	t0	t6	13	Fusobacteriota	2.300	1.791	3.251	2.863	0.313	1
OPP	t0 vs. t6	t0	t6	13	Patescibacteria	0.419	0.587	0.671	1.031	0.462	1
OPP	t0 vs. t6	t0	t6	13	Proteobacteria	24.131	18.174	27.168	15.459	0.609	1
OPP	t0 vs. t6	t0	t6	13	Spirochaetota	0.002	0.006	0.000	0.001	0.284	1
OPP	t3 vs. t6	t3	t6	12	Actinobacteriota	11.148	12.663	11.199	6.801	0.984	1
OPP	t3 vs. t6	t3	t6	12	Bacteroidota	14.902	7.153	18.659	10.030	0.148	1
OPP	t3 vs. t6	t3	t6	12	Campilobacterota	1.050	0.929	1.179	1.370	0.798	1
OPP	t3 vs. t6	t3	t6	12	Cyanobacteria	0.001	0.003	0.028	0.069	0.186	1
OPP	t3 vs. t6	t3	t6	12	Firmicutes	39.660	16.301	38.920	13.619	0.889	1
OPP	t3 vs. t6	t3	t6	12	Fusobacteriota	3.114	2.689	3.126	2.963	0.991	1
OPP	t3 vs. t6	t3	t6	12	Patescibacteria	0.500	0.564	0.475	0.926	0.936	1
OPP	t3 vs. t6	t3	t6	12	Proteobacteria	29.620	18.336	26.413	15.698	0.442	1
OPP	t3 vs. t6	t3	t6	12	Spirochaetota	0.003	0.005	0.000	0.001	0.128	1
OPP	t3 vs. t6	t3	t6	12	Synergistota	0.000	0.001	0.000	0.000	0.427	1
TAU	t0 vs. t3	t0	t3	15	Actinobacteriota	10.862	5.956	7.935	4.523	0.102	1
TAU	t0 vs. t3	t0	t3	15	Bacteroidota	17.468	10.549	19.127	11.985	0.614	1
TAU	t0 vs. t3	t0	t3	15	Campilobacterota	1.211	0.979	0.794	0.854	0.148	1
TAU	t0 vs. t3	t0	t3	15	Cyanobacteria	0.012	0.028	0.002	0.004	0.149	1
TAU	t0 vs. t3	t0	t3	15	Desulfobacterota	0.000	0.000	0.001	0.002	0.263	1
TAU	t0 vs. t3	t0	t3	15	Firmicutes	42.735	6.932	43.634	13.704	0.822	1
TAU	t0 vs. t3	t0	t3	15	Fusobacteriota	2.896	1.923	3.055	2.008	0.781	1

TAU	t0 vs. t3	t0	t3	15	Patescibacteria	0.965	0.817	0.597	0.602	0.036	0
TAU	t0 vs. t3	t0	t3	15	Proteobacteria	23.848	12.660	24.844	13.592	0.436	1
TAU	t0 vs. t3	t0	t3	15	Spirochaetota	0.004	0.008	0.006	0.013	0.351	1
TAU	t0 vs. t3	t0	t3	15	Synergistota	0.001	0.003	0.006	0.022	0.345	1
TAU	t0 vs. t6	t0	t6	14	Actinobacteriota	11.146	6.074	10.762	5.241	0.788	1
TAU	t0 vs. t6	t0	t6	14	Bacteroidota	15.809	8.683	19.114	11.918	0.257	1
TAU	t0 vs. t6	t0	t6	14	Campilobacterota	1.214	1.016	1.279	0.894	0.645	1
TAU	t0 vs. t6	t0	t6	14	Cyanobacteria	0.012	0.029	0.034	0.086	0.396	1
TAU	t0 vs. t6	t0	t6	14	Firmicutes	42.973	7.130	34.772	4.712	**0.003**	**0**
TAU	t0 vs. t6	t0	t6	14	Fusobacteriota	2.948	1.984	3.748	2.555	0.265	1
TAU	t0 vs. t6	t0	t6	14	Patescibacteria	0.934	0.838	1.038	1.701	0.787	1
TAU	t0 vs. t6	t0	t6	14	Proteobacteria	24.959	12.355	29.242	12.816	0.093	1
TAU	t0 vs. t6	t0	t6	14	Spirochaetota	0.004	0.008	0.010	0.022	0.272	1
TAU	t3 vs. t6	t3	t6	14	Actinobacteriota	8.102	4.645	10.762	5.241	0.055	0
TAU	t3 vs. t6	t3	t6	14	Bacteroidota	18.601	12.256	19.114	11.918	0.865	1
TAU	t3 vs. t6	t3	t6	14	Campilobacterota	0.782	0.884	1.279	0.894	0.118	1
TAU	t3 vs. t6	t3	t6	14	Cyanobacteria	0.002	0.004	0.034	0.086	0.179	1
TAU	t3 vs. t6	t3	t6	14	Firmicutes	42.928	13.936	34.772	4.712	**0.035**	**0**
TAU	t3 vs. t6	t3	t6	14	Fusobacteriota	3.106	2.074	3.748	2.555	0.544	1
TAU	t3 vs. t6	t3	t6	14	Patescibacteria	0.492	0.458	1.038	1.701	0.228	1
TAU	t3 vs. t6	t3	t6	14	Proteobacteria	25.975	13.353	29.242	12.816	0.255	1
TAU	t3 vs. t6	t3	t6	14	Spirochaetota	0.006	0.013	0.010	0.022	0.563	1

Abbreviations: OPP, Intensive oral prophylaxis program; TAU, Treatmen-As-Usual

Supplementary Table S4: Change in abundance of genera during the periods of intervention in the OPP and TAU groups

treatment group	comparison	group1	group2	N_pairs	genus	group1_mean	group1_sd	group2_mean	group2_sd	p_value	FDR
OPP	t0 vs. t3	t0	t3	11	Actinomyces	1.61945	1.87378	2.07643	2.84186	0.60786	1
OPP	t0 vs. t3	t0	t3	11	Alloscardovia	0.00005	0.00015	0.02522	0.05705	0.17324	1
OPP	t0 vs. t3	t0	t3	11	Corynebacterium	0.04618	0.09264	0.00336	0.00600	0.15892	1
OPP	t0 vs. t3	t0	t3	11	Microbacterium	0.38885	0.83375	0.82607	1.58733	0.42180	1
OPP	t0 vs. t3	t0	t3	11	Paenarthrobacter	0.00047	0.00157	0.00148	0.00360	0.43560	1
OPP	t0 vs. t3	t0	t3	11	Rothia	10.04575	12.43579	8.05446	13.37028	0.69319	1
OPP	t0 vs. t3	t0	t3	11	Atopobium	0.09262	0.13179	0.13765	0.26631	0.63788	1
OPP	t0 vs. t3	t0	t3	11	Bacteroides	0.02150	0.04712	0.00050	0.00129	0.17211	1
OPP	t0 vs. t3	t0	t3	11	F0058	0.00016	0.00054	0.00093	0.00279	0.28670	1
OPP	t0 vs. t3	t0	t3	11	Porphyromonas	1.28988	1.42332	3.12155	3.12899	0.07637	1
OPP	t0 vs. t3	t0	t3	11	Alloprevotella	0.84335	1.21188	2.32688	3.19027	0.09730	1
OPP	t0 vs. t3	t0	t3	11	Prevotella	10.76903	9.95753	9.98419	6.85094	0.70888	1
OPP	t0 vs. t3	t0	t3	11	Tannerella	0.00377	0.00706	0.00457	0.01278	0.75071	1
OPP	t0 vs. t3	t0	t3	11	Capnocytophaga	0.34474	0.41614	0.34233	0.25427	0.97467	1
OPP	t0 vs. t3	t0	t3	11	Flavobacterium	0.01335	0.03398	0.00144	0.00348	0.27521	1
OPP	t0 vs. t3	t0	t3	11	Bergeyella	0.00003	0.00009	0.00009	0.00030	0.52864	1
OPP	t0 vs. t3	t0	t3	11	Chryseobacterium	0.00257	0.00599	0.00151	0.00267	0.50943	1
OPP	t0 vs. t3	t0	t3	11	Campylobacter	0.66016	0.39867	1.11651	0.94395	0.16395	1
OPP	t0 vs. t3	t0	t3	11	Chloroplast	0.00062	0.00141	0.00085	0.00283	0.81934	1
OPP	t0 vs. t3	t0	t3	11	Solobacterium	0.12546	0.12581	0.11005	0.10322	0.55144	1
OPP	t0 vs. t3	t0	t3	11	Abiotrophia	0.00174	0.00315	0.00101	0.00335	0.63971	1
OPP	t0 vs. t3	t0	t3	11	Globicatella	0.00116	0.00315	0.00313	0.00819	0.24675	1
OPP	t0 vs. t3	t0	t3	11	Carnobacterium	0.04360	0.06460	0.01965	0.03250	0.18843	1
OPP	t0 vs. t3	t0	t3	11	Granulicatella	3.04234	2.09745	3.26943	1.99440	0.65902	1
OPP	t0 vs. t3	t0	t3	11	Enterococcus	0.67545	0.50710	0.42921	0.27871	0.05705	1
OPP	t0 vs. t3	t0	t3	11	Lactobacillus	0.26916	0.24728	0.19513	0.15006	0.33869	1
OPP	t0 vs. t3	t0	t3	11	Lactococcus	0.00194	0.00545	0.00149	0.00494	0.85311	1
OPP	t0 vs. t3	t0	t3	11	Streptococcus	27.48310	13.76152	21.37661	10.78689	0.20281	1
OPP	t0 vs. t3	t0	t3	11	RF39	0.00197	0.00654	0.00630	0.02089	0.53809	1
OPP	t0 vs. t3	t0	t3	11	Gemella	1.69636	1.53390	1.85113	1.26643	0.67951	1
OPP	t0 vs. t3	t0	t3	11	Staphylococcus	0.00005	0.00015	0.00019	0.00063	0.48505	1
OPP	t0 vs. t3	t0	t3	11	Clostridia_UCG-014	0.24979	0.31401	0.41954	0.76118	0.43254	1
OPP	t0 vs. t3	t0	t3	11	Butyrivibrio	0.04662	0.11971	0.07034	0.21015	0.41490	1
OPP	t0 vs. t3	t0	t3	11	Catonella	0.03535	0.03682	0.02912	0.02685	0.60169	1

OPP	t0 vs. t3	t0	t3	11	Johnsonella	0.00584	0.01306	0.00625	0.01466	0.93715	1
OPP	t0 vs. t3	t0	t3	11	Lachnoanaerobaculum	0.42016	0.38281	0.19925	0.11618	0.03633	1
OPP	t0 vs. t3	t0	t3	11	Lachnoclostridium	0.51627	0.58092	0.28730	0.14008	0.16407	1
OPP	t0 vs. t3	t0	t3	11	Oribacterium	0.52272	1.18214	0.15635	0.17951	0.31456	1
OPP	t0 vs. t3	t0	t3	11	Stomatobaculum	0.08171	0.14349	0.04695	0.04722	0.44878	1
OPP	t0 vs. t3	t0	t3	11	[Eubacterium]_brachy_group	0.00018	0.00060	0.00095	0.00317	0.34089	1
OPP	t0 vs. t3	t0	t3	11	[Eubacterium]_nodatum_group	0.01855	0.03836	0.05699	0.06440	0.05509	1
OPP	t0 vs. t3	t0	t3	11	Mogibacterium	0.01762	0.01814	0.04268	0.06870	0.26388	1
OPP	t0 vs. t3	t0	t3	11	[Eubacterium]_yurii_group	0.01671	0.03640	0.00698	0.01196	0.42128	1
OPP	t0 vs. t3	t0	t3	11	Peptostreptococcus	0.16686	0.16063	0.28436	0.32287	0.22143	1
OPP	t0 vs. t3	t0	t3	11	Parvimonas	0.00393	0.00654	0.00186	0.00399	0.42012	1
OPP	t0 vs. t3	t0	t3	11	Selenomonas	0.04908	0.08933	0.14738	0.28833	0.30008	1
OPP	t0 vs. t3	t0	t3	11	Dialister	0.00176	0.00372	0.00495	0.01604	0.48965	1
OPP	t0 vs. t3	t0	t3	11	Megasphaera	0.17888	0.41639	0.11944	0.14581	0.54622	1
OPP	t0 vs. t3	t0	t3	11	Veillonella	7.00433	7.10929	6.01179	3.85431	0.41821	1
OPP	t0 vs. t3	t0	t3	11	Fusobacterium	1.73244	1.48546	2.51588	1.95583	0.20579	1
OPP	t0 vs. t3	t0	t3	11	Leptotrichia	0.71107	0.96805	0.81682	1.57392	0.82280	1
OPP	t0 vs. t3	t0	t3	11	Absconditabacteriales_(SR1)	0.04217	0.09043	0.03007	0.04425	0.65692	1
OPP	t0 vs. t3	t0	t3	11	Gracilibacteria	0.00048	0.00108	0.00085	0.00204	0.58141	1
OPP	t0 vs. t3	t0	t3	11	Candidatus_Saccharimonas	0.01607	0.02548	0.06333	0.07992	0.05236	1
OPP	t0 vs. t3	t0	t3	11	Saccharimonadaceae	0.03699	0.07514	0.02045	0.03887	0.31708	1
OPP	t0 vs. t3	t0	t3	11	TM7a	0.02505	0.08159	0.02914	0.09278	0.26574	1
OPP	t0 vs. t3	t0	t3	11	TM7x	0.11247	0.10548	0.29446	0.38324	0.11890	1
OPP	t0 vs. t3	t0	t3	11	Saccharimonadales	0.10252	0.23620	0.10424	0.16362	0.98211	1
OPP	t0 vs. t3	t0	t3	11	Sphingomonas	0.00041	0.00129	0.00007	0.00024	0.42416	1
OPP	t0 vs. t3	t0	t3	11	Shewanella	0.00872	0.01140	0.00086	0.00133	0.04249	1
OPP	t0 vs. t3	t0	t3	11	Lautropia	0.08835	0.19144	0.05216	0.09131	0.47346	1
OPP	t0 vs. t3	t0	t3	11	Acidovorax	0.00669	0.01525	0.00196	0.00434	0.37037	1
OPP	t0 vs. t3	t0	t3	11	Brachymonas	0.01215	0.03503	0.00146	0.00304	0.34316	1
OPP	t0 vs. t3	t0	t3	11	Thiobacillus	0.00529	0.01630	0.00459	0.01371	0.49372	1
OPP	t0 vs. t3	t0	t3	11	Alysiella	0.00035	0.00118	0.00213	0.00706	0.34089	1
OPP	t0 vs. t3	t0	t3	11	Eikenella	0.00030	0.00099	0.00040	0.00133	0.85232	1
OPP	t0 vs. t3	t0	t3	11	Kingella	0.00377	0.01251	0.00027	0.00090	0.37987	1
OPP	t0 vs. t3	t0	t3	11	Neisseria	15.78131	12.87743	20.68155	15.09674	0.35824	1
OPP	t0 vs. t3	t0	t3	11	Simonsiella	0.00264	0.00570	0.00289	0.00641	0.91348	1
OPP	t0 vs. t3	t0	t3	11	Snodgrassella	0.00056	0.00187	0.00032	0.00106	0.34089	1
OPP	t0 vs. t3	t0	t3	11	Cardiobacterium	0.00312	0.00462	0.00689	0.01075	0.29565	1
OPP	t0 vs. t3	t0	t3	11	Serratia	0.41069	0.52832	0.31793	0.25190	0.56464	1
OPP	t0 vs. t3	t0	t3	11	Actinobacillus	0.08529	0.17046	0.09189	0.13023	0.92580	1

OPP	t0 vs. t3	t0	t3	11	Aggregatibacter	0.04777	0.06962	0.11610	0.21705	0.36415	1
OPP	t0 vs. t3	t0	t3	11	Haemophilus	4.54886	3.44327	4.62042	2.42087	0.95969	1
OPP	t0 vs. t3	t0	t3	11	Mannheimia	0.01206	0.01947	0.01445	0.02159	0.78104	1
OPP	t0 vs. t3	t0	t3	11	Pasteurella	0.63375	0.71601	0.77576	0.62897	0.67200	1
OPP	t0 vs. t3	t0	t3	11	Rodentibacter	0.10035	0.24038	0.12199	0.23334	0.84107	1
OPP	t0 vs. t3	t0	t3	11	Acinetobacter	0.24512	0.49289	0.21572	0.37989	0.74074	1
OPP	t0 vs. t3	t0	t3	11	Moraxella	2.29606	5.37342	0.65783	1.60715	0.30922	1
OPP	t0 vs. t3	t0	t3	11	Psychrobacter	0.00009	0.00030	0.00028	0.00093	0.54492	1
OPP	t0 vs. t3	t0	t3	11	Pseudomonas	0.00053	0.00175	0.00010	0.00033	0.34089	1
OPP	t0 vs. t3	t0	t3	11	Treponema	0.00263	0.00594	0.00329	0.00568	0.77999	1
OPP	t0 vs. t6	t0	t6	13	Actinomyces	1.54157	1.78004	2.07484	2.08086	0.51189	1
OPP	t0 vs. t6	t0	t6	13	Alloscardovia	0.00004	0.00014	0.21125	0.75399	0.33244	1
OPP	t0 vs. t6	t0	t6	13	Bifidobacterium	0.00014	0.00050	0.00763	0.01930	0.17804	1
OPP	t0 vs. t6	t0	t6	13	Corynebacterium	0.05390	0.09515	0.01448	0.02667	0.15229	1
OPP	t0 vs. t6	t0	t6	13	Microbacterium	0.34401	0.76996	0.31390	0.59020	0.92150	1
OPP	t0 vs. t6	t0	t6	13	Rothia	9.45984	11.65171	7.20155	6.51571	0.40444	1
OPP	t0 vs. t6	t0	t6	13	Atopobium	0.09775	0.13143	0.11395	0.13231	0.71105	1
OPP	t0 vs. t6	t0	t6	13	Bacteroides	0.02023	0.04347	0.00081	0.00200	0.13524	1
OPP	t0 vs. t6	t0	t6	13	F0058	0.00014	0.00050	0.00134	0.00272	0.12350	1
OPP	t0 vs. t6	t0	t6	13	Porphyromonas	1.11655	1.36808	1.56284	1.41591	0.35807	1
OPP	t0 vs. t6	t0	t6	13	Alloprevotella	0.80052	1.13490	0.90123	0.82049	0.74952	1
OPP	t0 vs. t6	t0	t6	13	Prevotella	11.82985	9.76757	14.92018	10.36230	0.31765	1
OPP	t0 vs. t6	t0	t6	13	Tannerella	0.00319	0.00660	0.00931	0.02099	0.35912	1
OPP	t0 vs. t6	t0	t6	13	Capnocytophaga	0.29236	0.40082	0.30405	0.35655	0.92872	1
OPP	t0 vs. t6	t0	t6	13	Flavobacterium	0.01381	0.03175	0.00121	0.00435	0.18882	1
OPP	t0 vs. t6	t0	t6	13	Bergeyella	0.00002	0.00008	0.00048	0.00172	0.36247	1
OPP	t0 vs. t6	t0	t6	13	Chryseobacterium	0.00218	0.00555	0.00583	0.01201	0.37300	1
OPP	t0 vs. t6	t0	t6	13	Campylobacter	0.65783	0.44924	1.00319	0.98807	0.32181	1
OPP	t0 vs. t6	t0	t6	13	Chloroplast	0.00052	0.00131	0.01748	0.06086	0.33631	1
OPP	t0 vs. t6	t0	t6	13	Solobacterium	0.22331	0.40849	0.13577	0.17954	0.50979	1
OPP	t0 vs. t6	t0	t6	13	Abiotrophia	0.00257	0.00457	0.00099	0.00358	0.38194	1
OPP	t0 vs. t6	t0	t6	13	Globicatella	0.00168	0.00365	0.00125	0.00449	0.80209	1
OPP	t0 vs. t6	t0	t6	13	Carnobacterium	0.03911	0.06027	0.04499	0.06854	0.82317	1
OPP	t0 vs. t6	t0	t6	13	Granulicatella	2.68905	2.12191	3.02101	1.75984	0.68909	1
OPP	t0 vs. t6	t0	t6	13	Enterococcus	0.59534	0.50648	0.39137	0.31602	0.27977	1
OPP	t0 vs. t6	t0	t6	13	Lactobacillus	0.24487	0.23777	0.27835	0.27475	0.74275	1
OPP	t0 vs. t6	t0	t6	13	Lactococcus	0.00164	0.00502	0.55485	1.99893	0.33795	1
OPP	t0 vs. t6	t0	t6	13	Streptococcus	28.73544	12.96496	23.13115	14.40218	0.34500	1
OPP	t0 vs. t6	t0	t6	13	Gemella	1.50792	1.48638	1.51759	1.03405	0.97967	1
OPP	t0 vs. t6	t0	t6	13	Staphylococcus	0.00004	0.00014	0.00086	0.00210	0.18806	1

OPP	t0 vs. t6	t0	t6	13	Clostridia_UCG-014	0.33793	0.49119	0.13988	0.26727	0.03434	1
OPP	t0 vs. t6	t0	t6	13	Defluviitaleaceae_UCG-011	0.00252	0.00907	0.00050	0.00180	0.33705	1
OPP	t0 vs. t6	t0	t6	13	Butyrivibrio	0.05098	0.11398	0.01502	0.04526	0.31553	1
OPP	t0 vs. t6	t0	t6	13	Catonella	0.27388	0.85010	0.03773	0.05801	0.34054	1
OPP	t0 vs. t6	t0	t6	13	Johnsonella	0.01407	0.03366	0.00184	0.00453	0.18141	1
OPP	t0 vs. t6	t0	t6	13	Lachnoanaerobaculum	0.67854	1.12009	0.29207	0.29595	0.22286	1
OPP	t0 vs. t6	t0	t6	13	Lachnobacterium	0.00245	0.00885	0.00214	0.00771	0.92734	1
OPP	t0 vs. t6	t0	t6	13	Lachnoclostridium	0.66105	0.87184	0.61752	0.74672	0.88870	1
OPP	t0 vs. t6	t0	t6	13	Oribacterium	0.47512	1.08887	0.15252	0.18794	0.31976	1
OPP	t0 vs. t6	t0	t6	13	Stomatobaculum	0.12642	0.22840	0.08409	0.15833	0.50590	1
OPP	t0 vs. t6	t0	t6	13	[Eubacterium]_brachy_group	0.00015	0.00055	0.00142	0.00307	0.16212	1
OPP	t0 vs. t6	t0	t6	13	[Eubacterium]_nodatum_group	0.02753	0.05191	0.07212	0.12488	0.25093	1
OPP	t0 vs. t6	t0	t6	13	Mogibacterium	0.02053	0.02339	0.09744	0.17442	0.15820	1
OPP	t0 vs. t6	t0	t6	13	[Eubacterium]_yurii_group	0.01524	0.03354	0.00727	0.01704	0.48887	1
OPP	t0 vs. t6	t0	t6	13	Peptostreptococcus	0.62456	1.68226	0.66748	0.92328	0.91030	1
OPP	t0 vs. t6	t0	t6	13	Parvimonas	0.00332	0.00615	0.00180	0.00332	0.49318	1
OPP	t0 vs. t6	t0	t6	13	Selenomonas	0.04400	0.08275	0.16347	0.40550	0.22254	1
OPP	t0 vs. t6	t0	t6	13	Dialister	0.00149	0.00346	0.00484	0.00787	0.06968	1
OPP	t0 vs. t6	t0	t6	13	Megasphaera	0.17502	0.38537	0.11821	0.18082	0.64437	1
OPP	t0 vs. t6	t0	t6	13	Veillonella	7.49805	6.66107	6.95309	2.87508	0.72847	1
OPP	t0 vs. t6	t0	t6	13	Fusobacterium	1.51802	1.46010	2.06908	1.75767	0.36344	1
OPP	t0 vs. t6	t0	t6	13	Leptotrichia	0.78098	1.01805	1.18129	1.67016	0.35558	1
OPP	t0 vs. t6	t0	t6	13	Absconditabacteriales_(SR1)	0.03614	0.08386	0.06520	0.14350	0.42661	1
OPP	t0 vs. t6	t0	t6	13	Gracilibacteria	0.00041	0.00101	0.00068	0.00149	0.43456	1
OPP	t0 vs. t6	t0	t6	13	JGI_0000069-P22	0.00007	0.00025	0.00133	0.00247	0.09669	1
OPP	t0 vs. t6	t0	t6	13	Candidatus_Saccharimonas	0.02964	0.05873	0.03263	0.04817	0.86659	1
OPP	t0 vs. t6	t0	t6	13	Saccharimonadaceae	0.03714	0.07032	0.00739	0.01027	0.15097	1
OPP	t0 vs. t6	t0	t6	13	TM7a	0.02574	0.07547	0.00143	0.00279	0.25830	1
OPP	t0 vs. t6	t0	t6	13	TM7x	0.16561	0.24705	0.47156	0.87071	0.25934	1
OPP	t0 vs. t6	t0	t6	13	Saccharimonadales	0.12453	0.24379	0.09024	0.16151	0.52780	1
OPP	t0 vs. t6	t0	t6	13	Shewanella	0.00965	0.01225	0.00161	0.00321	0.03548	1
OPP	t0 vs. t6	t0	t6	13	Lautropia	0.07565	0.17751	0.05538	0.07463	0.71670	1
OPP	t0 vs. t6	t0	t6	13	Acidovorax	0.00566	0.01415	0.00135	0.00219	0.26693	1
OPP	t0 vs. t6	t0	t6	13	Brachymonas	0.01028	0.03230	0.00130	0.00203	0.31847	1
OPP	t0 vs. t6	t0	t6	13	Thiobacillus	0.00448	0.01501	0.00026	0.00094	0.30122	1
OPP	t0 vs. t6	t0	t6	13	Alysiella	0.00030	0.00108	0.00253	0.00912	0.33705	1
OPP	t0 vs. t6	t0	t6	13	Eikenella	0.00025	0.00092	0.00064	0.00168	0.50222	1
OPP	t0 vs. t6	t0	t6	13	Kingella	0.00319	0.01151	0.00063	0.00227	0.45334	1
OPP	t0 vs. t6	t0	t6	13	Neisseria	14.69668	12.23704	17.62888	10.85642	0.51827	1

OPP	t0 vs. t6	t0	t6	13	Simonsiella	0.00285	0.00549	0.00043	0.00113	0.16028	1
OPP	t0 vs. t6	t0	t6	13	Cardiobacterium	0.00275	0.00433	0.01394	0.02675	0.15189	1
OPP	t0 vs. t6	t0	t6	13	Enterobacter	0.00017	0.00061	0.24814	0.89468	0.33739	1
OPP	t0 vs. t6	t0	t6	13	Serratia	0.35022	0.50443	0.36012	0.51942	0.96368	1
OPP	t0 vs. t6	t0	t6	13	Actinobacillus	0.07333	0.15836	0.05757	0.06922	0.59957	1
OPP	t0 vs. t6	t0	t6	13	Aggregatibacter	0.04453	0.06497	0.08259	0.07798	0.12326	1
OPP	t0 vs. t6	t0	t6	13	Haemophilus	4.13087	3.34507	4.21759	3.51482	0.89201	1
OPP	t0 vs. t6	t0	t6	13	Mannheimia	0.01121	0.01809	0.02856	0.03267	0.04599	1
OPP	t0 vs. t6	t0	t6	13	Pasteurella	0.54118	0.69171	0.73101	0.61829	0.49417	1
OPP	t0 vs. t6	t0	t6	13	Rodentibacter	0.08804	0.22164	0.13138	0.20161	0.54697	1
OPP	t0 vs. t6	t0	t6	13	Acinetobacter	0.23442	0.45636	0.14412	0.23221	0.54599	1
OPP	t0 vs. t6	t0	t6	13	Moraxella	1.94282	4.98045	0.09252	0.23135	0.20892	1
OPP	t0 vs. t6	t0	t6	13	Treponema	0.00222	0.00551	0.00042	0.00123	0.28381	1
OPP	t3 vs. t6	t3	t6	12	Actinomyces	1.99795	2.72321	2.09806	2.18570	0.89006	1
OPP	t3 vs. t6	t3	t6	12	Alloscardovia	0.02312	0.05488	0.22885	0.78472	0.36673	1
OPP	t3 vs. t6	t3	t6	12	Corynebacterium	0.00308	0.00580	0.01593	0.02735	0.07343	1
OPP	t3 vs. t6	t3	t6	12	Microbacterium	0.78736	1.51939	0.37513	0.75918	0.41567	1
OPP	t3 vs. t6	t3	t6	12	Rothia	7.92803	12.75558	7.70145	6.99944	0.93328	1
OPP	t3 vs. t6	t3	t6	12	Atopobium	0.17088	0.27879	0.11983	0.13598	0.50018	1
OPP	t3 vs. t6	t3	t6	12	Bacteroides	0.00046	0.00124	0.00088	0.00208	0.54231	1
OPP	t3 vs. t6	t3	t6	12	F0058	0.00085	0.00267	0.00089	0.00175	0.94468	1
OPP	t3 vs. t6	t3	t6	12	Porphyromonas	2.95058	3.04160	1.60511	1.47037	0.12237	1
OPP	t3 vs. t6	t3	t6	12	Alloprevotella	2.13298	3.11509	0.93403	0.85314	0.14558	1
OPP	t3 vs. t6	t3	t6	12	Prevotella	9.49463	6.74867	15.78863	10.55536	0.01070	0.9
OPP	t3 vs. t6	t3	t6	12	Tannerella	0.00419	0.01225	0.00946	0.02191	0.50850	1
OPP	t3 vs. t6	t3	t6	12	Capnocytophaga	0.31527	0.25993	0.30963	0.37467	0.96388	1
OPP	t3 vs. t6	t3	t6	12	Flavobacterium	0.00193	0.00374	0.00131	0.00453	0.68133	1
OPP	t3 vs. t6	t3	t6	12	Bergeyella	0.00008	0.00029	0.00052	0.00179	0.33880	1
OPP	t3 vs. t6	t3	t6	12	Chryseobacterium	0.00138	0.00258	0.00590	0.01255	0.22411	1
OPP	t3 vs. t6	t3	t6	12	Campylobacter	1.05006	0.92899	1.17897	1.37046	0.79832	1
OPP	t3 vs. t6	t3	t6	12	Chloroplast	0.00149	0.00349	0.02811	0.06863	0.18551	1
OPP	t3 vs. t6	t3	t6	12	Solobacterium	0.10786	0.09871	0.10588	0.12696	0.96470	1
OPP	t3 vs. t6	t3	t6	12	Abiotrophia	0.00093	0.00320	0.00108	0.00372	0.92112	1
OPP	t3 vs. t6	t3	t6	12	Globicatella	0.00341	0.00787	0.00135	0.00468	0.47715	1
OPP	t3 vs. t6	t3	t6	12	Carnobacterium	0.01801	0.03150	0.02845	0.05086	0.55953	1
OPP	t3 vs. t6	t3	t6	12	Granulicatella	3.35477	1.92443	2.62533	1.25933	0.19982	1
OPP	t3 vs. t6	t3	t6	12	Enterococcus	0.49275	0.34506	0.33337	0.20859	0.23055	1
OPP	t3 vs. t6	t3	t6	12	Lactobacillus	0.23462	0.19795	0.21493	0.14187	0.76204	1
OPP	t3 vs. t6	t3	t6	12	Leuconostoc	0.00030	0.00080	0.00190	0.00658	0.42723	1
OPP	t3 vs. t6	t3	t6	12	Lactococcus	0.01681	0.05327	0.60405	2.07964	0.35027	1

OPP	t3 vs. t6	t3	t6	12	Streptococcus	24.35667	14.57217	22.61007	15.15912	0.77255	1
OPP	t3 vs. t6	t3	t6	12	Gemella	1.83925	1.20819	1.56012	1.12205	0.35158	1
OPP	t3 vs. t6	t3	t6	12	Staphylococcus	0.00027	0.00066	0.00110	0.00262	0.19203	1
OPP	t3 vs. t6	t3	t6	12	Clostridia_UCG-014	0.38467	0.73574	0.05834	0.09756	0.15303	1
OPP	t3 vs. t6	t3	t6	12	Butyrivibrio	0.06448	0.20140	0.01358	0.04705	0.42013	1
OPP	t3 vs. t6	t3	t6	12	Catonella	0.03018	0.02586	0.05677	0.09458	0.33987	1
OPP	t3 vs. t6	t3	t6	12	Johnsonella	0.00573	0.01409	0.00112	0.00387	0.16999	1
OPP	t3 vs. t6	t3	t6	12	Lachnoanaerobaculum	0.45720	0.90042	0.28663	0.30041	0.55803	1
OPP	t3 vs. t6	t3	t6	12	Lachnoclostridium	0.34793	0.24891	0.57727	0.62975	0.09899	1
OPP	t3 vs. t6	t3	t6	12	Oribacterium	0.33283	0.63488	0.18841	0.23141	0.38374	1
OPP	t3 vs. t6	t3	t6	12	Stomatobaculum	0.07835	0.11774	0.10567	0.16664	0.59098	1
OPP	t3 vs. t6	t3	t6	12	[Eubacterium]_brachy_group	0.00088	0.00303	0.00093	0.00205	0.95207	1
OPP	t3 vs. t6	t3	t6	12	[Eubacterium]_nodatum_group	0.05241	0.06342	0.04098	0.05525	0.41512	1
OPP	t3 vs. t6	t3	t6	12	Mogibacterium	0.04027	0.06603	0.08855	0.15338	0.17248	1
OPP	t3 vs. t6	t3	t6	12	[Eubacterium]_yurii_group	0.00640	0.01158	0.00222	0.00440	0.18456	1
OPP	t3 vs. t6	t3	t6	12	Filifactor	0.00043	0.00147	0.00683	0.02364	0.37199	1
OPP	t3 vs. t6	t3	t6	12	Peptostreptococcus	0.26088	0.31841	0.50608	0.78006	0.34785	1
OPP	t3 vs. t6	t3	t6	12	Parvimonas	0.00171	0.00384	0.00134	0.00253	0.60560	1
OPP	t3 vs. t6	t3	t6	12	Selenomonas	0.13708	0.27722	0.17411	0.42159	0.81026	1
OPP	t3 vs. t6	t3	t6	12	Dialister	0.00453	0.01536	0.00404	0.00710	0.88671	1
OPP	t3 vs. t6	t3	t6	12	Megasphaera	0.10948	0.14324	0.13335	0.18228	0.73946	1
OPP	t3 vs. t6	t3	t6	12	Veillonella	5.59413	3.94948	6.88742	2.85806	0.24030	1
OPP	t3 vs. t6	t3	t6	12	Fusobacterium	2.33276	1.96975	2.01031	1.83255	0.54198	1
OPP	t3 vs. t6	t3	t6	12	Leptotrichia	0.78158	1.50563	1.11512	1.72517	0.64634	1
OPP	t3 vs. t6	t3	t6	12	Streptobacillus	0.00004	0.00014	0.00076	0.00143	0.11690	1
OPP	t3 vs. t6	t3	t6	12	Absconditabacteriales_(SR1)	0.02757	0.04308	0.07009	0.14875	0.36420	1
OPP	t3 vs. t6	t3	t6	12	Gracilibacteria	0.00078	0.00196	0.00048	0.00134	0.54135	1
OPP	t3 vs. t6	t3	t6	12	JGI_0000069-P22	0.00093	0.00187	0.00113	0.00247	0.81309	1
OPP	t3 vs. t6	t3	t6	12	Candidatus_Saccharimonas	0.05834	0.07813	0.02474	0.04834	0.07937	1
OPP	t3 vs. t6	t3	t6	12	Saccharimonadaceae	0.01875	0.03753	0.00368	0.00464	0.19764	1
OPP	t3 vs. t6	t3	t6	12	TM7a	0.02671	0.08886	0.00155	0.00288	0.33846	1
OPP	t3 vs. t6	t3	t6	12	TM7x	0.27073	0.37455	0.30725	0.70788	0.86919	1
OPP	t3 vs. t6	t3	t6	12	Saccharimonadales	0.09555	0.15888	0.06603	0.15541	0.63176	1
OPP	t3 vs. t6	t3	t6	12	Paracoccus	0.00008	0.00026	0.00022	0.00075	0.55988	1
OPP	t3 vs. t6	t3	t6	12	Shewanella	0.00079	0.00129	0.00120	0.00298	0.68123	1
OPP	t3 vs. t6	t3	t6	12	Lautropia	0.04818	0.08815	0.04955	0.07827	0.96786	1
OPP	t3 vs. t6	t3	t6	12	Acidovorax	0.00180	0.00417	0.00147	0.00224	0.73946	1
OPP	t3 vs. t6	t3	t6	12	Brachymonas	0.00134	0.00293	0.00141	0.00208	0.94961	1
OPP	t3 vs. t6	t3	t6	12	Thiobacillus	0.00421	0.01314	0.00028	0.00098	0.28746	1

OPP	t3 vs. t6	t3	t6	12	Alysiella	0.00195	0.00675	0.00274	0.00950	0.33880	1
OPP	t3 vs. t6	t3	t6	12	Eikenella	0.00037	0.00127	0.00069	0.00175	0.19105	1
OPP	t3 vs. t6	t3	t6	12	Kingella	0.00025	0.00087	0.00068	0.00237	0.57438	1
OPP	t3 vs. t6	t3	t6	12	Neisseria	19.43788	15.02507	17.46754	10.88118	0.51473	1
OPP	t3 vs. t6	t3	t6	12	Simonsiella	0.00265	0.00617	0.00047	0.00117	0.22083	1
OPP	t3 vs. t6	t3	t6	12	Cardiobacterium	0.00638	0.01041	0.01423	0.02784	0.15752	1
OPP	t3 vs. t6	t3	t6	12	Escherichia-Shigella	0.00048	0.00167	0.00009	0.00032	0.45003	1
OPP	t3 vs. t6	t3	t6	12	Serratia	0.29143	0.25712	0.24680	0.37458	0.75364	1
OPP	t3 vs. t6	t3	t6	12	Actinobacillus	0.08448	0.12680	0.05525	0.07343	0.47014	1
OPP	t3 vs. t6	t3	t6	12	Aggregatibacter	0.10643	0.20965	0.06824	0.06457	0.50251	1
OPP	t3 vs. t6	t3	t6	12	Haemophilus	4.37219	2.46318	3.99954	3.61652	0.77672	1
OPP	t3 vs. t6	t3	t6	12	Mannheimia	0.01324	0.02100	0.02203	0.02860	0.36653	1
OPP	t3 vs. t6	t3	t6	12	Pasteurella	0.72961	0.62064	0.66059	0.59234	0.76121	1
OPP	t3 vs. t6	t3	t6	12	Rodentibacter	0.11183	0.22525	0.13423	0.21099	0.78212	1
OPP	t3 vs. t6	t3	t6	12	Acinetobacter	0.19820	0.36726	0.13275	0.23895	0.53736	1
OPP	t3 vs. t6	t3	t6	12	Moraxella	0.60301	1.54407	0.10011	0.23994	0.28040	1
OPP	t3 vs. t6	t3	t6	12	Treponema	0.00302	0.00549	0.00045	0.00128	0.12822	1
OPP	t3 vs. t6	t3	t6	12	Fretibacterium	0.00022	0.00075	0.00003	0.00012	0.42684	1
TAU	t0 vs. t3	t0	t3	15	Actinomyces	3.05770	2.67200	2.74669	2.66049	0.70870	1
TAU	t0 vs. t3	t0	t3	15	Actinotignum	0.00061	0.00149	0.00337	0.01263	0.37135	1
TAU	t0 vs. t3	t0	t3	15	Arcanobacterium	0.00664	0.02572	0.00199	0.00772	0.52156	1
TAU	t0 vs. t3	t0	t3	15	F0332	0.00245	0.00496	0.01553	0.05922	0.37487	1
TAU	t0 vs. t3	t0	t3	15	Mobiluncus	0.00024	0.00093	0.00163	0.00630	0.33428	1
TAU	t0 vs. t3	t0	t3	15	Alloscardovia	0.00204	0.00790	0.00323	0.00951	0.11119	1
TAU	t0 vs. t3	t0	t3	15	Bifidobacterium	0.00647	0.02507	0.00097	0.00377	0.33428	1
TAU	t0 vs. t3	t0	t3	15	Scardovia	0.00055	0.00163	0.00035	0.00096	0.70026	1
TAU	t0 vs. t3	t0	t3	15	Corynebacterium	0.02758	0.04951	0.01789	0.03288	0.52300	1
TAU	t0 vs. t3	t0	t3	15	Microbacterium	1.37536	1.60929	1.02731	1.70127	0.43898	1
TAU	t0 vs. t3	t0	t3	15	Micrococcus	0.00457	0.01771	0.00337	0.01306	0.33428	1
TAU	t0 vs. t3	t0	t3	15	Rothia	5.64679	6.33970	3.47354	2.95560	0.18991	1
TAU	t0 vs. t3	t0	t3	15	Pseudopropionibacterium	0.00001	0.00005	0.00005	0.00021	0.48596	1
TAU	t0 vs. t3	t0	t3	15	Tessaracoccus	0.00012	0.00034	0.00006	0.00023	0.59510	1
TAU	t0 vs. t3	t0	t3	15	Atopobium	0.34968	0.41259	0.22466	0.32378	0.30799	1
TAU	t0 vs. t3	t0	t3	15	Olsenella	0.00014	0.00054	0.00003	0.00013	0.47770	1
TAU	t0 vs. t3	t0	t3	15	Cryptobacterium	0.00015	0.00059	0.00007	0.00028	0.33428	1
TAU	t0 vs. t3	t0	t3	15	Slackia	0.00045	0.00176	0.00184	0.00713	0.33428	1
TAU	t0 vs. t3	t0	t3	15	Bacteroides	0.00703	0.00957	0.00537	0.01547	0.74609	1
TAU	t0 vs. t3	t0	t3	15	Phocaeicola	0.00009	0.00036	0.00013	0.00049	0.33428	1
TAU	t0 vs. t3	t0	t3	15	F0058	0.00123	0.00405	0.00109	0.00381	0.91934	1
TAU	t0 vs. t3	t0	t3	15	Porphyromonas	1.44685	2.01786	2.07531	2.16193	0.28016	1

TAU	t0 vs. t3	t0	t3	15	Alloprevotella	1.23803	1.38406	1.18373	1.22828	0.81636	1
TAU	t0 vs. t3	t0	t3	15	Prevotella	14.15345	10.73582	15.57652	12.42542	0.65113	1
TAU	t0 vs. t3	t0	t3	15	Rikenellaceae_RC9_gut_group	0.00099	0.00287	0.00335	0.00885	0.18347	1
TAU	t0 vs. t3	t0	t3	15	Tannerella	0.02235	0.04359	0.00366	0.00517	0.12634	1
TAU	t0 vs. t3	t0	t3	15	Capnocytophaga	0.58105	0.95315	0.26911	0.26062	0.13095	1
TAU	t0 vs. t3	t0	t3	15	Flavobacterium	0.00246	0.00537	0.00577	0.01669	0.48906	1
TAU	t0 vs. t3	t0	t3	15	Bergeyella	0.00121	0.00403	0.00041	0.00111	0.47060	1
TAU	t0 vs. t3	t0	t3	15	Chryseobacterium	0.00947	0.02851	0.00130	0.00271	0.29475	1
TAU	t0 vs. t3	t0	t3	15	Lentimicrobium	0.00017	0.00062	0.00133	0.00514	0.33716	1
TAU	t0 vs. t3	t0	t3	15	Campylobacter	1.21065	0.97887	0.79425	0.85352	0.14798	1
TAU	t0 vs. t3	t0	t3	15	Chloroplast	0.01151	0.02804	0.00149	0.00416	0.14738	1
TAU	t0 vs. t3	t0	t3	15	Desulfovibrio	0.00007	0.00026	0.00046	0.00178	0.33428	1
TAU	t0 vs. t3	t0	t3	15	Solobacterium	0.33289	0.31141	0.22138	0.20415	0.16646	1
TAU	t0 vs. t3	t0	t3	15	Abiotrophia	0.00191	0.00551	0.00324	0.00858	0.64250	1
TAU	t0 vs. t3	t0	t3	15	Globicatella	0.00131	0.00355	0.01091	0.03590	0.32530	1
TAU	t0 vs. t3	t0	t3	15	Alkalibacterium	0.00212	0.00428	0.00063	0.00180	0.20780	1
TAU	t0 vs. t3	t0	t3	15	Carnobacterium	0.02801	0.03329	0.04907	0.08134	0.29513	1
TAU	t0 vs. t3	t0	t3	15	Granulicatella	2.83141	1.62342	3.32947	2.70443	0.35824	1
TAU	t0 vs. t3	t0	t3	15	Trichococcus	0.03215	0.12450	0.00272	0.01053	0.37990	1
TAU	t0 vs. t3	t0	t3	15	Enterococcus	0.54931	0.33830	0.64962	0.50598	0.45813	1
TAU	t0 vs. t3	t0	t3	15	Lactobacillus	0.42669	0.30830	0.41382	0.30388	0.88470	1
TAU	t0 vs. t3	t0	t3	15	Streptococcus	23.64317	5.61679	25.67657	15.74263	0.63955	1
TAU	t0 vs. t3	t0	t3	15	Mycoplasma	0.00067	0.00188	0.00089	0.00218	0.69222	1
TAU	t0 vs. t3	t0	t3	15	RF39	0.00109	0.00421	0.00221	0.00591	0.25581	1
TAU	t0 vs. t3	t0	t3	15	Gemella	2.01461	1.75097	2.18127	1.77757	0.67672	1
TAU	t0 vs. t3	t0	t3	15	Staphylococcus	0.03465	0.13286	0.00160	0.00555	0.35370	1
TAU	t0 vs. t3	t0	t3	15	Clostridia_UCG-014	0.35111	0.53189	0.12115	0.09330	0.13263	1
TAU	t0 vs. t3	t0	t3	15	Defluviitaleaceae_UCG-011	0.00317	0.00694	0.00425	0.01145	0.73180	1
TAU	t0 vs. t3	t0	t3	15	Butyrivibrio	0.25145	0.68336	0.09751	0.26616	0.36858	1
TAU	t0 vs. t3	t0	t3	15	Catonella	0.08139	0.12874	0.06221	0.09016	0.23800	1
TAU	t0 vs. t3	t0	t3	15	Howardella	0.00025	0.00096	0.00294	0.01139	0.33428	1
TAU	t0 vs. t3	t0	t3	15	Johnsonella	0.00784	0.01210	0.00295	0.00607	0.11454	1
TAU	t0 vs. t3	t0	t3	15	Lachnoanaerobaculum	0.45399	0.60812	0.36338	0.44652	0.59187	1
TAU	t0 vs. t3	t0	t3	15	Lachnoclostridium	0.52651	0.64395	0.35563	0.35085	0.36549	1
TAU	t0 vs. t3	t0	t3	15	Oribacterium	0.37387	0.36258	0.29039	0.34630	0.46921	1
TAU	t0 vs. t3	t0	t3	15	Shuttleworthia	0.00083	0.00320	0.00777	0.02726	0.28306	1
TAU	t0 vs. t3	t0	t3	15	Stomatobaculum	0.11859	0.16148	0.13966	0.22504	0.74221	1
TAU	t0 vs. t3	t0	t3	15	[Eubacterium]_brachy_group	0.00289	0.01015	0.00119	0.00400	0.55192	1
TAU	t0 vs. t3	t0	t3	15	[Eubacterium]_nodatum_group	0.15225	0.17796	0.12716	0.18468	0.70936	1

TAU	t0 vs. t3	t0	t3	15	[Eubacterium]_saphenum_group	0.00003	0.00013	0.00405	0.01570	0.33428	1
TAU	t0 vs. t3	t0	t3	15	Amnipila	0.00428	0.01658	0.00490	0.01898	0.33428	1
TAU	t0 vs. t3	t0	t3	15	Family_XIII_UCG-001	0.00767	0.02950	0.01363	0.04011	0.59379	1
TAU	t0 vs. t3	t0	t3	15	Mogibacterium	0.04071	0.04486	0.05748	0.08015	0.43449	1
TAU	t0 vs. t3	t0	t3	15	[Eubacterium]_yurii_group	0.03351	0.11510	0.02121	0.04249	0.58952	1
TAU	t0 vs. t3	t0	t3	15	Filifactor	0.01585	0.03485	0.00580	0.01625	0.20910	1
TAU	t0 vs. t3	t0	t3	15	Peptoanaerobacter	0.00053	0.00207	0.00017	0.00065	0.33428	1
TAU	t0 vs. t3	t0	t3	15	Peptostreptococcus	0.36249	0.52010	0.40992	0.57721	0.70554	1
TAU	t0 vs. t3	t0	t3	15	Parvimonas	0.00767	0.01326	0.01093	0.01598	0.40342	1
TAU	t0 vs. t3	t0	t3	15	Selenomonas	0.20819	0.24757	0.18236	0.28581	0.74560	1
TAU	t0 vs. t3	t0	t3	15	Dialister	0.34954	1.20216	0.25515	0.91948	0.81416	1
TAU	t0 vs. t3	t0	t3	15	Megasphaera	0.37109	0.59796	0.39097	1.04731	0.91663	1
TAU	t0 vs. t3	t0	t3	15	Veillonella	7.12994	2.75904	6.56421	3.55100	0.48810	1
TAU	t0 vs. t3	t0	t3	15	Fusobacterium	1.94793	1.27948	2.52726	2.03379	0.24555	1
TAU	t0 vs. t3	t0	t3	15	Leptotrichia	0.94670	1.44067	0.52774	0.45982	0.33229	1
TAU	t0 vs. t3	t0	t3	15	Streptobacillus	0.00083	0.00218	0.00006	0.00023	0.16447	1
TAU	t0 vs. t3	t0	t3	15	Absconditabacteriales_(SR1)	0.04058	0.07958	0.08537	0.13519	0.10577	1
TAU	t0 vs. t3	t0	t3	15	Gracilibacteria	0.00010	0.00039	0.00022	0.00085	0.63606	1
TAU	t0 vs. t3	t0	t3	15	JGI_0000069-P22	0.00009	0.00036	0.00053	0.00133	0.12059	1
TAU	t0 vs. t3	t0	t3	15	Candidatus_Saccharimonas	0.10819	0.13933	0.08211	0.08051	0.43611	1
TAU	t0 vs. t3	t0	t3	15	Saccharimonadaceae	0.05457	0.06421	0.02046	0.02312	0.03470	1
TAU	t0 vs. t3	t0	t3	15	TM7a	0.00041	0.00088	0.00074	0.00191	0.54968	1
TAU	t0 vs. t3	t0	t3	15	TM7x	0.51234	0.59635	0.32881	0.48647	0.06953	1
TAU	t0 vs. t3	t0	t3	15	Saccharimonadales	0.24673	0.33274	0.07706	0.10271	0.02266	1
TAU	t0 vs. t3	t0	t3	15	Sphingomonas	0.00030	0.00116	0.00002	0.00008	0.36969	1
TAU	t0 vs. t3	t0	t3	15	Shewanella	0.00772	0.01012	0.00516	0.00883	0.45597	1
TAU	t0 vs. t3	t0	t3	15	Lautropia	0.13047	0.33169	0.11633	0.15640	0.88668	1
TAU	t0 vs. t3	t0	t3	15	Acidovorax	0.00273	0.00696	0.00101	0.00260	0.31022	1
TAU	t0 vs. t3	t0	t3	15	Brachymonas	0.00281	0.00739	0.00103	0.00177	0.38363	1
TAU	t0 vs. t3	t0	t3	15	Thiobacillus	0.02409	0.06315	0.01162	0.02646	0.47899	1
TAU	t0 vs. t3	t0	t3	15	Alysiella	0.00166	0.00643	0.00194	0.00676	0.11086	1
TAU	t0 vs. t3	t0	t3	15	Eikenella	0.00003	0.00010	0.00063	0.00130	0.10019	1
TAU	t0 vs. t3	t0	t3	15	Kingella	0.00183	0.00574	0.01039	0.03599	0.29412	1
TAU	t0 vs. t3	t0	t3	15	Neisseria	17.70122	10.60723	15.39917	9.56546	0.17134	1
TAU	t0 vs. t3	t0	t3	15	Simonsiella	0.00674	0.01784	0.00055	0.00144	0.17628	1
TAU	t0 vs. t3	t0	t3	15	Cardiobacterium	0.01145	0.01138	0.00987	0.01365	0.62660	1
TAU	t0 vs. t3	t0	t3	15	Serratia	0.33757	0.51756	0.57851	0.71501	0.06621	1
TAU	t0 vs. t3	t0	t3	15	Actinobacillus	0.06689	0.09328	0.07910	0.11353	0.73627	1
TAU	t0 vs. t3	t0	t3	15	Aggregatibacter	0.11180	0.14293	0.13277	0.19873	0.73388	1
TAU	t0 vs. t3	t0	t3	15	Haemophilus	2.72905	2.05159	3.85777	2.36726	0.01332	1

TAU	t0 vs. t3	t0	t3	15	Mannheimia	0.02773	0.03565	0.04915	0.09055	0.35872	1
TAU	t0 vs. t3	t0	t3	15	Pasteurella	0.44930	0.51132	0.80641	0.75334	0.08964	1
TAU	t0 vs. t3	t0	t3	15	Rodentibacter	0.05953	0.13952	0.14477	0.29026	0.34164	1
TAU	t0 vs. t3	t0	t3	15	Acinetobacter	0.15888	0.25446	0.08486	0.18107	0.39230	1
TAU	t0 vs. t3	t0	t3	15	Moraxella	0.33473	1.01409	0.47781	1.15161	0.24867	1
TAU	t0 vs. t3	t0	t3	15	Psychrobacter	0.00008	0.00031	0.00003	0.00010	0.54565	1
TAU	t0 vs. t3	t0	t3	15	Pseudomonas	0.00002	0.00008	0.00005	0.00014	0.23767	1
TAU	t0 vs. t3	t0	t3	15	Vibrio	0.00011	0.00044	0.00009	0.00034	0.85913	1
TAU	t0 vs. t3	t0	t3	15	Treponema	0.00414	0.00797	0.00600	0.01265	0.35114	1
TAU	t0 vs. t3	t0	t3	15	Fretibacterium	0.00093	0.00268	0.00597	0.02240	0.34487	1
TAU	t0 vs. t6	t0	t6	14	Actinomyces	2.99526	2.76148	3.53947	3.22755	0.37449	1
TAU	t0 vs. t6	t0	t6	14	Actinotignum	0.00066	0.00154	0.00010	0.00037	0.21021	1
TAU	t0 vs. t6	t0	t6	14	F0332	0.00262	0.00510	0.00086	0.00323	0.05457	1
TAU	t0 vs. t6	t0	t6	14	Alloscardovia	0.00219	0.00818	0.02313	0.04915	0.12682	1
TAU	t0 vs. t6	t0	t6	14	Bifidobacterium	0.00694	0.02595	0.00432	0.01617	0.33556	1
TAU	t0 vs. t6	t0	t6	14	Scardovia	0.00059	0.00168	0.00016	0.00046	0.22680	1
TAU	t0 vs. t6	t0	t6	14	Corynebacterium	0.02955	0.05076	0.02589	0.06460	0.87127	1
TAU	t0 vs. t6	t0	t6	14	Microbacterium	1.45718	1.63734	1.05506	1.49896	0.28641	1
TAU	t0 vs. t6	t0	t6	14	Rothia	5.99849	6.42538	5.61551	4.43608	0.81686	1
TAU	t0 vs. t6	t0	t6	14	Pseudopropionibacterium	0.00001	0.00005	0.00015	0.00056	0.33556	1
TAU	t0 vs. t6	t0	t6	14	Tessaracoccus	0.00013	0.00035	0.00056	0.00175	0.34870	1
TAU	t0 vs. t6	t0	t6	14	Atopobium	0.30851	0.39489	0.13377	0.15258	0.06674	1
TAU	t0 vs. t6	t0	t6	14	Cryptobacterium	0.00016	0.00061	0.00083	0.00310	0.33556	1
TAU	t0 vs. t6	t0	t6	14	Bacteroides	0.00754	0.00973	0.00042	0.00112	0.02039	1
TAU	t0 vs. t6	t0	t6	14	F0058	0.00132	0.00419	0.00509	0.01077	0.24269	1
TAU	t0 vs. t6	t0	t6	14	Porphyromonas	1.54341	2.05776	1.69109	1.32739	0.73043	1
TAU	t0 vs. t6	t0	t6	14	Alloprevotella	1.10957	1.34031	1.30732	1.35592	0.61501	1
TAU	t0 vs. t6	t0	t6	14	Prevotella	12.48612	8.90037	15.60541	12.67606	0.26821	1
TAU	t0 vs. t6	t0	t6	14	Rikenellaceae_RC9_gut_group	0.00106	0.00297	0.00017	0.00046	0.28634	1
TAU	t0 vs. t6	t0	t6	14	Tannerella	0.02291	0.04518	0.03019	0.05321	0.64950	1
TAU	t0 vs. t6	t0	t6	14	Capnocytophaga	0.61982	0.97678	0.45914	0.49981	0.51361	1
TAU	t0 vs. t6	t0	t6	14	Flavobacterium	0.00264	0.00553	0.00456	0.01089	0.28296	1
TAU	t0 vs. t6	t0	t6	14	Bergeyella	0.00129	0.00417	0.00145	0.00335	0.91942	1
TAU	t0 vs. t6	t0	t6	14	Chryseobacterium	0.00979	0.02955	0.00910	0.01703	0.93946	1
TAU	t0 vs. t6	t0	t6	14	Lentimicrobium	0.00018	0.00064	0.00019	0.00072	0.95739	1
TAU	t0 vs. t6	t0	t6	14	Campylobacter	1.21346	1.01576	1.27889	0.89383	0.64395	1
TAU	t0 vs. t6	t0	t6	14	Chloroplast	0.01234	0.02891	0.03439	0.08627	0.39633	1
TAU	t0 vs. t6	t0	t6	14	Solobacterium	0.29929	0.29360	0.26916	0.40133	0.69260	1
TAU	t0 vs. t6	t0	t6	14	Abiotrophia	0.00205	0.00569	0.00464	0.01735	0.61561	1

TAU	t0 vs. t6	t0	t6	14	Globicatella	0.00140	0.00366	0.00350	0.01012	0.48190	1
TAU	t0 vs. t6	t0	t6	14	Alkalibacterium	0.00227	0.00440	0.00005	0.00019	0.08352	1
TAU	t0 vs. t6	t0	t6	14	Carnobacterium	0.03001	0.03360	0.01859	0.04446	0.31756	1
TAU	t0 vs. t6	t0	t6	14	Granulicatella	2.94584	1.62072	2.79156	1.80983	0.64301	1
TAU	t0 vs. t6	t0	t6	14	Enterococcus	0.57604	0.33423	0.48815	0.40923	0.27291	1
TAU	t0 vs. t6	t0	t6	14	Lactobacillus	0.45415	0.30030	0.49028	0.71654	0.83958	1
TAU	t0 vs. t6	t0	t6	14	Lactococcus	0.01307	0.04877	0.00066	0.00246	0.36059	1
TAU	t0 vs. t6	t0	t6	14	Streptococcus	23.69741	5.82474	18.70532	5.72178	0.04499	1
TAU	t0 vs. t6	t0	t6	14	Mycoplasma	0.00072	0.00195	0.00036	0.00077	0.55227	1
TAU	t0 vs. t6	t0	t6	14	RF39	0.00116	0.00436	0.00374	0.01201	0.23522	1
TAU	t0 vs. t6	t0	t6	14	Gemella	2.13681	1.74943	2.12580	1.19554	0.97850	1
TAU	t0 vs. t6	t0	t6	14	Staphylococcus	0.03712	0.13752	0.00007	0.00027	0.33189	1
TAU	t0 vs. t6	t0	t6	14	Clostridia_UCG-014	0.34932	0.55192	0.39116	0.55084	0.84265	1
TAU	t0 vs. t6	t0	t6	14	Defluviitaleaceae_UCG-011	0.00304	0.00718	0.00658	0.02427	0.48472	1
TAU	t0 vs. t6	t0	t6	14	Butyrivibrio	0.25650	0.70887	0.10309	0.26113	0.25116	1
TAU	t0 vs. t6	t0	t6	14	Catonella	0.08654	0.13199	0.05061	0.08685	0.26132	1
TAU	t0 vs. t6	t0	t6	14	Howardella	0.00026	0.00099	0.00276	0.00951	0.29391	1
TAU	t0 vs. t6	t0	t6	14	Johnsonella	0.00811	0.01250	0.02020	0.07316	0.49998	1
TAU	t0 vs. t6	t0	t6	14	Lachnoanaerobaculum	0.47481	0.62550	0.24856	0.20446	0.18631	1
TAU	t0 vs. t6	t0	t6	14	Lachnoclostridium	0.53990	0.66609	0.32611	0.22958	0.25883	1
TAU	t0 vs. t6	t0	t6	14	Oribacterium	0.38591	0.37314	0.21577	0.15645	0.06163	1
TAU	t0 vs. t6	t0	t6	14	Shuttleworthia	0.00089	0.00331	0.00134	0.00412	0.16683	1
TAU	t0 vs. t6	t0	t6	14	Stomatobaculum	0.11441	0.16673	0.05622	0.05987	0.17091	1
TAU	t0 vs. t6	t0	t6	14	[Eubacterium]_brachy_group	0.00310	0.01050	0.00026	0.00068	0.33339	1
TAU	t0 vs. t6	t0	t6	14	[Eubacterium]_nodatum_group	0.15843	0.18300	0.13159	0.18797	0.73587	1
TAU	t0 vs. t6	t0	t6	14	Amnipila	0.00459	0.01716	0.00350	0.01248	0.40618	1
TAU	t0 vs. t6	t0	t6	14	Family_XIII_UCG-001	0.00822	0.03053	0.00146	0.00548	0.33132	1
TAU	t0 vs. t6	t0	t6	14	Mogibacterium	0.04231	0.04610	0.04076	0.04781	0.92844	1
TAU	t0 vs. t6	t0	t6	14	[Eubacterium]_yurii_group	0.03590	0.11906	0.01426	0.02546	0.52761	1
TAU	t0 vs. t6	t0	t6	14	Filifactor	0.01691	0.03591	0.00355	0.01277	0.12616	1
TAU	t0 vs. t6	t0	t6	14	Peptostreptococcus	0.38456	0.53239	0.16984	0.15790	0.16203	1
TAU	t0 vs. t6	t0	t6	14	Parvimonas	0.00821	0.01359	0.00683	0.01295	0.77176	1
TAU	t0 vs. t6	t0	t6	14	Selenomonas	0.21266	0.25629	0.26716	0.55617	0.72599	1
TAU	t0 vs. t6	t0	t6	14	Anaeroglobus	0.00030	0.00112	0.00012	0.00045	0.59996	1
TAU	t0 vs. t6	t0	t6	14	Dialister	0.37451	1.24350	0.05711	0.12504	0.31842	1
TAU	t0 vs. t6	t0	t6	14	Megasphaera	0.36499	0.62005	0.28249	0.39747	0.54092	1
TAU	t0 vs. t6	t0	t6	14	Veillonella	6.94386	2.76379	6.08570	4.07698	0.32159	1
TAU	t0 vs. t6	t0	t6	14	Fusobacterium	1.97632	1.32287	2.56707	1.76084	0.24788	1
TAU	t0 vs. t6	t0	t6	14	Leptotrichia	0.97039	1.49202	1.18114	2.08977	0.69545	1
TAU	t0 vs. t6	t0	t6	14	Streptobacillus	0.00089	0.00225	0.00004	0.00016	0.18958	1

TAU	t0 vs. t6	t0	t6	14	Absconditabacteriales_(SR1)	0.04348	0.08176	0.12134	0.30848	0.30470	1
TAU	t0 vs. t6	t0	t6	14	Gracilibacteria	0.00011	0.00040	0.00049	0.00099	0.09971	1
TAU	t0 vs. t6	t0	t6	14	JGI_0000069-P22	0.00010	0.00037	0.00059	0.00176	0.21780	1
TAU	t0 vs. t6	t0	t6	14	Candidatus_Saccharimonas	0.11009	0.14439	0.14139	0.31809	0.59719	1
TAU	t0 vs. t6	t0	t6	14	Saccharimonadaceae	0.05812	0.06509	0.03079	0.07408	0.10769	1
TAU	t0 vs. t6	t0	t6	14	TM7a	0.00044	0.00090	0.00031	0.00082	0.70971	1
TAU	t0 vs. t6	t0	t6	14	TM7x	0.45559	0.57529	0.51556	0.72738	0.78598	1
TAU	t0 vs. t6	t0	t6	14	Saccharimonadales	0.26337	0.33875	0.22759	0.44821	0.66913	1
TAU	t0 vs. t6	t0	t6	14	Caulobacter	0.00001	0.00003	0.00035	0.00131	0.34599	1
TAU	t0 vs. t6	t0	t6	14	Mitochondria	0.00009	0.00035	0.00033	0.00066	0.15225	1
TAU	t0 vs. t6	t0	t6	14	Sphingomonas	0.00032	0.00120	0.00009	0.00035	0.51484	1
TAU	t0 vs. t6	t0	t6	14	Shewanella	0.00827	0.01026	0.00084	0.00174	0.02100	1
TAU	t0 vs. t6	t0	t6	14	Lautropia	0.13914	0.34244	0.23243	0.52903	0.13445	1
TAU	t0 vs. t6	t0	t6	14	Acidovorax	0.00292	0.00718	0.00532	0.01707	0.53235	1
TAU	t0 vs. t6	t0	t6	14	Brachymonas	0.00301	0.00762	0.00426	0.01471	0.72917	1
TAU	t0 vs. t6	t0	t6	14	Thiobacillus	0.02581	0.06517	0.00079	0.00225	0.17279	1
TAU	t0 vs. t6	t0	t6	14	Alysiella	0.00178	0.00665	0.00137	0.00456	0.50087	1
TAU	t0 vs. t6	t0	t6	14	Eikenella	0.00003	0.00011	0.00026	0.00096	0.39688	1
TAU	t0 vs. t6	t0	t6	14	Kingella	0.00196	0.00594	0.00301	0.01091	0.76587	1
TAU	t0 vs. t6	t0	t6	14	Neisseria	18.50338	10.52491	18.43358	10.45391	0.97575	1
TAU	t0 vs. t6	t0	t6	14	Simonsiella	0.00563	0.01797	0.00555	0.01671	0.88021	1
TAU	t0 vs. t6	t0	t6	14	Cardiobacterium	0.01226	0.01135	0.01455	0.02053	0.71840	1
TAU	t0 vs. t6	t0	t6	14	Serratia	0.35827	0.53061	0.45617	0.45175	0.51349	1
TAU	t0 vs. t6	t0	t6	14	Actinobacillus	0.07166	0.09488	0.10369	0.10321	0.16456	1
TAU	t0 vs. t6	t0	t6	14	Aggregatibacter	0.11929	0.14524	0.14927	0.32808	0.67802	1
TAU	t0 vs. t6	t0	t6	14	Haemophilus	2.88491	2.03478	4.93179	2.09861	0.00245	0.3
TAU	t0 vs. t6	t0	t6	14	Mannheimia	0.02971	0.03613	0.03350	0.05500	0.77524	1
TAU	t0 vs. t6	t0	t6	14	Pasteurella	0.47344	0.52168	0.74189	0.54359	0.14584	1
TAU	t0 vs. t6	t0	t6	14	Rodentibacter	0.03732	0.11400	0.19019	0.27617	0.04852	1
TAU	t0 vs. t6	t0	t6	14	Acinetobacter	0.17023	0.26009	0.17818	0.25695	0.92531	1
TAU	t0 vs. t6	t0	t6	14	Enhydrobacter	0.00027	0.00102	0.00034	0.00091	0.85539	1
TAU	t0 vs. t6	t0	t6	14	Moraxella	0.35864	1.04798	0.35632	0.75620	0.99441	1
TAU	t0 vs. t6	t0	t6	14	Pseudomonas	0.00002	0.00008	0.00005	0.00019	0.61779	1
TAU	t0 vs. t6	t0	t6	14	Vibrio	0.00012	0.00045	0.00156	0.00583	0.37746	1
TAU	t0 vs. t6	t0	t6	14	Treponema	0.00444	0.00818	0.00954	0.02191	0.27249	1
TAU	t3 vs. t6	t3	t6	14	Actinomyces	2.80741	2.75012	3.53947	3.22755	0.37862	1
TAU	t3 vs. t6	t3	t6	14	Actinotignum	0.00361	0.01307	0.00010	0.00037	0.33399	1
TAU	t3 vs. t6	t3	t6	14	F0332	0.01660	0.06131	0.00086	0.00323	0.32916	1
TAU	t3 vs. t6	t3	t6	14	Alloscardovia	0.00282	0.00973	0.02313	0.04915	0.13271	1

TAU	t3 vs. t6	t3	t6	14	Bifidobacterium	0.00104	0.00390	0.00432	0.01617	0.33556	1
TAU	t3 vs. t6	t3	t6	14	Scardovia	0.00035	0.00100	0.00016	0.00046	0.54210	1
TAU	t3 vs. t6	t3	t6	14	Corynebacterium	0.01907	0.03379	0.02589	0.06460	0.58989	1
TAU	t3 vs. t6	t3	t6	14	Microbacterium	1.10069	1.74069	1.05506	1.49896	0.87010	1
TAU	t3 vs. t6	t3	t6	14	Paenarthrobacter	0.00274	0.00714	0.00067	0.00251	0.24135	1
TAU	t3 vs. t6	t3	t6	14	Rothia	3.61327	3.01531	5.61551	4.43608	0.06069	1
TAU	t3 vs. t6	t3	t6	14	Cutibacterium	0.00018	0.00050	0.00001	0.00005	0.19824	1
TAU	t3 vs. t6	t3	t6	14	Pseudopropionibacterium	0.00006	0.00021	0.00015	0.00056	0.58220	1
TAU	t3 vs. t6	t3	t6	14	Tessaracoccus	0.00006	0.00024	0.00056	0.00175	0.31999	1
TAU	t3 vs. t6	t3	t6	14	Atopobium	0.17757	0.27763	0.13377	0.15258	0.61739	1
TAU	t3 vs. t6	t3	t6	14	Cryptobacterium	0.00008	0.00029	0.00083	0.00310	0.33556	1
TAU	t3 vs. t6	t3	t6	14	Bacteroides	0.00576	0.01598	0.00042	0.00112	0.22558	1
TAU	t3 vs. t6	t3	t6	14	F0058	0.00116	0.00394	0.00509	0.01077	0.22111	1
TAU	t3 vs. t6	t3	t6	14	Porphyromonas	2.21920	2.16772	1.69109	1.32739	0.33047	1
TAU	t3 vs. t6	t3	t6	14	Alloprevotella	1.14683	1.26599	1.30732	1.35592	0.70845	1
TAU	t3 vs. t6	t3	t6	14	Prevotella	14.92275	12.62391	15.60541	12.67606	0.83035	1
TAU	t3 vs. t6	t3	t6	14	Rikenellaceae_RC9_gut_group	0.00358	0.00914	0.00017	0.00046	0.18205	1
TAU	t3 vs. t6	t3	t6	14	Tannerella	0.00349	0.00532	0.03019	0.05321	0.09179	1
TAU	t3 vs. t6	t3	t6	14	Capnocytophaga	0.28817	0.25938	0.45914	0.49981	0.19764	1
TAU	t3 vs. t6	t3	t6	14	Flavobacterium	0.00618	0.01724	0.00456	0.01089	0.77952	1
TAU	t3 vs. t6	t3	t6	14	Bergeyella	0.00043	0.00115	0.00145	0.00335	0.31197	1
TAU	t3 vs. t6	t3	t6	14	Chryseobacterium	0.00139	0.00278	0.00910	0.01703	0.12056	1
TAU	t3 vs. t6	t3	t6	14	Lentimicrobium	0.00142	0.00532	0.00019	0.00072	0.41187	1
TAU	t3 vs. t6	t3	t6	14	Campylobacter	0.78228	0.88443	1.27889	0.89383	0.11772	1
TAU	t3 vs. t6	t3	t6	14	Chloroplast	0.00159	0.00429	0.03439	0.08627	0.17814	1
TAU	t3 vs. t6	t3	t6	14	Solobacterium	0.22326	0.21172	0.26916	0.40133	0.67250	1
TAU	t3 vs. t6	t3	t6	14	Abiotrophia	0.00347	0.00886	0.00464	0.01735	0.83378	1
TAU	t3 vs. t6	t3	t6	14	Globicatella	0.01169	0.03713	0.00350	0.01012	0.45194	1
TAU	t3 vs. t6	t3	t6	14	Alkalibacterium	0.00068	0.00186	0.00005	0.00019	0.23423	1
TAU	t3 vs. t6	t3	t6	14	Carnobacterium	0.05201	0.08358	0.01859	0.04446	0.08376	1
TAU	t3 vs. t6	t3	t6	14	Granulicatella	3.51471	2.70597	2.79156	1.80983	0.23870	1
TAU	t3 vs. t6	t3	t6	14	Enterococcus	0.68720	0.50289	0.48815	0.40923	0.15305	1
TAU	t3 vs. t6	t3	t6	14	Lactobacillus	0.43607	0.30240	0.49028	0.71654	0.80947	1
TAU	t3 vs. t6	t3	t6	14	Streptococcus	24.76592	15.92163	18.70532	5.72178	0.13702	1
TAU	t3 vs. t6	t3	t6	14	Mycoplasma	0.00095	0.00225	0.00036	0.00077	0.31830	1
TAU	t3 vs. t6	t3	t6	14	RF39	0.00237	0.00610	0.00374	0.01201	0.49345	1
TAU	t3 vs. t6	t3	t6	14	Gemella	2.30703	1.77406	2.12580	1.19554	0.72061	1
TAU	t3 vs. t6	t3	t6	14	Staphylococcus	0.00171	0.00574	0.00007	0.00027	0.28177	1
TAU	t3 vs. t6	t3	t6	14	Clostridia_UCG-014	0.11681	0.09524	0.39116	0.55084	0.08491	1
TAU	t3 vs. t6	t3	t6	14	Defluviitaleaceae_UCG-011	0.00456	0.01182	0.00658	0.02427	0.75720	1

TAU	t3 vs. t6	t3	t6	14	Butyrivibrio	0.09097	0.27495	0.10309	0.26113	0.90421	1
TAU	t3 vs. t6	t3	t6	14	Catonella	0.06454	0.09309	0.05061	0.08685	0.57494	1
TAU	t3 vs. t6	t3	t6	14	Howardella	0.00315	0.01179	0.00276	0.00951	0.55577	1
TAU	t3 vs. t6	t3	t6	14	Johnsonella	0.00279	0.00627	0.02020	0.07316	0.37332	1
TAU	t3 vs. t6	t3	t6	14	Lachnoanaerobaculum	0.37839	0.45944	0.24856	0.20446	0.12964	1
TAU	t3 vs. t6	t3	t6	14	Lachnoclostridium	0.36274	0.36297	0.32611	0.22958	0.53651	1
TAU	t3 vs. t6	t3	t6	14	Oribacterium	0.30240	0.35611	0.21577	0.15645	0.32956	1
TAU	t3 vs. t6	t3	t6	14	Shuttleworthia	0.00832	0.02820	0.00134	0.00412	0.29859	1
TAU	t3 vs. t6	t3	t6	14	Stomatobaculum	0.12709	0.22801	0.05622	0.05987	0.26814	1
TAU	t3 vs. t6	t3	t6	14	[Eubacterium]_brachy_group	0.00127	0.00414	0.00026	0.00068	0.34068	1
TAU	t3 vs. t6	t3	t6	14	[Eubacterium]_nodatum_group	0.12518	0.19149	0.13159	0.18797	0.91446	1
TAU	t3 vs. t6	t3	t6	14	Amnipila	0.00525	0.01964	0.00350	0.01248	0.38000	1
TAU	t3 vs. t6	t3	t6	14	Family_XIII_UCG-001	0.01460	0.04144	0.00146	0.00548	0.24361	1
TAU	t3 vs. t6	t3	t6	14	Mogibacterium	0.05936	0.08283	0.04076	0.04781	0.28951	1
TAU	t3 vs. t6	t3	t6	14	[Eubacterium]_yurii_group	0.02273	0.04368	0.01426	0.02546	0.52461	1
TAU	t3 vs. t6	t3	t6	14	Filifactor	0.00620	0.01679	0.00355	0.01277	0.65729	1
TAU	t3 vs. t6	t3	t6	14	Peptostreptococcus	0.43835	0.58800	0.16984	0.15790	0.09719	1
TAU	t3 vs. t6	t3	t6	14	Parvimonas	0.01171	0.01628	0.00683	0.01295	0.14188	1
TAU	t3 vs. t6	t3	t6	14	Selenomonas	0.13794	0.23685	0.26716	0.55617	0.26126	1
TAU	t3 vs. t6	t3	t6	14	Dialister	0.27331	0.95139	0.05711	0.12504	0.41749	1
TAU	t3 vs. t6	t3	t6	14	Megasphaera	0.36566	1.08207	0.28249	0.39747	0.71654	1
TAU	t3 vs. t6	t3	t6	14	Veillonella	6.36350	3.59567	6.08570	4.07698	0.82335	1
TAU	t3 vs. t6	t3	t6	14	Fusobacterium	2.64520	2.05664	2.56707	1.76084	0.92332	1
TAU	t3 vs. t6	t3	t6	14	Leptotrichia	0.46102	0.39471	1.18114	2.08977	0.24350	1
TAU	t3 vs. t6	t3	t6	14	Streptobacillus	0.00006	0.00024	0.00004	0.00016	0.79291	1
TAU	t3 vs. t6	t3	t6	14	Absconditabacteriales_(SR1)	0.09146	0.13815	0.12134	0.30848	0.73252	1
TAU	t3 vs. t6	t3	t6	14	Gracilibacteria	0.00024	0.00088	0.00049	0.00099	0.37621	1
TAU	t3 vs. t6	t3	t6	14	JGI_0000069-P22	0.00056	0.00137	0.00059	0.00176	0.93008	1
TAU	t3 vs. t6	t3	t6	14	Candidatus_Saccharimonas	0.07411	0.07710	0.14139	0.31809	0.37842	1
TAU	t3 vs. t6	t3	t6	14	Saccharimonadaceae	0.02099	0.02390	0.03079	0.07408	0.56400	1
TAU	t3 vs. t6	t3	t6	14	TM7a	0.00079	0.00198	0.00031	0.00082	0.43556	1
TAU	t3 vs. t6	t3	t6	14	TM7x	0.22889	0.30590	0.51556	0.72738	0.18441	1
TAU	t3 vs. t6	t3	t6	14	Saccharimonadales	0.07271	0.10514	0.22759	0.44821	0.13594	1
TAU	t3 vs. t6	t3	t6	14	Sphingomonas	0.00002	0.00008	0.00009	0.00035	0.47409	1
TAU	t3 vs. t6	t3	t6	14	Shewanella	0.00553	0.00905	0.00084	0.00174	0.08206	1
TAU	t3 vs. t6	t3	t6	14	Lautropia	0.12198	0.16071	0.23243	0.52903	0.45562	1
TAU	t3 vs. t6	t3	t6	14	Acidovorax	0.00108	0.00269	0.00532	0.01707	0.31327	1
TAU	t3 vs. t6	t3	t6	14	Brachymonas	0.00111	0.00181	0.00426	0.01471	0.42968	1
TAU	t3 vs. t6	t3	t6	14	Thiobacillus	0.01245	0.02725	0.00079	0.00225	0.11545	1

TAU	t3 vs. t6	t3	t6	14	Alysiella	0.00203	0.00701	0.00137	0.00456	0.33556	1
TAU	t3 vs. t6	t3	t6	14	Eikenella	0.00067	0.00134	0.00026	0.00096	0.39404	1
TAU	t3 vs. t6	t3	t6	14	Kingella	0.01114	0.03723	0.00301	0.01091	0.45744	1
TAU	t3 vs. t6	t3	t6	14	Neisseria	15.97453	9.65344	18.43358	10.45391	0.31817	1
TAU	t3 vs. t6	t3	t6	14	Simonsiella	0.00059	0.00149	0.00555	0.01671	0.25285	1
TAU	t3 vs. t6	t3	t6	14	Cardiobacterium	0.01058	0.01388	0.01455	0.02053	0.58543	1
TAU	t3 vs. t6	t3	t6	14	Escherichia-Shigella	0.00096	0.00350	0.00007	0.00027	0.36368	1
TAU	t3 vs. t6	t3	t6	14	Serratia	0.61586	0.72665	0.45617	0.45175	0.36221	1
TAU	t3 vs. t6	t3	t6	14	Actinobacillus	0.08434	0.11592	0.10369	0.10321	0.66320	1
TAU	t3 vs. t6	t3	t6	14	Aggregatibacter	0.14104	0.20354	0.14927	0.32808	0.93430	1
TAU	t3 vs. t6	t3	t6	14	Haemophilus	4.08779	2.27604	4.93179	2.09861	0.17203	1
TAU	t3 vs. t6	t3	t6	14	Mannheimia	0.05224	0.09314	0.03350	0.05500	0.39817	1
TAU	t3 vs. t6	t3	t6	14	Pasteurella	0.85935	0.75227	0.74189	0.54359	0.65480	1
TAU	t3 vs. t6	t3	t6	14	Rodentibacter	0.15400	0.29892	0.19019	0.27617	0.66201	1
TAU	t3 vs. t6	t3	t6	14	Acinetobacter	0.07994	0.18686	0.17818	0.25695	0.14114	1
TAU	t3 vs. t6	t3	t6	14	Moraxella	0.51193	1.18720	0.35632	0.75620	0.65036	1
TAU	t3 vs. t6	t3	t6	14	Pseudomonas	0.00006	0.00015	0.00005	0.00019	0.91633	1
TAU	t3 vs. t6	t3	t6	14	Vibrio	0.00009	0.00035	0.00156	0.00583	0.36711	1
TAU	t3 vs. t6	t3	t6	14	Treponema	0.00637	0.01305	0.00954	0.02191	0.56315	1

Abbreviations: OPP, Intensive oral prophylaxis program; TAU, Treatmen-As-Usual

Supplementary Table S5: Change in abundance of species during the periods of intervention in the IP and TAU groups

treatment group	comparison	group1	group2	N_pairs	genus	species	group1_mean	group1_sd	group2_mean	group2_sd	p_value	FDR
OPP	t0 vs. t3	t0	t3	11	Actinomyces	Actinomyces_graevenitzii	0.4527	0.8580	0.6114	1.2446	0.7533	1
OPP	t0 vs. t3	t0	t3	11	Actinomyces	Actinomyces_naeslundii	0.0579	0.1314	0.0097	0.0087	0.2385	1
OPP	t0 vs. t3	t0	t3	11	Actinomyces	Schaalia_odontolytica	0.1400	0.1475	0.2322	0.4610	0.4034	1
OPP	t0 vs. t3	t0	t3	11	Alloscardovia	Alloscardovia_omnicolens	0.0000	0.0002	0.0102	0.0238	0.1868	1
OPP	t0 vs. t3	t0	t3	11	Corynebacterium	Corynebacterium_diphtheriae	0.0003	0.0009	0.0003	0.0006	1.0000	1
OPP	t0 vs. t3	t0	t3	11	Corynebacterium	Corynebacterium_durum	0.0303	0.0603	0.0017	0.0032	0.1480	1
OPP	t0 vs. t3	t0	t3	11	Corynebacterium	Corynebacterium_matruchotii	0.0012	0.0033	0.0012	0.0026	0.9890	1
OPP	t0 vs. t3	t0	t3	11	Rothia	Rothia_aeria	0.1138	0.0890	0.0931	0.1494	0.7252	1
OPP	t0 vs. t3	t0	t3	11	Rothia	Rothia_mucilaginosa	7.4894	11.2522	6.5748	13.1869	0.8607	1
OPP	t0 vs. t3	t0	t3	11	Atopobium	Lancefieldella_parvula	0.0078	0.0167	0.0181	0.0504	0.5507	1
OPP	t0 vs. t3	t0	t3	11	Bacteroides	Bacteroidaceae_bacterium	0.0215	0.0471	0.0005	0.0013	0.1721	1
OPP	t0 vs. t3	t0	t3	11	F0058	Bacteroidetes_oral	0.0002	0.0005	0.0009	0.0028	0.2867	1
OPP	t0 vs. t3	t0	t3	11	Porphyromonas	Porphyromonas_catoniae	0.0118	0.0151	0.0092	0.0230	0.6346	1
OPP	t0 vs. t3	t0	t3	11	Porphyromonas	Porphyromonas_endodontalis	0.0049	0.0106	0.0045	0.0103	0.7970	1
OPP	t0 vs. t3	t0	t3	11	Porphyromonas	Porphyromonas_gingivalis	0.0002	0.0005	0.0019	0.0052	0.2496	1
OPP	t0 vs. t3	t0	t3	11	Porphyromonas	Porphyromonas_pasteri	0.8892	0.9695	2.3563	2.4802	0.0760	1
OPP	t0 vs. t3	t0	t3	11	Alloprevotella	Alloprevotella_rava	0.1162	0.3855	0.5438	1.5106	0.3972	1
OPP	t0 vs. t3	t0	t3	11	Alloprevotella	Alloprevotella_tannerae	0.0163	0.0266	0.0757	0.1550	0.2039	1
OPP	t0 vs. t3	t0	t3	11	Alloprevotella	Prevotellaceae_bacterium	0.4837	0.7709	1.2266	1.6572	0.0582	1
OPP	t0 vs. t3	t0	t3	11	Prevotella	Prevotella_buccae	0.0001	0.0002	0.0001	0.0005	0.3409	1
OPP	t0 vs. t3	t0	t3	11	Prevotella	Prevotella_denticola	0.0001	0.0003	0.0004	0.0008	0.1678	1
OPP	t0 vs. t3	t0	t3	11	Prevotella	Prevotella_fusca	0.0042	0.0140	0.0057	0.0169	0.8376	1
OPP	t0 vs. t3	t0	t3	11	Prevotella	Prevotella_histicola	0.1543	0.3372	0.0690	0.1786	0.1482	1
OPP	t0 vs. t3	t0	t3	11	Prevotella	Prevotella_intermedia	0.0741	0.1412	0.1040	0.2137	0.6309	1
OPP	t0 vs. t3	t0	t3	11	Prevotella	Prevotella_jejuni	0.0356	0.1117	0.0718	0.2231	0.3095	1
OPP	t0 vs. t3	t0	t3	11	Prevotella	Prevotella_loescheii	0.1358	0.2291	0.1466	0.2004	0.5961	1
OPP	t0 vs. t3	t0	t3	11	Prevotella	Prevotella_melaninogenica	7.1396	8.4565	6.0671	4.9567	0.5515	1
OPP	t0 vs. t3	t0	t3	11	Prevotella	Prevotella_nanceiensis	0.5448	0.5928	0.4528	0.6055	0.7051	1
OPP	t0 vs. t3	t0	t3	11	Prevotella	Prevotella_nigrescens	0.0067	0.0191	0.0328	0.0771	0.3053	1
OPP	t0 vs. t3	t0	t3	11	Prevotella	Prevotella_oulorum	0.0223	0.0539	0.0315	0.0481	0.5968	1
OPP	t0 vs. t3	t0	t3	11	Prevotella	Prevotella_pallens	0.1507	0.1887	0.6107	0.8936	0.0719	1
OPP	t0 vs. t3	t0	t3	11	Prevotella	Prevotella_salivae	0.3452	0.6514	0.5509	0.6651	0.1500	1
OPP	t0 vs. t3	t0	t3	11	Prevotella	Prevotella_shahii	0.0448	0.0926	0.0720	0.1169	0.0420	1
OPP	t0 vs. t3	t0	t3	11	Prevotella	Prevotella_veroralis	0.0641	0.2127	0.1344	0.4398	0.3293	1
OPP	t0 vs. t3	t0	t3	11	Tannerella	Tannerella_forsythia	0.0038	0.0071	0.0046	0.0128	0.7507	1
OPP	t0 vs. t3	t0	t3	11	Capnocytophaga	Capnocytophaga_gingivalis	0.0407	0.0663	0.0606	0.0580	0.1382	1
OPP	t0 vs. t3	t0	t3	11	Capnocytophaga	Capnocytophaga_granulosa	0.0033	0.0066	0.0060	0.0115	0.3818	1
OPP	t0 vs. t3	t0	t3	11	Capnocytophaga	Capnocytophaga_leadbetteri	0.0316	0.0386	0.0489	0.0434	0.3672	1
OPP	t0 vs. t3	t0	t3	11	Capnocytophaga	Capnocytophaga_ochracea	0.0298	0.0465	0.0320	0.0319	0.8856	1
OPP	t0 vs. t3	t0	t3	11	Capnocytophaga	Capnocytophaga_sputigena	0.1802	0.2986	0.1172	0.1883	0.2260	1
OPP	t0 vs. t3	t0	t3	11	Bergeyella	Flavobacteriaceae_bacterium	0.0000	0.0001	0.0001	0.0003	0.5286	1
OPP	t0 vs. t3	t0	t3	11	Chryseobacterium	Chryseobacterium_reticulitermitis	0.0005	0.0012	0.0001	0.0005	0.4027	1

OPP	t0 vs. t3	t0	t3	11	Campylobacter	Campylobacter_concisus	0.0240	0.0266	0.0543	0.0781	0.2664	1
OPP	t0 vs. t3	t0	t3	11	Campylobacter	Campylobacter_showae	0.0006	0.0009	0.0021	0.0043	0.2158	1
OPP	t0 vs. t3	t0	t3	11	Solobacterium	Solobacterium_moorei	0.1255	0.1258	0.1100	0.1032	0.5514	1
OPP	t0 vs. t3	t0	t3	11	Globicatella	Aerococcaceae_bacterium	0.0012	0.0031	0.0031	0.0082	0.2467	1
OPP	t0 vs. t3	t0	t3	11	Granulicatella	Granulicatella_adiacens	0.0150	0.0186	0.0017	0.0043	0.0566	1
OPP	t0 vs. t3	t0	t3	11	Granulicatella	Granulicatella_elegans	0.0141	0.0207	0.0219	0.0223	0.2628	1
OPP	t0 vs. t3	t0	t3	11	Lactobacillus	Lactobacillus_sakei	0.0027	0.0090	0.0049	0.0164	0.7107	1
OPP	t0 vs. t3	t0	t3	11	Lactococcus	Lactococcus_lactis	0.0019	0.0054	0.0015	0.0049	0.8531	1
OPP	t0 vs. t3	t0	t3	11	Streptococcus	Streptococcus_anginosus	0.0042	0.0101	0.0057	0.0140	0.2469	1
OPP	t0 vs. t3	t0	t3	11	Streptococcus	Streptococcus_australis	0.0108	0.0209	0.0057	0.0085	0.3325	1
OPP	t0 vs. t3	t0	t3	11	Streptococcus	Streptococcus_cristatus	0.0364	0.0773	0.0238	0.0379	0.4581	1
OPP	t0 vs. t3	t0	t3	11	Streptococcus	Streptococcus_gordonii	0.0300	0.0329	0.0180	0.0225	0.2797	1
OPP	t0 vs. t3	t0	t3	11	Streptococcus	Streptococcus_infantis	0.0302	0.0377	0.0331	0.0387	0.8618	1
OPP	t0 vs. t3	t0	t3	11	Streptococcus	Streptococcus_mitis	0.0028	0.0048	0.0121	0.0190	0.1268	1
OPP	t0 vs. t3	t0	t3	11	Streptococcus	Streptococcus_oralis	0.0085	0.0159	0.0053	0.0121	0.3626	1
OPP	t0 vs. t3	t0	t3	11	Streptococcus	Streptococcus_parasanguinis	1.2518	0.6983	1.6872	2.1285	0.5167	1
OPP	t0 vs. t3	t0	t3	11	Streptococcus	Streptococcus_peroris	0.0022	0.0072	0.0007	0.0024	0.3409	1
OPP	t0 vs. t3	t0	t3	11	Streptococcus	Streptococcus_pneumoniae	0.2735	0.3073	0.2529	0.2820	0.8641	1
OPP	t0 vs. t3	t0	t3	11	Streptococcus	Streptococcus_salivarius	12.9143	12.9177	5.7875	7.8308	0.0341	1
OPP	t0 vs. t3	t0	t3	11	Streptococcus	Streptococcus_sanguinis	0.1223	0.2138	0.2304	0.5170	0.3963	1
OPP	t0 vs. t3	t0	t3	11	Streptococcus	Streptococcus_suis	0.2277	0.2462	0.2196	0.1967	0.8920	1
OPP	t0 vs. t3	t0	t3	11	RF39	Firmicutes_oral	0.0020	0.0065	0.0063	0.0209	0.5381	1
OPP	t0 vs. t3	t0	t3	11	Gemella	Gemella_haemolysans	1.1679	1.0091	1.3291	0.9068	0.5482	1
OPP	t0 vs. t3	t0	t3	11	Gemella	Gemella_morbillorum	0.0146	0.0280	0.0138	0.0267	0.9092	1
OPP	t0 vs. t3	t0	t3	11	Gemella	Gemella_sanguinis	0.0618	0.0926	0.0934	0.0746	0.2543	1
OPP	t0 vs. t3	t0	t3	11	Clostridia_UCG-014	Clostridiales_bacterium	0.2497	0.3139	0.4195	0.7612	0.4325	1
OPP	t0 vs. t3	t0	t3	11	Catonella	Catonella_morbi	0.0319	0.0300	0.0273	0.0267	0.6421	1
OPP	t0 vs. t3	t0	t3	11	Johnsonella	Lachnospiraceae_bacterium	0.0053	0.0132	0.0056	0.0128	0.9567	1
OPP	t0 vs. t3	t0	t3	11	Lachnoanaerobaculum	Lachnoanaerobaculum_umeaense	0.1867	0.1901	0.0920	0.0524	0.0717	1
OPP	t0 vs. t3	t0	t3	11	Stomatobaculum	Stomatobaculum_longum	0.0619	0.0948	0.0386	0.0442	0.4968	1
OPP	t0 vs. t3	t0	t3	11	[Eubacterium]_brachy_group	[Eubacterium]_brachy	0.0002	0.0006	0.0010	0.0032	0.3409	1
OPP	t0 vs. t3	t0	t3	11	[Eubacterium]_nodatum_group	Eubacterium_sulci	0.0017	0.0057	0.0122	0.0327	0.3303	1
OPP	t0 vs. t3	t0	t3	11	[Eubacterium]_yurii_group	[Eubacterium]_yurii	0.0167	0.0364	0.0070	0.0120	0.4213	1
OPP	t0 vs. t3	t0	t3	11	Parvimonas	Parvimonas_micra	0.0012	0.0020	0.0003	0.0005	0.1812	1
OPP	t0 vs. t3	t0	t3	11	Selenomonas	Selenomonas_flueggei	0.0004	0.0015	0.0018	0.0052	0.4374	1
OPP	t0 vs. t3	t0	t3	11	Selenomonas	Selenomonas_massiliensis	0.0007	0.0023	0.0052	0.0147	0.3467	1
OPP	t0 vs. t3	t0	t3	11	Selenomonas	Selenomonas_noxia	0.0037	0.0122	0.0194	0.0364	0.2181	1
OPP	t0 vs. t3	t0	t3	11	Selenomonas	Selenomonas_sputigena	0.0050	0.0127	0.0242	0.0575	0.1999	1
OPP	t0 vs. t3	t0	t3	11	Dialister	Dialister_invisus	0.0012	0.0033	0.0013	0.0041	0.9149	1
OPP	t0 vs. t3	t0	t3	11	Dialister	Dialister_pneumosintes	0.0005	0.0018	0.0036	0.0119	0.3409	1
OPP	t0 vs. t3	t0	t3	11	Megasphaera	Megasphaera_elsdenii	0.0002	0.0005	0.0001	0.0005	0.9346	1
OPP	t0 vs. t3	t0	t3	11	Megasphaera	Megasphaera_micronuciformis	0.1784	0.4156	0.1193	0.1456	0.5481	1
OPP	t0 vs. t3	t0	t3	11	Veillonella	Veillonella_atypica	1.3645	2.0629	1.1405	1.5780	0.5022	1
OPP	t0 vs. t3	t0	t3	11	Veillonella	Veillonella_parvula	2.7639	3.4432	2.3777	1.4028	0.6146	1
OPP	t0 vs. t3	t0	t3	11	Veillonella	Veillonella_rogosae	0.1076	0.2355	0.0036	0.0119	0.1768	1
OPP	t0 vs. t3	t0	t3	11	Fusobacterium	Fusobacterium_nucleatum	0.4262	0.3941	0.7319	0.7146	0.2215	1
OPP	t0 vs. t3	t0	t3	11	Fusobacterium	Fusobacterium_periodonticum	1.3063	1.1876	1.7814	1.3016	0.2265	1
OPP	t0 vs. t3	t0	t3	11	Leptotrichia	Leptotrichia_buccalis	0.2074	0.3306	0.1946	0.4653	0.9278	1
OPP	t0 vs. t3	t0	t3	11	Leptotrichia	Leptotrichia_goodfellowii	0.0000	0.0001	0.0350	0.1159	0.3412	1
OPP	t0 vs. t3	t0	t3	11	Leptotrichia	Leptotrichia_hofstadii	0.0042	0.0104	0.0011	0.0023	0.3667	1

OPP	t0 vs. t3	t0	t3	11	Leptotrichia	Leptotrichia_hongkongensis	0.0002	0.0004	0.0010	0.0013	0.0713	1
OPP	t0 vs. t3	t0	t3	11	Leptotrichia	Leptotrichia_shahii	0.0057	0.0127	0.0053	0.0118	0.9275	1
OPP	t0 vs. t3	t0	t3	11	Leptotrichia	Leptotrichia_trevisanii	0.0007	0.0024	0.0002	0.0007	0.5172	1
OPP	t0 vs. t3	t0	t3	11	Leptotrichia	Leptotrichia_wadei	0.1876	0.3189	0.1906	0.3697	0.9825	1
OPP	t0 vs. t3	t0	t3	11	Absconditabacteriales_(SR1)	SR1_bacterium	0.0138	0.0278	0.0087	0.0149	0.3766	1
OPP	t0 vs. t3	t0	t3	11	Gracilibacteria	Gracilibacteria_bacterium	0.0005	0.0011	0.0008	0.0020	0.5814	1
OPP	t0 vs. t3	t0	t3	11	Candidatus_Saccharimonas	TM7_phylum	0.0161	0.0255	0.0633	0.0799	0.0524	1
OPP	t0 vs. t3	t0	t3	11	Saccharimonadaceae	TM7_bacterium	0.0084	0.0246	0.0069	0.0196	0.4147	1
OPP	t0 vs. t3	t0	t3	11	Saccharimonadaceae	TM7_phylum	0.0049	0.0161	0.0030	0.0099	0.3409	1
OPP	t0 vs. t3	t0	t3	11	TM7a	candidate_division	0.0075	0.0245	0.0091	0.0287	0.2293	1
OPP	t0 vs. t3	t0	t3	11	TM7a	TM7_phylum	0.0024	0.0079	0.0005	0.0011	0.4095	1
OPP	t0 vs. t3	t0	t3	11	TM7x	Candidatus_Saccharibacteria	0.0275	0.0505	0.1134	0.1493	0.0286	1
OPP	t0 vs. t3	t0	t3	11	TM7x	TM7_phylum	0.0514	0.0560	0.1628	0.2439	0.1777	1
OPP	t0 vs. t3	t0	t3	11	Saccharimonadales	TM7_phylum	0.1025	0.2362	0.1042	0.1636	0.9821	1
OPP	t0 vs. t3	t0	t3	11	Shewanella	Shewanella_putrefaciens	0.0030	0.0048	0.0009	0.0013	0.2175	1
OPP	t0 vs. t3	t0	t3	11	Lautropia	Lautropia_mirabilis	0.0471	0.0935	0.0431	0.0764	0.8637	1
OPP	t0 vs. t3	t0	t3	11	Brachymonas	Brachymonas_denitrificans	0.0121	0.0350	0.0015	0.0030	0.3432	1
OPP	t0 vs. t3	t0	t3	11	Eikenella	Eikenella_corrodens	0.0003	0.0010	0.0004	0.0013	0.8523	1
OPP	t0 vs. t3	t0	t3	11	Kingella	Kingella_oralis	0.0022	0.0072	0.0003	0.0009	0.4118	1
OPP	t0 vs. t3	t0	t3	11	Neisseria	Neisseria_bacilliformis	0.0018	0.0045	0.0017	0.0058	0.9928	1
OPP	t0 vs. t3	t0	t3	11	Neisseria	Neisseria_elongata	0.0216	0.0218	0.0348	0.0885	0.6026	1
OPP	t0 vs. t3	t0	t3	11	Neisseria	Neisseria_meningitidis	7.5649	6.9417	11.6960	9.5807	0.2446	1
OPP	t0 vs. t3	t0	t3	11	Neisseria	Neisseria_oralis	0.0003	0.0008	0.0065	0.0192	0.3096	1
OPP	t0 vs. t3	t0	t3	11	Neisseria	Neisseria_perflava	0.0014	0.0036	0.0058	0.0081	0.0584	1
OPP	t0 vs. t3	t0	t3	11	Simonsiella	Simonsiella_muelleri	0.0026	0.0057	0.0029	0.0064	0.9135	1
OPP	t0 vs. t3	t0	t3	11	Snodgrassella	Snodgrassella_alvi	0.0006	0.0019	0.0003	0.0011	0.3409	1
OPP	t0 vs. t3	t0	t3	11	Cardiobacterium	Cardiobacterium_hominis	0.0012	0.0016	0.0042	0.0069	0.1704	1
OPP	t0 vs. t3	t0	t3	11	Cardiobacterium	Cardiobacterium_valvarum	0.0019	0.0031	0.0027	0.0044	0.6406	1
OPP	t0 vs. t3	t0	t3	11	Actinobacillus	Actinobacillus_pleuropneumoniae	0.0122	0.0359	0.0268	0.0434	0.4375	1
OPP	t0 vs. t3	t0	t3	11	Actinobacillus	Haemophilus_parahaemolyticus	0.0152	0.0252	0.0141	0.0383	0.9418	1
OPP	t0 vs. t3	t0	t3	11	Aggregatibacter	Aggregatibacter_aphrophilus	0.0323	0.0521	0.0579	0.1020	0.5110	1
OPP	t0 vs. t3	t0	t3	11	Aggregatibacter	Aggregatibacter_segnis	0.0111	0.0150	0.0324	0.0605	0.2552	1
OPP	t0 vs. t3	t0	t3	11	Haemophilus	[Haemophilus]_ducreyi	0.0019	0.0043	0.0023	0.0035	0.8474	1
OPP	t0 vs. t3	t0	t3	11	Haemophilus	Haemophilus_haemolyticus	0.0377	0.0588	0.0192	0.0212	0.3744	1
OPP	t0 vs. t3	t0	t3	11	Haemophilus	Haemophilus_influenzae	0.0300	0.0444	0.0561	0.0784	0.3959	1
OPP	t0 vs. t3	t0	t3	11	Haemophilus	Haemophilus_parainfluenzae	1.7015	1.5369	1.6409	1.0589	0.9178	1
OPP	t0 vs. t3	t0	t3	11	Haemophilus	Haemophilus_pittmaniae	0.8861	0.7601	0.8939	0.7948	0.9822	1
OPP	t0 vs. t3	t0	t3	11	Haemophilus	Haemophilus_sputorum	0.0269	0.0481	0.0217	0.0434	0.7209	1
OPP	t0 vs. t3	t0	t3	11	Mannheimia	Mannheimia_haemolytica	0.0007	0.0022	0.0091	0.0137	0.0695	1
OPP	t0 vs. t3	t0	t3	11	Pasteurella	Pasteurella_multocida	0.6332	0.7165	0.7370	0.6648	0.7721	1
OPP	t0 vs. t3	t0	t3	11	Rodentibacter	Pasteurellaceae_bacterium	0.0012	0.0040	0.0175	0.0537	0.3442	1
OPP	t0 vs. t3	t0	t3	11	Pseudomonas	Pseudomonas_fluorescens	0.0001	0.0003	0.0001	0.0003	0.3409	1
OPP	t0 vs. t3	t0	t3	11	Treponema	Treponema_medium	0.0026	0.0059	0.0027	0.0055	0.9521	1
OPP	t0 vs. t3	t0	t3	11	Treponema	Treponema_socranskii	0.0000	0.0001	0.0000	0.0001	0.3409	1
OPP	t0 vs. t6	t0	t6	13	Actinomyces	Actinomyces_graevenitzii	0.4446	0.8003	0.7508	1.1802	0.4756	1
OPP	t0 vs. t6	t0	t6	13	Actinomyces	Actinomyces_lingnae	0.0011	0.0031	0.0001	0.0002	0.2484	1
OPP	t0 vs. t6	t0	t6	13	Actinomyces	Actinomyces_naeslundii	0.0490	0.1219	0.0511	0.0865	0.9409	1
OPP	t0 vs. t6	t0	t6	13	Actinomyces	Schaalia_odontolytica	0.1374	0.1438	0.2347	0.3015	0.2971	1
OPP	t0 vs. t6	t0	t6	13	Alloscardovia	Alloscardovia_omnicolens	0.0000	0.0001	0.0798	0.2834	0.3303	1
OPP	t0 vs. t6	t0	t6	13	Bifidobacterium	Bifidobacterium_longum	0.0001	0.0005	0.0069	0.0172	0.1710	1

OPP	t0 vs. t6	t0	t6	13	Corynebacterium	Corynebacterium_diphtheriae	0.0025	0.0081	0.0016	0.0034	0.7173	1
OPP	t0 vs. t6	t0	t6	13	Corynebacterium	Corynebacterium_durum	0.0256	0.0562	0.0039	0.0063	0.1785	1
OPP	t0 vs. t6	t0	t6	13	Corynebacterium	Corynebacterium_matruchotii	0.0010	0.0030	0.0074	0.0155	0.1271	1
OPP	t0 vs. t6	t0	t6	13	Rothia	Rothia_aeria	0.0998	0.0886	0.0777	0.1272	0.6021	1
OPP	t0 vs. t6	t0	t6	13	Rothia	Rothia_mucilaginosa	7.2558	10.5437	5.3643	5.5513	0.4860	1
OPP	t0 vs. t6	t0	t6	13	Atopobium	Lancefieldella_parvula	0.0092	0.0172	0.0171	0.0230	0.2488	1
OPP	t0 vs. t6	t0	t6	13	Bacteroides	Bacteroidaceae_bacterium	0.0202	0.0435	0.0008	0.0020	0.1352	1
OPP	t0 vs. t6	t0	t6	13	F0058	Bacteroidetes_oral	0.0001	0.0005	0.0013	0.0027	0.1235	1
OPP	t0 vs. t6	t0	t6	13	Porphyromonas	Porphyromonas_catoniae	0.0100	0.0145	0.0141	0.0209	0.4299	1
OPP	t0 vs. t6	t0	t6	13	Porphyromonas	Porphyromonas_endodontalis	0.0164	0.0443	0.0092	0.0214	0.5944	1
OPP	t0 vs. t6	t0	t6	13	Porphyromonas	Porphyromonas_gingivalis	0.0001	0.0005	0.0008	0.0019	0.1857	1
OPP	t0 vs. t6	t0	t6	13	Porphyromonas	Porphyromonas_pasteri	0.7549	0.9438	1.1340	1.0675	0.2978	1
OPP	t0 vs. t6	t0	t6	13	Alloprevotella	Alloprevotella_rava	0.1697	0.4203	0.1116	0.3853	0.7324	1
OPP	t0 vs. t6	t0	t6	13	Alloprevotella	Alloprevotella_tannerae	0.0138	0.0250	0.0673	0.1705	0.2672	1
OPP	t0 vs. t6	t0	t6	13	Alloprevotella	Prevotellaceae_bacterium	0.4119	0.7253	0.6303	0.7166	0.2255	1
OPP	t0 vs. t6	t0	t6	13	Prevotella	Massiliprevotella_massiliensis	0.0002	0.0006	0.0001	0.0004	0.6294	1
OPP	t0 vs. t6	t0	t6	13	Prevotella	Prevotella_aurantiaca	0.0266	0.0959	0.0349	0.1257	0.3370	1
OPP	t0 vs. t6	t0	t6	13	Prevotella	Prevotella_buccae	0.0001	0.0002	0.0001	0.0003	0.3370	1
OPP	t0 vs. t6	t0	t6	13	Prevotella	Prevotella_denticola	0.0001	0.0002	0.0065	0.0161	0.1771	1
OPP	t0 vs. t6	t0	t6	13	Prevotella	Prevotella_fusca	0.0037	0.0128	0.0130	0.0254	0.2296	1
OPP	t0 vs. t6	t0	t6	13	Prevotella	Prevotella_histicola	0.2387	0.4684	0.0458	0.0921	0.1600	1
OPP	t0 vs. t6	t0	t6	13	Prevotella	Prevotella_intermedia	0.0627	0.1319	0.1365	0.1983	0.1662	1
OPP	t0 vs. t6	t0	t6	13	Prevotella	Prevotella_jejuni	0.0349	0.1028	0.0815	0.1584	0.3090	1
OPP	t0 vs. t6	t0	t6	13	Prevotella	Prevotella_loescheii	0.1212	0.2129	0.3297	0.8755	0.3273	1
OPP	t0 vs. t6	t0	t6	13	Prevotella	Prevotella_melaninogenica	7.7005	7.8477	10.6371	10.1883	0.2730	1
OPP	t0 vs. t6	t0	t6	13	Prevotella	Prevotella_nanceiensis	0.4636	0.5764	0.3465	0.4289	0.1983	1
OPP	t0 vs. t6	t0	t6	13	Prevotella	Prevotella_nigrescens	0.0064	0.0175	0.0269	0.0495	0.2034	1
OPP	t0 vs. t6	t0	t6	13	Prevotella	Prevotella_oulorum	0.0222	0.0500	0.0449	0.1000	0.5004	1
OPP	t0 vs. t6	t0	t6	13	Prevotella	Prevotella_pallens	0.1920	0.2628	0.5867	1.1582	0.2127	1
OPP	t0 vs. t6	t0	t6	13	Prevotella	Prevotella_pleuritidis	0.0000	0.0002	0.0010	0.0037	0.3603	1
OPP	t0 vs. t6	t0	t6	13	Prevotella	Prevotella_salivae	0.3297	0.6041	0.3954	0.4450	0.7338	1
OPP	t0 vs. t6	t0	t6	13	Prevotella	Prevotella_shahii	0.0396	0.0856	0.0279	0.0516	0.6883	1
OPP	t0 vs. t6	t0	t6	13	Prevotella	Prevotella_veroralis	0.0543	0.1957	0.0416	0.1492	0.3444	1
OPP	t0 vs. t6	t0	t6	13	Tannerella	Tannerella_forsythia	0.0032	0.0066	0.0093	0.0210	0.3591	1
OPP	t0 vs. t6	t0	t6	13	Capnocytophaga	Capnocytophaga_gingivalis	0.0344	0.0624	0.0306	0.0304	0.8122	1
OPP	t0 vs. t6	t0	t6	13	Capnocytophaga	Capnocytophaga_granulosa	0.0028	0.0062	0.0052	0.0132	0.5634	1
OPP	t0 vs. t6	t0	t6	13	Capnocytophaga	Capnocytophaga_leadbetteri	0.0270	0.0370	0.0538	0.0990	0.4116	1
OPP	t0 vs. t6	t0	t6	13	Capnocytophaga	Capnocytophaga_ochracea	0.0252	0.0439	0.0567	0.1197	0.3856	1
OPP	t0 vs. t6	t0	t6	13	Capnocytophaga	Capnocytophaga_sputigena	0.1528	0.2807	0.1179	0.2386	0.5983	1
OPP	t0 vs. t6	t0	t6	13	Flavobacterium	Flavobacterium_branchiophilum	0.0005	0.0013	0.0001	0.0005	0.3489	1
OPP	t0 vs. t6	t0	t6	13	Bergeyella	Flavobacteriaceae_bacterium	0.0000	0.0001	0.0005	0.0017	0.3625	1
OPP	t0 vs. t6	t0	t6	13	Campylobacter	Campylobacter_concisus	0.0248	0.0270	0.0780	0.1334	0.1619	1
OPP	t0 vs. t6	t0	t6	13	Campylobacter	Campylobacter_showae	0.0005	0.0008	0.0003	0.0009	0.5876	1
OPP	t0 vs. t6	t0	t6	13	Solobacterium	Solobacterium_moorei	0.2233	0.4085	0.1358	0.1795	0.5098	1
OPP	t0 vs. t6	t0	t6	13	Globicatella	Aerococcaceae_bacterium	0.0017	0.0036	0.0012	0.0045	0.8021	1
OPP	t0 vs. t6	t0	t6	13	Granulicatella	Granulicatella_adiacens	0.0127	0.0179	0.0043	0.0129	0.2439	1
OPP	t0 vs. t6	t0	t6	13	Granulicatella	Granulicatella_elegans	0.0180	0.0267	0.0263	0.0389	0.4360	1
OPP	t0 vs. t6	t0	t6	13	Lactobacillus	Lactobacillus_curvatus	0.0015	0.0055	0.0002	0.0006	0.3955	1
OPP	t0 vs. t6	t0	t6	13	Lactobacillus	Lactobacillus_sakei	0.0023	0.0082	0.0022	0.0054	0.9591	1
OPP	t0 vs. t6	t0	t6	13	Lactococcus	Lactococcus_lactis	0.0016	0.0050	0.5487	1.9767	0.3380	1

OPP	t0 vs. t6	t0	t6	13	Streptococcus	Streptococcus_anginosus	0.0035	0.0093	0.0215	0.0469	0.2106	1
OPP	t0 vs. t6	t0	t6	13	Streptococcus	Streptococcus_australis	0.0092	0.0195	0.0024	0.0037	0.2181	1
OPP	t0 vs. t6	t0	t6	13	Streptococcus	Streptococcus_cristatus	0.0319	0.0715	0.0121	0.0172	0.3436	1
OPP	t0 vs. t6	t0	t6	13	Streptococcus	Streptococcus_gordonii	0.0296	0.0321	0.0192	0.0235	0.3236	1
OPP	t0 vs. t6	t0	t6	13	Streptococcus	Streptococcus_infantis	0.0271	0.0355	0.0273	0.0186	0.9850	1
OPP	t0 vs. t6	t0	t6	13	Streptococcus	Streptococcus_mitis	0.0054	0.0111	0.0033	0.0097	0.6376	1
OPP	t0 vs. t6	t0	t6	13	Streptococcus	Streptococcus_mutans	0.0001	0.0003	0.0022	0.0080	0.3551	1
OPP	t0 vs. t6	t0	t6	13	Streptococcus	Streptococcus_oralis	0.0098	0.0164	0.0111	0.0237	0.8431	1
OPP	t0 vs. t6	t0	t6	13	Streptococcus	Streptococcus_parasanguinis	1.1189	0.7326	1.4499	1.3725	0.4067	1
OPP	t0 vs. t6	t0	t6	13	Streptococcus	Streptococcus_peroris	0.0018	0.0066	0.0013	0.0048	0.8331	1
OPP	t0 vs. t6	t0	t6	13	Streptococcus	Streptococcus_pneumoniae	0.2536	0.2907	0.2292	0.2262	0.8167	1
OPP	t0 vs. t6	t0	t6	13	Streptococcus	Streptococcus_salivarius	14.0242	12.2038	4.4911	3.2065	0.0198	1
OPP	t0 vs. t6	t0	t6	13	Streptococcus	Streptococcus_sanguinis	0.1046	0.2000	0.0534	0.0739	0.4306	1
OPP	t0 vs. t6	t0	t6	13	Streptococcus	Streptococcus_suis	0.2324	0.2485	0.2674	0.2651	0.7701	1
OPP	t0 vs. t6	t0	t6	13	Gemella	Gemella_haemolysans	1.0406	0.9821	1.1362	0.7118	0.7120	1
OPP	t0 vs. t6	t0	t6	13	Gemella	Gemella_morbillorum	0.0127	0.0260	0.0124	0.0205	0.9769	1
OPP	t0 vs. t6	t0	t6	13	Gemella	Gemella_sanguinis	0.0535	0.0870	0.0739	0.0701	0.4960	1
OPP	t0 vs. t6	t0	t6	13	Clostridia_UCG-014	Clostridiales_bacterium	0.3373	0.4897	0.1398	0.2673	0.0342	1
OPP	t0 vs. t6	t0	t6	13	Defluviitaleaceae_UCG-011	Lachnospiraceae_bacterium	0.0025	0.0091	0.0005	0.0018	0.3370	1
OPP	t0 vs. t6	t0	t6	13	Catonella	Catonella_morbi	0.0324	0.0310	0.0336	0.0450	0.9429	1
OPP	t0 vs. t6	t0	t6	13	Johnsonella	Lachnospiraceae_bacterium	0.0126	0.0305	0.0018	0.0045	0.1892	1
OPP	t0 vs. t6	t0	t6	13	Lachnoanaerobaculum	Lachnoanaerobaculum_umeaense	0.3206	0.5683	0.1601	0.1643	0.3224	1
OPP	t0 vs. t6	t0	t6	13	Lachnobacterium	Lachnobacterium_bovis	0.0025	0.0088	0.0021	0.0077	0.9273	1
OPP	t0 vs. t6	t0	t6	13	Stomatobaculum	Stomatobaculum_longum	0.0870	0.1405	0.0610	0.0916	0.5326	1
OPP	t0 vs. t6	t0	t6	13	[Eubacterium]_brachy_group	[Eubacterium]_brachy	0.0002	0.0006	0.0014	0.0031	0.1621	1
OPP	t0 vs. t6	t0	t6	13	[Eubacterium]_yurii_group	[Eubacterium]_yurii	0.0152	0.0335	0.0073	0.0170	0.4889	1
OPP	t0 vs. t6	t0	t6	13	Parvimonas	Parvimonas_micra	0.0010	0.0019	0.0006	0.0011	0.5668	1
OPP	t0 vs. t6	t0	t6	13	Selenomonas	Selenomonas_flueggei	0.0004	0.0014	0.0107	0.0197	0.0878	1
OPP	t0 vs. t6	t0	t6	13	Selenomonas	Selenomonas_infelix	0.0002	0.0006	0.0047	0.0125	0.2122	1
OPP	t0 vs. t6	t0	t6	13	Selenomonas	Selenomonas_massiliensis	0.0006	0.0021	0.0036	0.0073	0.1996	1
OPP	t0 vs. t6	t0	t6	13	Selenomonas	Selenomonas_noxia	0.0031	0.0112	0.0077	0.0135	0.3903	1
OPP	t0 vs. t6	t0	t6	13	Selenomonas	Selenomonas_sputigena	0.0045	0.0117	0.0426	0.1293	0.3171	1
OPP	t0 vs. t6	t0	t6	13	Dialister	Dialister_invisus	0.0010	0.0031	0.0014	0.0033	0.7872	1
OPP	t0 vs. t6	t0	t6	13	Dialister	Dialister_pneumosintes	0.0005	0.0017	0.0035	0.0070	0.0908	1
OPP	t0 vs. t6	t0	t6	13	Megasphaera	Megasphaera_micronuciformis	0.1742	0.3846	0.1182	0.1808	0.6483	1
OPP	t0 vs. t6	t0	t6	13	Veillonella	Veillonella_atypica	1.2368	1.9212	1.4086	1.5301	0.7999	1
OPP	t0 vs. t6	t0	t6	13	Veillonella	Veillonella_parvula	3.6161	4.1241	2.7720	1.9861	0.4168	1
OPP	t0 vs. t6	t0	t6	13	Veillonella	Veillonella_rogosae	0.0910	0.2187	0.0002	0.0006	0.1601	1
OPP	t0 vs. t6	t0	t6	13	Fusobacterium	Fusobacterium_nucleatum	0.3753	0.3826	0.7043	0.6720	0.1408	1
OPP	t0 vs. t6	t0	t6	13	Fusobacterium	Fusobacterium_periodonticum	1.1427	1.1595	1.3613	1.1260	0.5888	1
OPP	t0 vs. t6	t0	t6	13	Leptotrichia	Leptotrichia_buccalis	0.2167	0.3217	0.1546	0.2447	0.5887	1
OPP	t0 vs. t6	t0	t6	13	Leptotrichia	Leptotrichia_hofstadii	0.0041	0.0096	0.0010	0.0020	0.2784	1
OPP	t0 vs. t6	t0	t6	13	Leptotrichia	Leptotrichia_hongkongensis	0.0003	0.0004	0.0082	0.0204	0.1869	1
OPP	t0 vs. t6	t0	t6	13	Leptotrichia	Leptotrichia_shahii	0.0107	0.0228	0.0401	0.1118	0.3709	1
OPP	t0 vs. t6	t0	t6	13	Leptotrichia	Leptotrichia_trevisanii	0.0006	0.0022	0.0101	0.0322	0.3137	1
OPP	t0 vs. t6	t0	t6	13	Leptotrichia	Leptotrichia_wadei	0.2124	0.3297	0.5644	1.0911	0.2344	1
OPP	t0 vs. t6	t0	t6	13	Absconditabacteriales_(SR1)	SR1_bacterium	0.0117	0.0259	0.0044	0.0152	0.0690	1
OPP	t0 vs. t6	t0	t6	13	Gracilibacteria	Gracilibacteria_bacterium	0.0004	0.0010	0.0007	0.0015	0.4346	1
OPP	t0 vs. t6	t0	t6	13	JGI_0000069-P22	Gracilibacteria_bacterium	0.0001	0.0002	0.0013	0.0025	0.0967	1
OPP	t0 vs. t6	t0	t6	13	Candidatus_Saccharimonas	Candidatus_Saccharimonas	0.0002	0.0009	0.0002	0.0008	0.9295	1

OPP	t0 vs. t6	t0	t6	13	Candidatus_Saccharimonas	TM7_phylum	0.0294	0.0579	0.0324	0.0483	0.8645	1
OPP	t0 vs. t6	t0	t6	13	Saccharimonadaceae	TM7_bacterium	0.0113	0.0260	0.0008	0.0017	0.1773	1
OPP	t0 vs. t6	t0	t6	13	Saccharimonadaceae	TM7_phylum	0.0041	0.0148	0.0004	0.0014	0.3881	1
OPP	t0 vs. t6	t0	t6	13	TM7a	candidate_division	0.0079	0.0228	0.0002	0.0003	0.2424	1
OPP	t0 vs. t6	t0	t6	13	TM7a	TM7_phylum	0.0020	0.0073	0.0007	0.0019	0.4026	1
OPP	t0 vs. t6	t0	t6	13	TM7x	Candidatus_Saccharibacteria	0.0654	0.1524	0.1117	0.2389	0.5662	1
OPP	t0 vs. t6	t0	t6	13	TM7x	TM7_phylum	0.0655	0.0849	0.3549	0.7214	0.1833	1
OPP	t0 vs. t6	t0	t6	13	Saccharimonadales	TM7_phylum	0.1245	0.2438	0.0902	0.1615	0.5278	1
OPP	t0 vs. t6	t0	t6	13	Shewanella	Shewanella_putrefaciens	0.0043	0.0073	0.0016	0.0032	0.2015	1
OPP	t0 vs. t6	t0	t6	13	Lautropia	Lautropia_mirabilis	0.0404	0.0870	0.0331	0.0337	0.7823	1
OPP	t0 vs. t6	t0	t6	13	Brachymonas	Brachymonas_denitrificans	0.0103	0.0323	0.0013	0.0020	0.3185	1
OPP	t0 vs. t6	t0	t6	13	Eikenella	Eikenella_corrodens	0.0003	0.0009	0.0006	0.0017	0.5022	1
OPP	t0 vs. t6	t0	t6	13	Neisseria	Neisseria_bacilliformis	0.0015	0.0042	0.0019	0.0045	0.8067	1
OPP	t0 vs. t6	t0	t6	13	Neisseria	Neisseria_elongata	0.0183	0.0215	0.0101	0.0153	0.0828	1
OPP	t0 vs. t6	t0	t6	13	Neisseria	Neisseria_meningitidis	7.1280	6.5437	9.4015	6.9496	0.4222	1
OPP	t0 vs. t6	t0	t6	13	Neisseria	Neisseria_oralis	0.0002	0.0008	0.0053	0.0082	0.0418	1
OPP	t0 vs. t6	t0	t6	13	Neisseria	Neisseria_perflava	0.0016	0.0035	0.0756	0.1856	0.1766	1
OPP	t0 vs. t6	t0	t6	13	Simonsiella	Simonsiella_muelleri	0.0029	0.0055	0.0004	0.0011	0.1603	1
OPP	t0 vs. t6	t0	t6	13	Cardiobacterium	Cardiobacterium_hominis	0.0010	0.0015	0.0086	0.0193	0.1711	1
OPP	t0 vs. t6	t0	t6	13	Cardiobacterium	Cardiobacterium_valvarum	0.0017	0.0029	0.0053	0.0079	0.1413	1
OPP	t0 vs. t6	t0	t6	13	Actinobacillus	Actinobacillus_pleuropneumoniae	0.0108	0.0330	0.0212	0.0259	0.1969	1
OPP	t0 vs. t6	t0	t6	13	Actinobacillus	Haemophilus_parahaemolyticus	0.0129	0.0237	0.0060	0.0120	0.4053	1
OPP	t0 vs. t6	t0	t6	13	Aggregatibacter	Aggregatibacter_aphrophilus	0.0304	0.0485	0.0420	0.0461	0.2145	1
OPP	t0 vs. t6	t0	t6	13	Aggregatibacter	Aggregatibacter_segnis	0.0104	0.0141	0.0326	0.0540	0.1933	1
OPP	t0 vs. t6	t0	t6	13	Haemophilus	[Haemophilus]_ducreyi	0.0016	0.0040	0.0013	0.0033	0.8465	1
OPP	t0 vs. t6	t0	t6	13	Haemophilus	Haemophilus_haemolyticus	0.0325	0.0552	0.0385	0.0956	0.8199	1
OPP	t0 vs. t6	t0	t6	13	Haemophilus	Haemophilus_influenzae	0.0258	0.0418	0.0374	0.0811	0.6309	1
OPP	t0 vs. t6	t0	t6	13	Haemophilus	Haemophilus_parainfluenzae	1.4566	1.5257	1.4579	1.1036	0.9971	1
OPP	t0 vs. t6	t0	t6	13	Haemophilus	Haemophilus_pittmaniae	0.7525	0.7667	0.7673	0.8439	0.8790	1
OPP	t0 vs. t6	t0	t6	13	Haemophilus	Haemophilus_sputorum	0.0235	0.0448	0.0389	0.0688	0.2313	1
OPP	t0 vs. t6	t0	t6	13	Mannheimia	Mannheimia_haemolytica	0.0016	0.0040	0.0249	0.0303	0.0195	1
OPP	t0 vs. t6	t0	t6	13	Pasteurella	Pasteurella_multocida	0.5407	0.6921	0.7310	0.6183	0.4931	1
OPP	t0 vs. t6	t0	t6	13	Rodentibacter	Pasteurellaceae_bacterium	0.0020	0.0048	0.0788	0.1425	0.0713	1
OPP	t0 vs. t6	t0	t6	13	Treponema	Treponema_medium	0.0022	0.0054	0.0001	0.0003	0.1907	1
OPP	t3 vs. t6	t3	t6	12	Actinomyces	Actinomyces_graevenitzii	0.5928	1.1885	0.7709	1.2331	0.3864	1
OPP	t3 vs. t6	t3	t6	12	Actinomyces	Actinomyces_naeslundii	0.0093	0.0084	0.0568	0.0880	0.0835	1
OPP	t3 vs. t6	t3	t6	12	Actinomyces	Actinomyces_oris	0.0004	0.0012	0.0022	0.0042	0.2024	1
OPP	t3 vs. t6	t3	t6	12	Actinomyces	Schaalia_odontolytica	0.2235	0.4406	0.2234	0.3161	0.9995	1
OPP	t3 vs. t6	t3	t6	12	Alloscardovia	Alloscardovia_omnicolens	0.0093	0.0229	0.0864	0.2950	0.3708	1
OPP	t3 vs. t6	t3	t6	12	Corynebacterium	Corynebacterium_diphtheriae	0.0003	0.0006	0.0019	0.0035	0.1447	1
OPP	t3 vs. t6	t3	t6	12	Corynebacterium	Corynebacterium_durum	0.0016	0.0031	0.0042	0.0065	0.1008	1
OPP	t3 vs. t6	t3	t6	12	Corynebacterium	Corynebacterium_matruchotii	0.0011	0.0025	0.0082	0.0160	0.1141	1
OPP	t3 vs. t6	t3	t6	12	Rothia	Rothia_aeria	0.0858	0.1447	0.0826	0.1311	0.8623	1
OPP	t3 vs. t6	t3	t6	12	Rothia	Rothia_mucilaginosa	6.2384	12.6271	5.7832	5.9930	0.8749	1
OPP	t3 vs. t6	t3	t6	12	Atopobium	Lancefieldella_parvula	0.0227	0.0507	0.0186	0.0233	0.7820	1
OPP	t3 vs. t6	t3	t6	12	Bacteroides	Bacteroidaceae_bacterium	0.0005	0.0012	0.0009	0.0021	0.5423	1
OPP	t3 vs. t6	t3	t6	12	F0058	Bacteroidetes_oral	0.0009	0.0027	0.0009	0.0017	0.9447	1
OPP	t3 vs. t6	t3	t6	12	Porphyromonas	Porphyromonas_catoniae	0.0085	0.0220	0.0122	0.0208	0.2402	1
OPP	t3 vs. t6	t3	t6	12	Porphyromonas	Porphyromonas_endodontalis	0.0041	0.0099	0.0095	0.0221	0.2037	1
OPP	t3 vs. t6	t3	t6	12	Porphyromonas	Porphyromonas_gingivalis	0.0017	0.0050	0.0008	0.0020	0.4249	1

OPP	t3 vs. t6	t3	t6	12	Porphyromonas	Porphyromonas_pasteri	2.2100	2.4185	1.1690	1.1071	0.1256	1
OPP	t3 vs. t6	t3	t6	12	Alloprevotella	Alloprevotella_rava	0.4984	1.4488	0.1163	0.4021	0.2364	1
OPP	t3 vs. t6	t3	t6	12	Alloprevotella	Alloprevotella_tannerae	0.0694	0.1494	0.0729	0.1768	0.9562	1
OPP	t3 vs. t6	t3	t6	12	Alloprevotella	Prevotellaceae_bacterium	1.1244	1.6193	0.6593	0.7421	0.2116	1
OPP	t3 vs. t6	t3	t6	12	Prevotella	Prevotella_buccae	0.0001	0.0004	0.0001	0.0003	0.3388	1
OPP	t3 vs. t6	t3	t6	12	Prevotella	Prevotella_denticola	0.0003	0.0008	0.0070	0.0167	0.1916	1
OPP	t3 vs. t6	t3	t6	12	Prevotella	Prevotella_fusca	0.0052	0.0162	0.0070	0.0140	0.4759	1
OPP	t3 vs. t6	t3	t6	12	Prevotella	Prevotella_histicola	0.0741	0.1712	0.0394	0.0931	0.5517	1
OPP	t3 vs. t6	t3	t6	12	Prevotella	Prevotella_intermedia	0.0963	0.2055	0.1110	0.1857	0.8554	1
OPP	t3 vs. t6	t3	t6	12	Prevotella	Prevotella_jejuni	0.0664	0.2135	0.0834	0.1635	0.7925	1
OPP	t3 vs. t6	t3	t6	12	Prevotella	Prevotella_loescheii	0.1344	0.1957	0.3240	0.9180	0.4108	1
OPP	t3 vs. t6	t3	t6	12	Prevotella	Prevotella_maculosa	0.0006	0.0014	0.0004	0.0011	0.2516	1
OPP	t3 vs. t6	t3	t6	12	Prevotella	Prevotella_melaninogenica	5.8417	4.7901	11.5480	10.5459	0.0288	1
OPP	t3 vs. t6	t3	t6	12	Prevotella	Prevotella_micans	0.0008	0.0028	0.0004	0.0013	0.3388	1
OPP	t3 vs. t6	t3	t6	12	Prevotella	Prevotella_nanceiensis	0.4225	0.5868	0.3613	0.4454	0.7754	1
OPP	t3 vs. t6	t3	t6	12	Prevotella	Prevotella_nigrescens	0.0300	0.0741	0.0184	0.0409	0.4301	1
OPP	t3 vs. t6	t3	t6	12	Prevotella	Prevotella_oulorum	0.0293	0.0465	0.0489	0.1034	0.4400	1
OPP	t3 vs. t6	t3	t6	12	Prevotella	Prevotella_pallens	0.5608	0.8693	0.5755	1.2182	0.9626	1
OPP	t3 vs. t6	t3	t6	12	Prevotella	Prevotella_saccharolytica	0.0004	0.0010	0.0006	0.0013	0.7500	1
OPP	t3 vs. t6	t3	t6	12	Prevotella	Prevotella_salivae	0.5179	0.6443	0.4068	0.4639	0.5395	1
OPP	t3 vs. t6	t3	t6	12	Prevotella	Prevotella_shahii	0.0660	0.1133	0.0164	0.0398	0.1509	1
OPP	t3 vs. t6	t3	t6	12	Prevotella	Prevotella_veroralis	0.1232	0.4212	0.0451	0.1553	0.3306	1
OPP	t3 vs. t6	t3	t6	12	Tannerella	Tannerella_forsythia	0.0042	0.0123	0.0095	0.0219	0.5085	1
OPP	t3 vs. t6	t3	t6	12	Capnocytophaga	Capnocytophaga_gingivalis	0.0561	0.0574	0.0315	0.0318	0.0520	1
OPP	t3 vs. t6	t3	t6	12	Capnocytophaga	Capnocytophaga_granulosa	0.0056	0.0111	0.0052	0.0137	0.8808	1
OPP	t3 vs. t6	t3	t6	12	Capnocytophaga	Capnocytophaga_leadbetteri	0.0449	0.0437	0.0515	0.1033	0.8448	1
OPP	t3 vs. t6	t3	t6	12	Capnocytophaga	Capnocytophaga_ochracea	0.0293	0.0318	0.0582	0.1251	0.4745	1
OPP	t3 vs. t6	t3	t6	12	Capnocytophaga	Capnocytophaga_sputigena	0.1079	0.1824	0.1221	0.2490	0.8038	1
OPP	t3 vs. t6	t3	t6	12	Bergeyella	Flavobacteriaceae_bacterium	0.0001	0.0003	0.0005	0.0018	0.3388	1
OPP	t3 vs. t6	t3	t6	12	Campylobacter	Campylobacter_concisus	0.0572	0.0751	0.0840	0.1375	0.5579	1
OPP	t3 vs. t6	t3	t6	12	Campylobacter	Campylobacter_showae	0.0019	0.0042	0.0003	0.0010	0.1864	1
OPP	t3 vs. t6	t3	t6	12	Solobacterium	Solobacterium_moorei	0.1079	0.0987	0.1059	0.1270	0.9647	1
OPP	t3 vs. t6	t3	t6	12	Globicatella	Aerococcaceae_bacterium	0.0034	0.0079	0.0014	0.0047	0.4772	1
OPP	t3 vs. t6	t3	t6	12	Granulicatella	Granulicatella_adiacens	0.0038	0.0086	0.0039	0.0134	0.9965	1
OPP	t3 vs. t6	t3	t6	12	Granulicatella	Granulicatella_elegans	0.0211	0.0215	0.0198	0.0378	0.8886	1
OPP	t3 vs. t6	t3	t6	12	Lactobacillus	Lactobacillus_sakei	0.0113	0.0269	0.0024	0.0056	0.3038	1
OPP	t3 vs. t6	t3	t6	12	Lactococcus	Lactococcus_lactis	0.0168	0.0533	0.5974	2.0565	0.3504	1
OPP	t3 vs. t6	t3	t6	12	Streptococcus	Streptococcus_anginosus	0.0052	0.0135	0.0223	0.0489	0.2798	1
OPP	t3 vs. t6	t3	t6	12	Streptococcus	Streptococcus_australis	0.0052	0.0083	0.0025	0.0038	0.3025	1
OPP	t3 vs. t6	t3	t6	12	Streptococcus	Streptococcus_cristatus	0.0286	0.0397	0.0131	0.0175	0.2658	1
OPP	t3 vs. t6	t3	t6	12	Streptococcus	Streptococcus_gordonii	0.0168	0.0219	0.0156	0.0236	0.9064	1
OPP	t3 vs. t6	t3	t6	12	Streptococcus	Streptococcus_infantis	0.0356	0.0379	0.0247	0.0193	0.3211	1
OPP	t3 vs. t6	t3	t6	12	Streptococcus	Streptococcus_mitis	0.0111	0.0185	0.0058	0.0121	0.4507	1
OPP	t3 vs. t6	t3	t6	12	Streptococcus	Streptococcus_oralis	0.0048	0.0117	0.0049	0.0081	0.9956	1
OPP	t3 vs. t6	t3	t6	12	Streptococcus	Streptococcus_parasanguinis	2.5052	3.4854	1.6414	1.4927	0.2753	1
OPP	t3 vs. t6	t3	t6	12	Streptococcus	Streptococcus_peroris	0.0007	0.0023	0.0014	0.0050	0.6462	1
OPP	t3 vs. t6	t3	t6	12	Streptococcus	Streptococcus_pneumoniae	0.2771	0.2816	0.2376	0.2318	0.7128	1
OPP	t3 vs. t6	t3	t6	12	Streptococcus	Streptococcus_salivarius	6.7699	8.2053	4.0668	3.0333	0.3288	1
OPP	t3 vs. t6	t3	t6	12	Streptococcus	Streptococcus_sanguinis	0.2177	0.4949	0.0535	0.0771	0.2926	1
OPP	t3 vs. t6	t3	t6	12	Streptococcus	Streptococcus_suis	0.2137	0.1886	0.2696	0.2769	0.5664	1

OPP	t3 vs. t6	t3	t6	12	Gemella	Gemella_haemolysans	1.3046	0.8688	1.1904	0.8104	0.6216	1
OPP	t3 vs. t6	t3	t6	12	Gemella	Gemella_morbillorum	0.0130	0.0256	0.0075	0.0088	0.3887	1
OPP	t3 vs. t6	t3	t6	12	Gemella	Gemella_sanguinis	0.0964	0.0719	0.0756	0.0689	0.3030	1
OPP	t3 vs. t6	t3	t6	12	Clostridia_UCG-014	Clostridiales_bacterium	0.3846	0.7358	0.0583	0.0974	0.1531	1
OPP	t3 vs. t6	t3	t6	12	Catonella	Catonella_morbi	0.0280	0.0256	0.0523	0.0876	0.3504	1
OPP	t3 vs. t6	t3	t6	12	Johnsonella	Lachnospiraceae_bacterium	0.0051	0.0123	0.0011	0.0039	0.1661	1
OPP	t3 vs. t6	t3	t6	12	Lachnoanaerobaculum	Lachnoanaerobaculum_umeaense	0.1989	0.3736	0.1590	0.1679	0.7496	1
OPP	t3 vs. t6	t3	t6	12	Stomatobaculum	Stomatobaculum_longum	0.0640	0.0975	0.0800	0.1025	0.6226	1
OPP	t3 vs. t6	t3	t6	12	[Eubacterium]_brachy_group	[Eubacterium]_brachy	0.0009	0.0030	0.0009	0.0021	0.9521	1
OPP	t3 vs. t6	t3	t6	12	[Eubacterium]_yurii_group	[Eubacterium]_yurii	0.0064	0.0116	0.0022	0.0044	0.1846	1
OPP	t3 vs. t6	t3	t6	12	Filifactor	Filifactor_alocis	0.0004	0.0015	0.0068	0.0236	0.3720	1
OPP	t3 vs. t6	t3	t6	12	Parvimonas	Parvimonas_micra	0.0003	0.0005	0.0006	0.0011	0.2900	1
OPP	t3 vs. t6	t3	t6	12	Selenomonas	Selenomonas_flueggei	0.0016	0.0050	0.0116	0.0204	0.1386	1
OPP	t3 vs. t6	t3	t6	12	Selenomonas	Selenomonas_massiliensis	0.0047	0.0141	0.0032	0.0072	0.7224	1
OPP	t3 vs. t6	t3	t6	12	Selenomonas	Selenomonas_noxia	0.0188	0.0348	0.0111	0.0169	0.4864	1
OPP	t3 vs. t6	t3	t6	12	Selenomonas	Selenomonas_sputigena	0.0222	0.0553	0.0450	0.1348	0.6106	1
OPP	t3 vs. t6	t3	t6	12	Dialister	Dialister_invisus	0.0012	0.0039	0.0010	0.0028	0.8988	1
OPP	t3 vs. t6	t3	t6	12	Dialister	Dialister_pneumosintes	0.0033	0.0114	0.0030	0.0070	0.8963	1
OPP	t3 vs. t6	t3	t6	12	Megasphaera	Megasphaera_micronuciformis	0.1094	0.1431	0.1334	0.1823	0.7380	1
OPP	t3 vs. t6	t3	t6	12	Veillonella	Veillonella_atypica	1.0578	1.5316	1.7442	1.8199	0.3195	1
OPP	t3 vs. t6	t3	t6	12	Veillonella	Veillonella_parvula	2.2008	1.4712	2.5261	1.8745	0.5631	1
OPP	t3 vs. t6	t3	t6	12	Veillonella	Veillonella_rogosae	0.0033	0.0114	0.0002	0.0006	0.3655	1
OPP	t3 vs. t6	t3	t6	12	Fusobacterium	Fusobacterium_nucleatum	0.6773	0.7070	0.6911	0.7056	0.9516	1
OPP	t3 vs. t6	t3	t6	12	Fusobacterium	Fusobacterium_periodonticum	1.6530	1.3183	1.3154	1.1669	0.3066	1
OPP	t3 vs. t6	t3	t6	12	Leptotrichia	Leptotrichia_buccalis	0.1977	0.4438	0.1273	0.2353	0.6540	1
OPP	t3 vs. t6	t3	t6	12	Leptotrichia	Leptotrichia_hofstadii	0.0010	0.0022	0.0006	0.0014	0.6067	1
OPP	t3 vs. t6	t3	t6	12	Leptotrichia	Leptotrichia_hongkongensis	0.0010	0.0013	0.0090	0.0211	0.2133	1
OPP	t3 vs. t6	t3	t6	12	Leptotrichia	Leptotrichia_shahii	0.0048	0.0114	0.0381	0.1164	0.3532	1
OPP	t3 vs. t6	t3	t6	12	Leptotrichia	Leptotrichia_trevisanii	0.0002	0.0007	0.0109	0.0335	0.2896	1
OPP	t3 vs. t6	t3	t6	12	Leptotrichia	Leptotrichia_wadei	0.1802	0.3543	0.5693	1.1388	0.2943	1
OPP	t3 vs. t6	t3	t6	12	Streptobacillus	Streptobacillus_hongkongensis	0.0000	0.0001	0.0003	0.0012	0.4070	1
OPP	t3 vs. t6	t3	t6	12	Absconditabacteriales_(SR1)	SR1_bacterium	0.0080	0.0144	0.0048	0.0158	0.4410	1
OPP	t3 vs. t6	t3	t6	12	Gracilibacteria	Gracilibacteria_bacterium	0.0008	0.0020	0.0005	0.0013	0.5413	1
OPP	t3 vs. t6	t3	t6	12	JGI_0000069-P22	Gracilibacteria_bacterium	0.0009	0.0019	0.0011	0.0025	0.8131	1
OPP	t3 vs. t6	t3	t6	12	Candidatus_Saccharimonas	TM7_phylum	0.0583	0.0781	0.0245	0.0485	0.0772	1
OPP	t3 vs. t6	t3	t6	12	Saccharimonadaceae	TM7_bacterium	0.0064	0.0188	0.0007	0.0017	0.3288	1
OPP	t3 vs. t6	t3	t6	12	Saccharimonadaceae	TM7_phylum	0.0027	0.0095	0.0004	0.0014	0.4259	1
OPP	t3 vs. t6	t3	t6	12	TM7a	candidate_division	0.0084	0.0275	0.0002	0.0003	0.3217	1
OPP	t3 vs. t6	t3	t6	12	TM7a	TM7_phylum	0.0005	0.0011	0.0008	0.0020	0.4199	1
OPP	t3 vs. t6	t3	t6	12	TM7x	Candidatus_Saccharibacteria	0.1047	0.1455	0.1114	0.2503	0.9199	1
OPP	t3 vs. t6	t3	t6	12	TM7x	TM7_phylum	0.1492	0.2373	0.1935	0.4593	0.7787	1
OPP	t3 vs. t6	t3	t6	12	Saccharimonadales	TM7_phylum	0.0956	0.1589	0.0660	0.1554	0.6318	1
OPP	t3 vs. t6	t3	t6	12	Shewanella	Shewanella_putrefaciens	0.0008	0.0013	0.0012	0.0030	0.6812	1
OPP	t3 vs. t6	t3	t6	12	Lautropia	Lautropia_mirabilis	0.0397	0.0738	0.0279	0.0317	0.5723	1
OPP	t3 vs. t6	t3	t6	12	Brachymonas	Brachymonas_denitrificans	0.0013	0.0029	0.0014	0.0021	0.9496	1
OPP	t3 vs. t6	t3	t6	12	Eikenella	Eikenella_corrodens	0.0004	0.0013	0.0007	0.0017	0.1911	1
OPP	t3 vs. t6	t3	t6	12	Neisseria	Neisseria_bacilliformis	0.0016	0.0055	0.0020	0.0046	0.6332	1
OPP	t3 vs. t6	t3	t6	12	Neisseria	Neisseria_elongata	0.0319	0.0850	0.0103	0.0157	0.3786	1
OPP	t3 vs. t6	t3	t6	12	Neisseria	Neisseria_meningitidis	10.8647	9.5780	9.2947	7.0782	0.4345	1
OPP	t3 vs. t6	t3	t6	12	Neisseria	Neisseria_oralis	0.0063	0.0183	0.0076	0.0102	0.8379	1

OPP	t3 vs. t6	t3	t6	12	Neisseria	Neisseria_perflava	0.0460	0.1395	0.2047	0.4449	0.1188	1
OPP	t3 vs. t6	t3	t6	12	Simonsiella	Simonsiella_muelleri	0.0027	0.0062	0.0005	0.0012	0.2208	1
OPP	t3 vs. t6	t3	t6	12	Cardiobacterium	Cardiobacterium_hominis	0.0039	0.0067	0.0089	0.0200	0.2387	1
OPP	t3 vs. t6	t3	t6	12	Cardiobacterium	Cardiobacterium_valvarum	0.0025	0.0043	0.0053	0.0083	0.0748	1
OPP	t3 vs. t6	t3	t6	12	Escherichia-Shigella	Escherichia_coli	0.0005	0.0017	0.0001	0.0003	0.4500	1
OPP	t3 vs. t6	t3	t6	12	Actinobacillus	Actinobacillus_pleuropneumoniae	0.0247	0.0420	0.0216	0.0272	0.7979	1
OPP	t3 vs. t6	t3	t6	12	Actinobacillus	Haemophilus_parahaemolyticus	0.0130	0.0367	0.0035	0.0050	0.3697	1
OPP	t3 vs. t6	t3	t6	12	Aggregatibacter	Aggregatibacter_aphrophilus	0.0531	0.0987	0.0391	0.0491	0.6705	1
OPP	t3 vs. t6	t3	t6	12	Aggregatibacter	Aggregatibacter_segnis	0.0297	0.0585	0.0204	0.0289	0.5549	1
OPP	t3 vs. t6	t3	t6	12	Haemophilus	[Haemophilus]_ducreyi	0.0021	0.0034	0.0007	0.0023	0.0784	1
OPP	t3 vs. t6	t3	t6	12	Haemophilus	Haemophilus_haemolyticus	0.0176	0.0210	0.0408	0.0995	0.3937	1
OPP	t3 vs. t6	t3	t6	12	Haemophilus	Haemophilus_influenzae	0.0515	0.0765	0.0418	0.0834	0.6757	1
OPP	t3 vs. t6	t3	t6	12	Haemophilus	Haemophilus_parainfluenzae	1.5463	1.0615	1.3675	1.1016	0.6876	1
OPP	t3 vs. t6	t3	t6	12	Haemophilus	Haemophilus_pittmaniae	0.8267	0.7929	0.7644	0.8915	0.8635	1
OPP	t3 vs. t6	t3	t6	12	Haemophilus	Haemophilus_sputorum	0.0200	0.0417	0.0412	0.0713	0.1461	1
OPP	t3 vs. t6	t3	t6	12	Mannheimia	Mannheimia_haemolytica	0.0083	0.0133	0.0180	0.0244	0.2254	1
OPP	t3 vs. t6	t3	t6	12	Pasteurella	Pasteurella_multocida	0.6941	0.6510	0.6606	0.5923	0.8812	1
OPP	t3 vs. t6	t3	t6	12	Rodentibacter	Pasteurellaceae_bacterium	0.0160	0.0514	0.0773	0.1497	0.1594	1
OPP	t3 vs. t6	t3	t6	12	Acinetobacter	Acinetobacter_johnsonii	0.0011	0.0018	0.0009	0.0025	0.8121	1
OPP	t3 vs. t6	t3	t6	12	Moraxella	Moraxella_catarrhalis	0.0003	0.0012	0.0001	0.0002	0.4827	1
OPP	t3 vs. t6	t3	t6	12	Treponema	Treponema_medium	0.0025	0.0053	0.0001	0.0003	0.1322	1
TAU	t0 vs. t3	t0	t3	15	Actinomyces	Actinomyces_dentalis	0.0000	0.0001	0.0006	0.0016	0.1785	1
TAU	t0 vs. t3	t0	t3	15	Actinomyces	Actinomyces_graevenitzii	1.0468	1.2833	0.7870	1.2152	0.5051	1
TAU	t0 vs. t3	t0	t3	15	Actinomyces	Actinomyces_naeslundii	0.0473	0.0803	0.0303	0.0541	0.3586	1
TAU	t0 vs. t3	t0	t3	15	Actinomyces	Actinomyces_oris	0.0059	0.0123	0.0038	0.0083	0.2675	1
TAU	t0 vs. t3	t0	t3	15	Actinomyces	Actinomyces_viscosus	0.0005	0.0021	0.0005	0.0021	0.3343	1
TAU	t0 vs. t3	t0	t3	15	Actinomyces	Schaalia_odontolytica	0.2723	0.3292	0.3451	0.4185	0.5870	1
TAU	t0 vs. t3	t0	t3	15	Arcanobacterium	Arcanobacterium_phocae	0.0066	0.0257	0.0020	0.0077	0.5216	1
TAU	t0 vs. t3	t0	t3	15	Mobiluncus	Mobiluncus_curtisii	0.0002	0.0009	0.0016	0.0063	0.3343	1
TAU	t0 vs. t3	t0	t3	15	Alloscardovia	Alloscardovia_omnicolens	0.0009	0.0036	0.0019	0.0060	0.1728	1
TAU	t0 vs. t3	t0	t3	15	Bifidobacterium	Bifidobacterium_longum	0.0065	0.0251	0.0010	0.0038	0.3343	1
TAU	t0 vs. t3	t0	t3	15	Scardovia	Scardovia_inopinata	0.0005	0.0016	0.0002	0.0006	0.5048	1
TAU	t0 vs. t3	t0	t3	15	Scardovia	Scardovia_wiggsiae	0.0001	0.0002	0.0002	0.0005	0.2651	1
TAU	t0 vs. t3	t0	t3	15	Corynebacterium	Corynebacterium_diphtheriae	0.0043	0.0105	0.0012	0.0040	0.2546	1
TAU	t0 vs. t3	t0	t3	15	Corynebacterium	Corynebacterium_durum	0.0109	0.0248	0.0092	0.0211	0.8444	1
TAU	t0 vs. t3	t0	t3	15	Corynebacterium	Corynebacterium_matruchotii	0.0105	0.0251	0.0044	0.0082	0.3186	1
TAU	t0 vs. t3	t0	t3	15	Rothia	Rothia_aeria	0.0975	0.1362	0.0660	0.0658	0.3591	1
TAU	t0 vs. t3	t0	t3	15	Rothia	Rothia_mucilaginosa	3.9185	6.0747	1.7445	1.4110	0.1814	1
TAU	t0 vs. t3	t0	t3	15	Pseudopropionibacterium	Pseudopropionibacterium_propionicum	0.0000	0.0001	0.0001	0.0002	0.4860	1
TAU	t0 vs. t3	t0	t3	15	Atopobium	Lancefieldella_parvula	0.0342	0.0577	0.0276	0.0442	0.6797	1
TAU	t0 vs. t3	t0	t3	15	Cryptobacterium	Cryptobacterium_curtum	0.0002	0.0006	0.0001	0.0003	0.3343	1
TAU	t0 vs. t3	t0	t3	15	Slackia	Slackia_exigua	0.0005	0.0018	0.0018	0.0071	0.3343	1
TAU	t0 vs. t3	t0	t3	15	Bacteroides	Bacteroidaceae_bacterium	0.0070	0.0096	0.0054	0.0155	0.7461	1
TAU	t0 vs. t3	t0	t3	15	Phocaeicola	Phocaeicola_abscessus	0.0001	0.0004	0.0001	0.0005	0.3343	1
TAU	t0 vs. t3	t0	t3	15	F0058	Bacteroidetes_oral	0.0011	0.0034	0.0011	0.0038	0.9960	1
TAU	t0 vs. t3	t0	t3	15	Porphyromonas	Porphyromonas_asaccharolytica	0.0001	0.0003	0.0001	0.0004	0.3343	1
TAU	t0 vs. t3	t0	t3	15	Porphyromonas	Porphyromonas_catoniae	0.0258	0.0517	0.0238	0.0341	0.9037	1
TAU	t0 vs. t3	t0	t3	15	Porphyromonas	Porphyromonas_endodontalis	0.0157	0.0275	0.0067	0.0128	0.1721	1
TAU	t0 vs. t3	t0	t3	15	Porphyromonas	Porphyromonas_gingivalis	0.0015	0.0058	0.0003	0.0012	0.3343	1
TAU	t0 vs. t3	t0	t3	15	Porphyromonas	Porphyromonas_pasteri	0.9479	1.2828	1.4016	1.4101	0.2125	1

TAU	t0 vs. t3	t0	t3	15	Alloprevotella	Alloprevotella_rava	0.2480	0.6350	0.2879	0.6767	0.7667	1
TAU	t0 vs. t3	t0	t3	15	Alloprevotella	Alloprevotella_tannerae	0.1659	0.3927	0.1558	0.4075	0.7045	1
TAU	t0 vs. t3	t0	t3	15	Alloprevotella	Prevotellaceae_bacterium	0.6728	0.9676	0.5665	0.5328	0.5576	1
TAU	t0 vs. t3	t0	t3	15	Prevotella	Prevotella_aurantiaca	0.1986	0.5240	0.1132	0.2561	0.4882	1
TAU	t0 vs. t3	t0	t3	15	Prevotella	Prevotella_baroniae	0.0002	0.0006	0.0009	0.0035	0.4371	1
TAU	t0 vs. t3	t0	t3	15	Prevotella	Prevotella_dentalis	0.0018	0.0069	0.0011	0.0044	0.3343	1
TAU	t0 vs. t3	t0	t3	15	Prevotella	Prevotella_denticola	0.0032	0.0105	0.0079	0.0204	0.1389	1
TAU	t0 vs. t3	t0	t3	15	Prevotella	Prevotella_enoeca	0.0000	0.0001	0.0003	0.0012	0.3343	1
TAU	t0 vs. t3	t0	t3	15	Prevotella	Prevotella_fusca	0.0075	0.0275	0.0055	0.0148	0.6708	1
TAU	t0 vs. t3	t0	t3	15	Prevotella	Prevotella_histicola	0.2394	0.4235	0.2498	0.4655	0.9243	1
TAU	t0 vs. t3	t0	t3	15	Prevotella	Prevotella_intermedia	0.2272	0.3820	0.3190	0.5544	0.5720	1
TAU	t0 vs. t3	t0	t3	15	Prevotella	Prevotella_jejuni	0.2737	0.6903	0.0460	0.1648	0.2118	1
TAU	t0 vs. t3	t0	t3	15	Prevotella	Prevotella_loescheii	0.1969	0.3433	0.1156	0.2754	0.3360	1
TAU	t0 vs. t3	t0	t3	15	Prevotella	Prevotella_maculosa	0.0002	0.0008	0.0013	0.0036	0.2377	1
TAU	t0 vs. t3	t0	t3	15	Prevotella	Prevotella_melaninogenica	8.2966	6.9351	9.0842	6.5611	0.6916	1
TAU	t0 vs. t3	t0	t3	15	Prevotella	Prevotella_multiformis	0.0004	0.0014	0.0007	0.0028	0.3343	1
TAU	t0 vs. t3	t0	t3	15	Prevotella	Prevotella_nanceiensis	0.3818	0.7477	0.7716	1.1397	0.2111	1
TAU	t0 vs. t3	t0	t3	15	Prevotella	Prevotella_nigrescens	0.0329	0.0516	0.0445	0.1080	0.6458	1
TAU	t0 vs. t3	t0	t3	15	Prevotella	Prevotella_oralis	0.0000	0.0001	0.0004	0.0011	0.1649	1
TAU	t0 vs. t3	t0	t3	15	Prevotella	Prevotella_oulorum	0.0372	0.0707	0.0075	0.0214	0.1442	1
TAU	t0 vs. t3	t0	t3	15	Prevotella	Prevotella_pallens	0.3227	0.2613	0.7884	1.7856	0.2954	1
TAU	t0 vs. t3	t0	t3	15	Prevotella	Prevotella_pleuritidis	0.0104	0.0300	0.0032	0.0090	0.3443	1
TAU	t0 vs. t3	t0	t3	15	Prevotella	Prevotella_saccharolytica	0.0000	0.0002	0.0006	0.0016	0.2059	1
TAU	t0 vs. t3	t0	t3	15	Prevotella	Prevotella_salivae	0.7849	0.7584	0.8303	1.3968	0.8907	1
TAU	t0 vs. t3	t0	t3	15	Prevotella	Prevotella_shahii	0.0685	0.1471	0.0407	0.0976	0.4898	1
TAU	t0 vs. t3	t0	t3	15	Prevotella	Prevotella_veroralis	0.0006	0.0012	0.0016	0.0056	0.4696	1
TAU	t0 vs. t3	t0	t3	15	Rikenellaceae_RC9_gut_group	Bacteroidales_oral	0.0006	0.0024	0.0018	0.0059	0.2314	1
TAU	t0 vs. t3	t0	t3	15	Tannerella	Tannerella_forsythia	0.0224	0.0436	0.0037	0.0052	0.1263	1
TAU	t0 vs. t3	t0	t3	15	Capnocytophaga	Capnocytophaga_gingivalis	0.0640	0.0922	0.0490	0.0618	0.5354	1
TAU	t0 vs. t3	t0	t3	15	Capnocytophaga	Capnocytophaga_granulosa	0.0027	0.0062	0.0021	0.0055	0.7677	1
TAU	t0 vs. t3	t0	t3	15	Capnocytophaga	Capnocytophaga_leadbetteri	0.2540	0.5744	0.0586	0.0964	0.1380	1
TAU	t0 vs. t3	t0	t3	15	Capnocytophaga	Capnocytophaga_ochracea	0.1230	0.3285	0.0376	0.0912	0.1860	1
TAU	t0 vs. t3	t0	t3	15	Capnocytophaga	Capnocytophaga_sputigena	0.0921	0.2846	0.0800	0.1177	0.8343	1
TAU	t0 vs. t3	t0	t3	15	Bergeyella	Flavobacteriaceae_bacterium	0.0012	0.0040	0.0004	0.0011	0.4706	1
TAU	t0 vs. t3	t0	t3	15	Campylobacter	Campylobacter_concisus	0.0322	0.0308	0.0466	0.0634	0.3332	1
TAU	t0 vs. t3	t0	t3	15	Campylobacter	Campylobacter_showae	0.0014	0.0030	0.0019	0.0039	0.4812	1
TAU	t0 vs. t3	t0	t3	15	Desulfovibrio	Desulfovibrio_fairfieldensis	0.0001	0.0003	0.0004	0.0016	0.3343	1
TAU	t0 vs. t3	t0	t3	15	Solobacterium	Solobacterium_moorei	0.3329	0.3114	0.2214	0.2041	0.1665	1
TAU	t0 vs. t3	t0	t3	15	Globicatella	Aerococcaceae_bacterium	0.0013	0.0035	0.0109	0.0359	0.3253	1
TAU	t0 vs. t3	t0	t3	15	Granulicatella	Granulicatella_adiacens	0.0081	0.0191	0.0085	0.0257	0.9647	1
TAU	t0 vs. t3	t0	t3	15	Granulicatella	Granulicatella_elegans	0.0199	0.0233	0.0380	0.1106	0.5415	1
TAU	t0 vs. t3	t0	t3	15	Lactobacillus	Lactobacillus_acidophilus	0.0000	0.0000	0.0001	0.0004	0.3697	1
TAU	t0 vs. t3	t0	t3	15	Lactobacillus	Lactobacillus_amylovorus	0.0010	0.0040	0.0006	0.0022	0.3343	1
TAU	t0 vs. t3	t0	t3	15	Lactobacillus	Lactobacillus_helveticus	0.0031	0.0119	0.0020	0.0078	0.3343	1
TAU	t0 vs. t3	t0	t3	15	Lactobacillus	Lactobacillus_reuteri	0.0009	0.0034	0.0011	0.0042	0.3343	1
TAU	t0 vs. t3	t0	t3	15	Lactobacillus	Lactobacillus_sakei	0.0026	0.0100	0.0062	0.0161	0.0726	1
TAU	t0 vs. t3	t0	t3	15	Lactobacillus	Lactobacillus_vaginalis	0.0002	0.0007	0.0008	0.0032	0.3343	1
TAU	t0 vs. t3	t0	t3	15	Streptococcus	Coregonus_clupeaformis	0.0002	0.0008	0.0013	0.0027	0.1756	1
TAU	t0 vs. t3	t0	t3	15	Streptococcus	Streptococcus_anginosus	0.0405	0.1355	0.0171	0.0458	0.3460	1
TAU	t0 vs. t3	t0	t3	15	Streptococcus	Streptococcus_australis	0.0036	0.0068	0.0152	0.0223	0.0480	1

TAU	t0 vs. t3	t0	t3	15	Streptococcus	Streptococcus_cristatus	0.0215	0.0320	0.0184	0.0259	0.6880	1
TAU	t0 vs. t3	t0	t3	15	Streptococcus	Streptococcus_gordonii	0.0428	0.0506	0.0281	0.0285	0.2830	1
TAU	t0 vs. t3	t0	t3	15	Streptococcus	Streptococcus_infantis	0.0287	0.0314	0.0329	0.0319	0.6282	1
TAU	t0 vs. t3	t0	t3	15	Streptococcus	Streptococcus_mitis	0.0164	0.0585	0.0094	0.0140	0.6174	1
TAU	t0 vs. t3	t0	t3	15	Streptococcus	Streptococcus_mutans	0.0001	0.0004	0.0001	0.0003	0.6413	1
TAU	t0 vs. t3	t0	t3	15	Streptococcus	Streptococcus_oralis	0.0030	0.0052	0.0087	0.0186	0.2710	1
TAU	t0 vs. t3	t0	t3	15	Streptococcus	Streptococcus_parasanguinis	1.1674	0.8114	2.2383	4.5769	0.3283	1
TAU	t0 vs. t3	t0	t3	15	Streptococcus	Streptococcus_pneumoniae	0.7073	0.8546	0.7488	1.1966	0.9061	1
TAU	t0 vs. t3	t0	t3	15	Streptococcus	Streptococcus_salivarius	6.2873	6.3219	8.8163	10.2213	0.3818	1
TAU	t0 vs. t3	t0	t3	15	Streptococcus	Streptococcus_sanguinis	0.0639	0.1132	0.1243	0.1783	0.1298	1
TAU	t0 vs. t3	t0	t3	15	Streptococcus	Streptococcus_suis	0.6711	1.5564	0.3964	0.4694	0.3755	1
TAU	t0 vs. t3	t0	t3	15	RF39	Firmicutes_oral	0.0011	0.0042	0.0021	0.0057	0.2492	1
TAU	t0 vs. t3	t0	t3	15	Gemella	Gemella_haemolysans	1.5002	1.3369	1.5690	1.2345	0.8028	1
TAU	t0 vs. t3	t0	t3	15	Gemella	Gemella_morbillorum	0.0184	0.0212	0.0144	0.0202	0.5651	1
TAU	t0 vs. t3	t0	t3	15	Gemella	Gemella_sanguinis	0.0609	0.0585	0.1241	0.1283	0.0403	1
TAU	t0 vs. t3	t0	t3	15	Staphylococcus	Staphylococcus_aureus	0.0323	0.1236	0.0009	0.0036	0.3442	1
TAU	t0 vs. t3	t0	t3	15	Clostridia_UCG-014	Clostridiales_bacterium	0.3496	0.5315	0.1206	0.0929	0.1337	1
TAU	t0 vs. t3	t0	t3	15	Defluviitaleaceae_UCG-011	Lachnospiraceae_bacterium	0.0032	0.0069	0.0043	0.0114	0.7318	1
TAU	t0 vs. t3	t0	t3	15	Catonella	Catonella_genomosp.	0.0015	0.0044	0.0012	0.0031	0.6740	1
TAU	t0 vs. t3	t0	t3	15	Catonella	Catonella_morbi	0.0610	0.0823	0.0500	0.0616	0.4203	1
TAU	t0 vs. t3	t0	t3	15	Howardella	Howardella_ureilytica	0.0002	0.0010	0.0029	0.0114	0.3343	1
TAU	t0 vs. t3	t0	t3	15	Johnsonella	Firmicutes_oral	0.0013	0.0035	0.0003	0.0009	0.2061	1
TAU	t0 vs. t3	t0	t3	15	Johnsonella	Johnsonella_ignava	0.0002	0.0008	0.0001	0.0002	0.5476	1
TAU	t0 vs. t3	t0	t3	15	Johnsonella	Lachnospiraceae_bacterium	0.0056	0.0113	0.0023	0.0053	0.2532	1
TAU	t0 vs. t3	t0	t3	15	Lachnoanaerobaculum	Lachnoanaerobaculum_umeaense	0.2359	0.3408	0.1941	0.2509	0.6460	1
TAU	t0 vs. t3	t0	t3	15	Shuttleworthia	Shuttleworthia_satelles	0.0008	0.0032	0.0078	0.0273	0.2831	1
TAU	t0 vs. t3	t0	t3	15	Stomatobaculum	Stomatobaculum_longum	0.0961	0.1172	0.1187	0.2103	0.7180	1
TAU	t0 vs. t3	t0	t3	15	[Eubacterium]_brachy_group	[Eubacterium]_brachy	0.0029	0.0102	0.0012	0.0040	0.5519	1
TAU	t0 vs. t3	t0	t3	15	[Eubacterium]_saphenum_group	Eubacterium_saphenum	0.0000	0.0001	0.0041	0.0157	0.3343	1
TAU	t0 vs. t3	t0	t3	15	Amnipila	Peptostreptococcaceae_bacterium	0.0043	0.0166	0.0049	0.0190	0.3343	1
TAU	t0 vs. t3	t0	t3	15	Family_XIII_UCG-001	Peptostreptococcaceae_bacterium	0.0077	0.0295	0.0136	0.0401	0.5938	1
TAU	t0 vs. t3	t0	t3	15	[Eubacterium]_yurii_group	[Eubacterium]_yurii	0.0335	0.1151	0.0212	0.0425	0.5895	1
TAU	t0 vs. t3	t0	t3	15	Filifactor	Filifactor_alocis	0.0158	0.0349	0.0058	0.0163	0.2091	1
TAU	t0 vs. t3	t0	t3	15	Peptoanaerobacter	Peptoanaerobacter_stomatis	0.0005	0.0021	0.0002	0.0006	0.3343	1
TAU	t0 vs. t3	t0	t3	15	Parvimonas	Parvimonas_micra	0.0016	0.0026	0.0056	0.0110	0.1711	1
TAU	t0 vs. t3	t0	t3	15	Selenomonas	Selenomonas_flueggei	0.0026	0.0069	0.0017	0.0067	0.7404	1
TAU	t0 vs. t3	t0	t3	15	Selenomonas	Selenomonas_infelix	0.0101	0.0269	0.0013	0.0051	0.2067	1
TAU	t0 vs. t3	t0	t3	15	Selenomonas	Selenomonas_massiliensis	0.0090	0.0200	0.0155	0.0431	0.5216	1
TAU	t0 vs. t3	t0	t3	15	Selenomonas	Selenomonas_noxia	0.0166	0.0282	0.0270	0.0696	0.5088	1
TAU	t0 vs. t3	t0	t3	15	Selenomonas	Selenomonas_sputigena	0.0517	0.1000	0.0306	0.0723	0.2162	1
TAU	t0 vs. t3	t0	t3	15	Dialister	Dialister_invisus	0.2969	1.0356	0.1893	0.6707	0.7441	1
TAU	t0 vs. t3	t0	t3	15	Dialister	Dialister_micraerophilus	0.0004	0.0015	0.0060	0.0232	0.3343	1
TAU	t0 vs. t3	t0	t3	15	Dialister	Dialister_pneumosintes	0.0031	0.0095	0.0327	0.1265	0.3455	1
TAU	t0 vs. t3	t0	t3	15	Megasphaera	Megasphaera_micronuciformis	0.3705	0.5965	0.3897	1.0443	0.9192	1
TAU	t0 vs. t3	t0	t3	15	Veillonella	Veillonella_atypica	1.1276	1.3859	1.0351	1.6865	0.7886	1
TAU	t0 vs. t3	t0	t3	15	Veillonella	Veillonella_parvula	3.3435	2.6704	2.6414	2.2854	0.4296	1
TAU	t0 vs. t3	t0	t3	15	Veillonella	Veillonella_rogosae	0.0623	0.1668	0.0669	0.2554	0.9350	1
TAU	t0 vs. t3	t0	t3	15	Fusobacterium	Fusobacterium_nucleatum	0.6693	0.5362	0.8566	0.8203	0.3490	1
TAU	t0 vs. t3	t0	t3	15	Fusobacterium	Fusobacterium_periodonticum	1.2702	0.8689	1.6619	1.3008	0.2304	1
TAU	t0 vs. t3	t0	t3	15	Leptotrichia	Leptotrichia_buccalis	0.2297	0.3838	0.1755	0.2814	0.6941	1

TAU	t0 vs. t3	t0	t3	15	Leptotrichia	Leptotrichia_goodfellowii	0.0020	0.0068	0.0039	0.0102	0.5753	1
TAU	t0 vs. t3	t0	t3	15	Leptotrichia	Leptotrichia_hofstadii	0.0053	0.0092	0.0058	0.0110	0.8842	1
TAU	t0 vs. t3	t0	t3	15	Leptotrichia	Leptotrichia_hongkongensis	0.0007	0.0018	0.0016	0.0035	0.2445	1
TAU	t0 vs. t3	t0	t3	15	Leptotrichia	Leptotrichia_shahii	0.0282	0.0717	0.0181	0.0680	0.6914	1
TAU	t0 vs. t3	t0	t3	15	Leptotrichia	Leptotrichia_trevisanii	0.0002	0.0005	0.0002	0.0006	0.6922	1
TAU	t0 vs. t3	t0	t3	15	Leptotrichia	Leptotrichia_wadei	0.2717	0.5027	0.0946	0.1508	0.2227	1
TAU	t0 vs. t3	t0	t3	15	Absconditabacteriales_(SR1)	SR1_bacterium	0.0082	0.0180	0.0075	0.0133	0.8287	1
TAU	t0 vs. t3	t0	t3	15	Gracilibacteria	Gracilibacteria_bacterium	0.0001	0.0004	0.0002	0.0009	0.6361	1
TAU	t0 vs. t3	t0	t3	15	JGI_0000069-P22	Gracilibacteria_bacterium	0.0001	0.0004	0.0005	0.0013	0.1206	1
TAU	t0 vs. t3	t0	t3	15	Candidatus_Saccharimonas	TM7_phylum	0.1080	0.1387	0.0821	0.0805	0.4380	1
TAU	t0 vs. t3	t0	t3	15	Saccharimonadaceae	TM7_bacterium	0.0047	0.0063	0.0047	0.0103	0.9943	1
TAU	t0 vs. t3	t0	t3	15	Saccharimonadaceae	TM7_phylum	0.0006	0.0018	0.0004	0.0010	0.3343	1
TAU	t0 vs. t3	t0	t3	15	TM7a	candidate_division	0.0001	0.0005	0.0003	0.0004	0.2129	1
TAU	t0 vs. t3	t0	t3	15	TM7x	Candidatus_Saccharibacteria	0.1484	0.2554	0.1000	0.1626	0.3675	1
TAU	t0 vs. t3	t0	t3	15	TM7x	TM7_phylum	0.2701	0.3359	0.1980	0.3227	0.1668	1
TAU	t0 vs. t3	t0	t3	15	Saccharimonadales	Candidatus_Saccharibacteria	0.0009	0.0028	0.0004	0.0014	0.2012	1
TAU	t0 vs. t3	t0	t3	15	Saccharimonadales	TM7_phylum	0.2459	0.3330	0.0767	0.1029	0.0231	1
TAU	t0 vs. t3	t0	t3	15	Shewanella	Shewanella_baltica	0.0004	0.0010	0.0000	0.0002	0.2016	1
TAU	t0 vs. t3	t0	t3	15	Shewanella	Shewanella_putrefaciens	0.0035	0.0064	0.0028	0.0054	0.7367	1
TAU	t0 vs. t3	t0	t3	15	Lautropia	Lautropia_mirabilis	0.0862	0.2064	0.0722	0.1072	0.8155	1
TAU	t0 vs. t3	t0	t3	15	Brachymonas	Brachymonas_denitrificans	0.0028	0.0074	0.0010	0.0018	0.3836	1
TAU	t0 vs. t3	t0	t3	15	Eikenella	Eikenella_corrodens	0.0000	0.0001	0.0006	0.0013	0.1002	1
TAU	t0 vs. t3	t0	t3	15	Kingella	Kingella_oralis	0.0014	0.0046	0.0091	0.0324	0.3018	1
TAU	t0 vs. t3	t0	t3	15	Neisseria	Neisseria_bacilliformis	0.0015	0.0047	0.0037	0.0122	0.5478	1
TAU	t0 vs. t3	t0	t3	15	Neisseria	Neisseria_elongata	0.0168	0.0241	0.0174	0.0262	0.9452	1
TAU	t0 vs. t3	t0	t3	15	Neisseria	Neisseria_meningitidis	7.6120	4.4722	7.1833	4.6507	0.6661	1
TAU	t0 vs. t3	t0	t3	15	Neisseria	Neisseria_oralis	0.0093	0.0186	0.0443	0.1056	0.2223	1
TAU	t0 vs. t3	t0	t3	15	Neisseria	Neisseria_perflava	0.0023	0.0038	0.0033	0.0063	0.6360	1
TAU	t0 vs. t3	t0	t3	15	Simonsiella	Simonsiella_muelleri	0.0067	0.0178	0.0005	0.0014	0.1763	1
TAU	t0 vs. t3	t0	t3	15	Cardiobacterium	Cardiobacterium_hominis	0.0042	0.0059	0.0054	0.0089	0.5551	1
TAU	t0 vs. t3	t0	t3	15	Cardiobacterium	Cardiobacterium_valvarum	0.0072	0.0089	0.0045	0.0062	0.1662	1
TAU	t0 vs. t3	t0	t3	15	Actinobacillus	Actinobacillus_pleuropneumoniae	0.0199	0.0390	0.0264	0.0532	0.7062	1
TAU	t0 vs. t3	t0	t3	15	Actinobacillus	Haemophilus_parahaemolyticus	0.0138	0.0325	0.0034	0.0082	0.2383	1
TAU	t0 vs. t3	t0	t3	15	Aggregatibacter	Aggregatibacter_aphrophilus	0.0713	0.0941	0.1091	0.1955	0.4836	1
TAU	t0 vs. t3	t0	t3	15	Aggregatibacter	Aggregatibacter_segnis	0.0283	0.0359	0.0140	0.0248	0.1724	1
TAU	t0 vs. t3	t0	t3	15	Haemophilus	[Haemophilus]_ducreyi	0.0020	0.0055	0.0030	0.0093	0.4081	1
TAU	t0 vs. t3	t0	t3	15	Haemophilus	Haemophilus_haemolyticus	0.0572	0.0794	0.0885	0.1138	0.1073	1
TAU	t0 vs. t3	t0	t3	15	Haemophilus	Haemophilus_influenzae	0.0431	0.0521	0.0858	0.1027	0.0510	1
TAU	t0 vs. t3	t0	t3	15	Haemophilus	Haemophilus_parainfluenzae	0.9180	0.7281	1.1573	0.7069	0.0751	1
TAU	t0 vs. t3	t0	t3	15	Haemophilus	Haemophilus_pittmaniae	0.5078	0.5680	0.5331	0.4289	0.8560	1
TAU	t0 vs. t3	t0	t3	15	Haemophilus	Haemophilus_sputorum	0.0235	0.0399	0.0421	0.0612	0.3410	1
TAU	t0 vs. t3	t0	t3	15	Mannheimia	Mannheimia_haemolytica	0.0199	0.0304	0.0400	0.0855	0.3479	1
TAU	t0 vs. t3	t0	t3	15	Pasteurella	Pasteurella_multocida	0.4493	0.5113	0.8064	0.7533	0.0896	1
TAU	t0 vs. t3	t0	t3	15	Rodentibacter	Pasteurellaceae_bacterium	0.0045	0.0119	0.0292	0.0967	0.3474	1
TAU	t0 vs. t3	t0	t3	15	Acinetobacter	Acinetobacter_johnsonii	0.0005	0.0017	0.0007	0.0011	0.8180	1
TAU	t0 vs. t3	t0	t3	15	Treponema	Treponema_denticola	0.0005	0.0020	0.0007	0.0027	0.3932	1
TAU	t0 vs. t3	t0	t3	15	Treponema	Treponema_maltophilum	0.0001	0.0005	0.0004	0.0015	0.3343	1
TAU	t0 vs. t3	t0	t3	15	Treponema	Treponema_medium	0.0028	0.0069	0.0034	0.0089	0.6835	1
TAU	t0 vs. t3	t0	t3	15	Treponema	Treponema_refringens	0.0000	0.0001	0.0003	0.0011	0.3098	1
TAU	t0 vs. t3	t0	t3	15	Fretibacterium	Synergistales_bacterium	0.0005	0.0016	0.0056	0.0208	0.3310	1

TAU	t0 vs. t6	t0	t6	14	Actinomyces	Actinomyces_dentalis	0.0000	0.0001	0.0009	0.0025	0.2431	1
TAU	t0 vs. t6	t0	t6	14	Actinomyces	Actinomyces_graevenitzii	1.0179	1.3267	1.0914	1.3939	0.8037	1
TAU	t0 vs. t6	t0	t6	14	Actinomyces	Actinomyces_naeslundii	0.0507	0.0822	0.0355	0.0855	0.4756	1
TAU	t0 vs. t6	t0	t6	14	Actinomyces	Actinomyces_oris	0.0063	0.0127	0.0016	0.0055	0.0966	1
TAU	t0 vs. t6	t0	t6	14	Actinomyces	Actinomyces_viscosus	0.0006	0.0022	0.0004	0.0011	0.7554	1
TAU	t0 vs. t6	t0	t6	14	Actinomyces	Schaalia_odontolytica	0.2674	0.3411	0.3805	0.4124	0.4001	1
TAU	t0 vs. t6	t0	t6	14	Alloscardovia	Alloscardovia_omnicolens	0.0010	0.0037	0.0128	0.0272	0.1215	1
TAU	t0 vs. t6	t0	t6	14	Bifidobacterium	Bifidobacterium_longum	0.0069	0.0260	0.0043	0.0162	0.3356	1
TAU	t0 vs. t6	t0	t6	14	Scardovia	Scardovia_inopinata	0.0005	0.0017	0.0001	0.0005	0.2699	1
TAU	t0 vs. t6	t0	t6	14	Scardovia	Scardovia_wiggsiae	0.0001	0.0002	0.0000	0.0001	0.5022	1
TAU	t0 vs. t6	t0	t6	14	Corynebacterium	Corynebacterium_diphtheriae	0.0046	0.0108	0.0006	0.0019	0.1909	1
TAU	t0 vs. t6	t0	t6	14	Corynebacterium	Corynebacterium_durum	0.0117	0.0255	0.0169	0.0485	0.7128	1
TAU	t0 vs. t6	t0	t6	14	Corynebacterium	Corynebacterium_matruchotii	0.0113	0.0259	0.0025	0.0053	0.2377	1
TAU	t0 vs. t6	t0	t6	14	Rothia	Rothia_aeria	0.1038	0.1391	0.1222	0.1884	0.7396	1
TAU	t0 vs. t6	t0	t6	14	Rothia	Rothia_mucilaginosa	4.1697	6.2227	3.8246	3.4970	0.8424	1
TAU	t0 vs. t6	t0	t6	14	Pseudopropionibacterium	Pseudopropionibacterium_propionicum	0.0000	0.0001	0.0002	0.0006	0.3356	1
TAU	t0 vs. t6	t0	t6	14	Atopobium	Lancefieldella_parvula	0.0279	0.0542	0.0210	0.0276	0.5149	1
TAU	t0 vs. t6	t0	t6	14	Cryptobacterium	Cryptobacterium_curtum	0.0002	0.0006	0.0008	0.0031	0.3356	1
TAU	t0 vs. t6	t0	t6	14	Bacteroides	Bacteroidaceae_bacterium	0.0075	0.0097	0.0004	0.0011	0.0201	1
TAU	t0 vs. t6	t0	t6	14	F0058	Bacteroidetes_oral	0.0011	0.0035	0.0045	0.0091	0.2111	1
TAU	t0 vs. t6	t0	t6	14	Porphyromonas	Porphyromonas_catoniae	0.0271	0.0533	0.0174	0.0295	0.5804	1
TAU	t0 vs. t6	t0	t6	14	Porphyromonas	Porphyromonas_endodontalis	0.0166	0.0283	0.0076	0.0198	0.0506	1
TAU	t0 vs. t6	t0	t6	14	Porphyromonas	Porphyromonas_gingivalis	0.0016	0.0060	0.0002	0.0006	0.3892	1
TAU	t0 vs. t6	t0	t6	14	Porphyromonas	Porphyromonas_pasteri	1.0114	1.3065	1.2826	0.9950	0.3613	1
TAU	t0 vs. t6	t0	t6	14	Alloprevotella	Alloprevotella_rava	0.0959	0.2459	0.1972	0.5037	0.4669	1
TAU	t0 vs. t6	t0	t6	14	Alloprevotella	Alloprevotella_tannerae	0.1737	0.4063	0.0871	0.2653	0.2205	1
TAU	t0 vs. t6	t0	t6	14	Alloprevotella	Prevotellaceae_bacterium	0.7019	0.9973	0.8402	0.9999	0.6888	1
TAU	t0 vs. t6	t0	t6	14	Prevotella	Prevotella_aurantiaca	0.2128	0.5408	0.1461	0.4323	0.0742	1
TAU	t0 vs. t6	t0	t6	14	Prevotella	Prevotella_baroniae	0.0002	0.0007	0.0000	0.0001	0.2386	1
TAU	t0 vs. t6	t0	t6	14	Prevotella	Prevotella_buccae	0.0014	0.0048	0.0011	0.0027	0.6624	1
TAU	t0 vs. t6	t0	t6	14	Prevotella	Prevotella_denticola	0.0034	0.0108	0.0045	0.0094	0.3830	1
TAU	t0 vs. t6	t0	t6	14	Prevotella	Prevotella_enoeca	0.0000	0.0001	0.0004	0.0014	0.3859	1
TAU	t0 vs. t6	t0	t6	14	Prevotella	Prevotella_fusca	0.0080	0.0285	0.0010	0.0030	0.3845	1
TAU	t0 vs. t6	t0	t6	14	Prevotella	Prevotella_histicola	0.2311	0.4383	0.5654	1.2562	0.2853	1
TAU	t0 vs. t6	t0	t6	14	Prevotella	Prevotella_intermedia	0.2283	0.3964	0.1814	0.2459	0.4911	1
TAU	t0 vs. t6	t0	t6	14	Prevotella	Prevotella_jejuni	0.2922	0.7126	0.6865	1.9642	0.4140	1
TAU	t0 vs. t6	t0	t6	14	Prevotella	Prevotella_loescheii	0.2055	0.3546	0.4095	0.6987	0.1731	1
TAU	t0 vs. t6	t0	t6	14	Prevotella	Prevotella_maculosa	0.0002	0.0009	0.0003	0.0010	0.8956	1
TAU	t0 vs. t6	t0	t6	14	Prevotella	Prevotella_melaninogenica	7.1541	5.5413	7.7778	5.8302	0.6783	1
TAU	t0 vs. t6	t0	t6	14	Prevotella	Prevotella_nanceiensis	0.4043	0.7706	0.7252	1.1135	0.3370	1
TAU	t0 vs. t6	t0	t6	14	Prevotella	Prevotella_nigrescens	0.0330	0.0536	0.0217	0.0317	0.4094	1
TAU	t0 vs. t6	t0	t6	14	Prevotella	Prevotella_oralis	0.0000	0.0001	0.0008	0.0020	0.1661	1
TAU	t0 vs. t6	t0	t6	14	Prevotella	Prevotella_oulorum	0.0385	0.0732	0.0788	0.1526	0.1691	1
TAU	t0 vs. t6	t0	t6	14	Prevotella	Prevotella_pallens	0.3030	0.2594	0.5643	0.7183	0.1475	1
TAU	t0 vs. t6	t0	t6	14	Prevotella	Prevotella_pleuritidis	0.0112	0.0309	0.0012	0.0043	0.2292	1
TAU	t0 vs. t6	t0	t6	14	Prevotella	Prevotella_saccharolytica	0.0001	0.0002	0.0011	0.0029	0.2088	1
TAU	t0 vs. t6	t0	t6	14	Prevotella	Prevotella_salivae	0.7021	0.7131	0.8292	1.0827	0.4233	1
TAU	t0 vs. t6	t0	t6	14	Prevotella	Prevotella_shahii	0.0725	0.1518	0.1639	0.2642	0.0838	1
TAU	t0 vs. t6	t0	t6	14	Prevotella	Prevotella_veroralis	0.0004	0.0011	0.0005	0.0014	0.8798	1
TAU	t0 vs. t6	t0	t6	14	Rikenellaceae_RC9_gut_group	Bacteroidales_oral	0.0007	0.0025	0.0001	0.0002	0.3826	1

TAU	t0 vs. t6	t0	t6	14	Tannerella	Tannerella_forsythia	0.0229	0.0452	0.0302	0.0532	0.6495	1
TAU	t0 vs. t6	t0	t6	14	Capnocytophaga	Capnocytophaga_gingivalis	0.0674	0.0946	0.0622	0.0535	0.8625	1
TAU	t0 vs. t6	t0	t6	14	Capnocytophaga	Capnocytophaga_granulosa	0.0013	0.0030	0.0032	0.0071	0.3569	1
TAU	t0 vs. t6	t0	t6	14	Capnocytophaga	Capnocytophaga_leadbetteri	0.2721	0.5916	0.1427	0.2444	0.3098	1
TAU	t0 vs. t6	t0	t6	14	Capnocytophaga	Capnocytophaga_ochracea	0.1318	0.3391	0.0697	0.0924	0.5009	1
TAU	t0 vs. t6	t0	t6	14	Capnocytophaga	Capnocytophaga_sputigena	0.0986	0.2942	0.0902	0.1654	0.8870	1
TAU	t0 vs. t6	t0	t6	14	Bergeyella	Flavobacteriaceae_bacterium	0.0013	0.0042	0.0015	0.0033	0.9194	1
TAU	t0 vs. t6	t0	t6	14	Chryseobacterium	Chryseobacterium_reticulitermitis	0.0005	0.0013	0.0004	0.0016	0.7244	1
TAU	t0 vs. t6	t0	t6	14	Campylobacter	Campylobacter_concisus	0.0306	0.0313	0.0824	0.1940	0.2895	1
TAU	t0 vs. t6	t0	t6	14	Campylobacter	Campylobacter_showae	0.0012	0.0031	0.0046	0.0063	0.0758	1
TAU	t0 vs. t6	t0	t6	14	Solobacterium	Solobacterium_moorei	0.2993	0.2936	0.2692	0.4013	0.6926	1
TAU	t0 vs. t6	t0	t6	14	Globicatella	Aerococcaceae_bacterium	0.0014	0.0037	0.0035	0.0101	0.4819	1
TAU	t0 vs. t6	t0	t6	14	Granulicatella	Granulicatella_adiacens	0.0087	0.0197	0.0011	0.0027	0.1807	1
TAU	t0 vs. t6	t0	t6	14	Granulicatella	Granulicatella_elegans	0.0194	0.0241	0.0403	0.1063	0.4285	1
TAU	t0 vs. t6	t0	t6	14	Lactobacillus	Lactobacillus_acidophilus	0.0000	0.0000	0.0021	0.0079	0.3356	1
TAU	t0 vs. t6	t0	t6	14	Lactobacillus	Lactobacillus_amylovorus	0.0011	0.0042	0.0262	0.0978	0.3356	1
TAU	t0 vs. t6	t0	t6	14	Lactobacillus	Lactobacillus_crispatus	0.0005	0.0019	0.0049	0.0185	0.3356	1
TAU	t0 vs. t6	t0	t6	14	Lactobacillus	Lactobacillus_fermentum	0.0023	0.0087	0.0284	0.1061	0.3356	1
TAU	t0 vs. t6	t0	t6	14	Lactobacillus	Lactobacillus_gasseri	0.0000	0.0002	0.0009	0.0033	0.3356	1
TAU	t0 vs. t6	t0	t6	14	Lactobacillus	Lactobacillus_helveticus	0.0033	0.0123	0.0767	0.2851	0.3321	1
TAU	t0 vs. t6	t0	t6	14	Lactobacillus	Lactobacillus_reuteri	0.0009	0.0035	0.0226	0.0844	0.3356	1
TAU	t0 vs. t6	t0	t6	14	Lactobacillus	Lactobacillus_sakei	0.0028	0.0104	0.0003	0.0013	0.4029	1
TAU	t0 vs. t6	t0	t6	14	Lactobacillus	Lactobacillus_vaginalis	0.0002	0.0007	0.0152	0.0568	0.3356	1
TAU	t0 vs. t6	t0	t6	14	Lactococcus	Lactococcus_lactis	0.0131	0.0488	0.0007	0.0025	0.3606	1
TAU	t0 vs. t6	t0	t6	14	Streptococcus	Streptococcus_anginosus	0.0434	0.1401	0.0057	0.0128	0.2963	1
TAU	t0 vs. t6	t0	t6	14	Streptococcus	Streptococcus_australis	0.0039	0.0070	0.0086	0.0099	0.0719	1
TAU	t0 vs. t6	t0	t6	14	Streptococcus	Streptococcus_cristatus	0.0212	0.0332	0.0084	0.0135	0.2001	1
TAU	t0 vs. t6	t0	t6	14	Streptococcus	Streptococcus_gordonii	0.0413	0.0522	0.0229	0.0308	0.2326	1
TAU	t0 vs. t6	t0	t6	14	Streptococcus	Streptococcus_infantis	0.0308	0.0315	0.0325	0.0291	0.8730	1
TAU	t0 vs. t6	t0	t6	14	Streptococcus	Streptococcus_mitis	0.0176	0.0605	0.0061	0.0107	0.4284	1
TAU	t0 vs. t6	t0	t6	14	Streptococcus	Streptococcus_oralis	0.0029	0.0054	0.0092	0.0148	0.1472	1
TAU	t0 vs. t6	t0	t6	14	Streptococcus	Streptococcus_parasanguinis	1.1967	0.8337	0.8225	0.7793	0.0456	1
TAU	t0 vs. t6	t0	t6	14	Streptococcus	Streptococcus_pneumoniae	0.7399	0.8772	0.3300	0.2997	0.0429	1
TAU	t0 vs. t6	t0	t6	14	Streptococcus	Streptococcus_salivarius	5.6856	6.0985	3.6240	3.5179	0.1766	1
TAU	t0 vs. t6	t0	t6	14	Streptococcus	Streptococcus_sanguinis	0.0685	0.1160	0.1676	0.4113	0.3223	1
TAU	t0 vs. t6	t0	t6	14	Streptococcus	Streptococcus_suis	0.7053	1.6093	0.3750	0.3406	0.4676	1
TAU	t0 vs. t6	t0	t6	14	RF39	Firmicutes_oral	0.0012	0.0044	0.0037	0.0120	0.2352	1
TAU	t0 vs. t6	t0	t6	14	Gemella	Gemella_haemolysans	1.5904	1.3392	1.6337	0.8754	0.8829	1
TAU	t0 vs. t6	t0	t6	14	Gemella	Gemella_morbillorum	0.0197	0.0213	0.0125	0.0179	0.0272	1
TAU	t0 vs. t6	t0	t6	14	Gemella	Gemella_sanguinis	0.0650	0.0584	0.1122	0.0867	0.0561	1
TAU	t0 vs. t6	t0	t6	14	Staphylococcus	Staphylococcus_aureus	0.0346	0.1280	0.0001	0.0003	0.3316	1
TAU	t0 vs. t6	t0	t6	14	Clostridia_UCG-014	Clostridiales_bacterium	0.3477	0.5515	0.3902	0.5506	0.8403	1
TAU	t0 vs. t6	t0	t6	14	Defluviitaleaceae_UCG-011	Lachnospiraceae_bacterium	0.0030	0.0072	0.0066	0.0243	0.4847	1
TAU	t0 vs. t6	t0	t6	14	Catonella	Catonella_morbi	0.0647	0.0841	0.0480	0.0869	0.4040	1
TAU	t0 vs. t6	t0	t6	14	Howardella	Howardella_ureilytica	0.0003	0.0010	0.0028	0.0095	0.2939	1
TAU	t0 vs. t6	t0	t6	14	Johnsonella	Firmicutes_oral	0.0014	0.0036	0.0002	0.0007	0.2599	1
TAU	t0 vs. t6	t0	t6	14	Johnsonella	Johnsonella_ignava	0.0002	0.0009	0.0001	0.0003	0.5657	1
TAU	t0 vs. t6	t0	t6	14	Johnsonella	Lachnospiraceae_bacterium	0.0057	0.0117	0.0106	0.0387	0.5556	1
TAU	t0 vs. t6	t0	t6	14	Lachnoanaerobaculum	Lachnoanaerobaculum_umeaense	0.2481	0.3503	0.1319	0.1106	0.2258	1
TAU	t0 vs. t6	t0	t6	14	Shuttleworthia	Shuttleworthia_satelles	0.0009	0.0033	0.0013	0.0041	0.1668	1

TAU	t0 vs. t6	t0	t6	14	Stomatobaculum	Stomatobaculum_longum	0.0903	0.1194	0.0529	0.0545	0.2267	1
TAU	t0 vs. t6	t0	t6	14	[Eubacterium]_brachy_group	[Eubacterium]_brachy	0.0031	0.0105	0.0003	0.0007	0.3334	1
TAU	t0 vs. t6	t0	t6	14	Amnipila	Peptostreptococcaceae_bacterium	0.0046	0.0172	0.0035	0.0125	0.4062	1
TAU	t0 vs. t6	t0	t6	14	Family_XIII_UCG-001	Peptostreptococcaceae_bacterium	0.0082	0.0305	0.0015	0.0055	0.3313	1
TAU	t0 vs. t6	t0	t6	14	[Eubacterium]_yurii_group	[Eubacterium]_yurii	0.0359	0.1191	0.0143	0.0255	0.5276	1
TAU	t0 vs. t6	t0	t6	14	Filifactor	Filifactor_alocis	0.0169	0.0359	0.0036	0.0128	0.1262	1
TAU	t0 vs. t6	t0	t6	14	Parvimonas	Parvimonas_micra	0.0017	0.0027	0.0033	0.0068	0.4327	1
TAU	t0 vs. t6	t0	t6	14	Selenomonas	Selenomonas_flueggei	0.0028	0.0071	0.0886	0.3317	0.3439	1
TAU	t0 vs. t6	t0	t6	14	Selenomonas	Selenomonas_infelix	0.0109	0.0278	0.0007	0.0027	0.1582	1
TAU	t0 vs. t6	t0	t6	14	Selenomonas	Selenomonas_massiliensis	0.0066	0.0184	0.0099	0.0223	0.6959	1
TAU	t0 vs. t6	t0	t6	14	Selenomonas	Selenomonas_noxia	0.0178	0.0289	0.0103	0.0229	0.3642	1
TAU	t0 vs. t6	t0	t6	14	Selenomonas	Selenomonas_sputigena	0.0552	0.1028	0.0378	0.0875	0.2289	1
TAU	t0 vs. t6	t0	t6	14	Anaeroglobus	Anaeroglobus_geminatus	0.0003	0.0011	0.0001	0.0005	0.6000	1
TAU	t0 vs. t6	t0	t6	14	Dialister	Dialister_invisus	0.3181	1.0713	0.0460	0.1042	0.3195	1
TAU	t0 vs. t6	t0	t6	14	Dialister	Dialister_micraerophilus	0.0004	0.0016	0.0010	0.0025	0.3101	1
TAU	t0 vs. t6	t0	t6	14	Dialister	Dialister_pneumosintes	0.0033	0.0098	0.0026	0.0078	0.8210	1
TAU	t0 vs. t6	t0	t6	14	Megasphaera	Megasphaera_micronuciformis	0.3644	0.6185	0.2797	0.3945	0.5297	1
TAU	t0 vs. t6	t0	t6	14	Veillonella	Veillonella_atypica	1.1166	1.4375	1.0798	2.2323	0.9353	1
TAU	t0 vs. t6	t0	t6	14	Veillonella	Veillonella_parvula	3.1847	2.6967	1.9448	1.3875	0.1284	1
TAU	t0 vs. t6	t0	t6	14	Veillonella	Veillonella_rogosae	0.0667	0.1721	0.0024	0.0050	0.1811	1
TAU	t0 vs. t6	t0	t6	14	Fusobacterium	Fusobacterium_nucleatum	0.6820	0.5541	0.7732	0.4778	0.5488	1
TAU	t0 vs. t6	t0	t6	14	Fusobacterium	Fusobacterium_periodonticum	1.2853	0.8996	1.7886	1.3947	0.1837	1
TAU	t0 vs. t6	t0	t6	14	Leptotrichia	Leptotrichia_buccalis	0.2261	0.3980	0.3284	0.5854	0.5053	1
TAU	t0 vs. t6	t0	t6	14	Leptotrichia	Leptotrichia_goodfellowii	0.0021	0.0070	0.0030	0.0081	0.6916	1
TAU	t0 vs. t6	t0	t6	14	Leptotrichia	Leptotrichia_hofstadii	0.0057	0.0094	0.0144	0.0489	0.5369	1
TAU	t0 vs. t6	t0	t6	14	Leptotrichia	Leptotrichia_hongkongensis	0.0007	0.0019	0.0007	0.0013	0.8353	1
TAU	t0 vs. t6	t0	t6	14	Leptotrichia	Leptotrichia_shahii	0.0302	0.0740	0.0063	0.0161	0.2436	1
TAU	t0 vs. t6	t0	t6	14	Leptotrichia	Leptotrichia_trevisanii	0.0002	0.0006	0.0015	0.0038	0.1779	1
TAU	t0 vs. t6	t0	t6	14	Leptotrichia	Leptotrichia_wadei	0.2824	0.5199	0.3245	0.6620	0.8284	1
TAU	t0 vs. t6	t0	t6	14	Absconditabacteriales_(SR1)	SR1_bacterium	0.0087	0.0185	0.0093	0.0150	0.6605	1
TAU	t0 vs. t6	t0	t6	14	Gracilibacteria	Gracilibacteria_bacterium	0.0001	0.0004	0.0005	0.0010	0.0997	1
TAU	t0 vs. t6	t0	t6	14	JGI_0000069-P22	Gracilibacteria_bacterium	0.0001	0.0004	0.0006	0.0018	0.2178	1
TAU	t0 vs. t6	t0	t6	14	Candidatus_Saccharimonas	Candidatus_Saccharimonas	0.0002	0.0008	0.0003	0.0010	0.3356	1
TAU	t0 vs. t6	t0	t6	14	Candidatus_Saccharimonas	TM7_phylum	0.1099	0.1437	0.1411	0.3171	0.5974	1
TAU	t0 vs. t6	t0	t6	14	Saccharimonadaceae	TM7_bacterium	0.0047	0.0065	0.0023	0.0041	0.1999	1
TAU	t0 vs. t6	t0	t6	14	Saccharimonadaceae	TM7_phylum	0.0007	0.0018	0.0009	0.0026	0.5310	1
TAU	t0 vs. t6	t0	t6	14	TM7a	candidate_division	0.0001	0.0005	0.0001	0.0002	0.5593	1
TAU	t0 vs. t6	t0	t6	14	TM7x	Candidatus_Saccharibacteria	0.1080	0.2096	0.2052	0.3047	0.3480	1
TAU	t0 vs. t6	t0	t6	14	TM7x	TM7_phylum	0.2541	0.3426	0.2949	0.4808	0.7808	1
TAU	t0 vs. t6	t0	t6	14	Saccharimonadales	Candidatus_Saccharibacteria	0.0009	0.0028	0.0001	0.0005	0.3291	1
TAU	t0 vs. t6	t0	t6	14	Saccharimonadales	TM7_phylum	0.2625	0.3391	0.2275	0.4482	0.6757	1
TAU	t0 vs. t6	t0	t6	14	Shewanella	Shewanella_putrefaciens	0.0037	0.0065	0.0008	0.0016	0.1467	1
TAU	t0 vs. t6	t0	t6	14	Lautropia	Lautropia_mirabilis	0.0919	0.2130	0.1714	0.3795	0.1305	1
TAU	t0 vs. t6	t0	t6	14	Brachymonas	Brachymonas_denitrificans	0.0030	0.0076	0.0043	0.0147	0.7292	1
TAU	t0 vs. t6	t0	t6	14	Eikenella	Eikenella_corrodens	0.0000	0.0001	0.0003	0.0010	0.3969	1
TAU	t0 vs. t6	t0	t6	14	Kingella	Kingella_denitrificans	0.0003	0.0011	0.0004	0.0013	0.8653	1
TAU	t0 vs. t6	t0	t6	14	Kingella	Kingella_oralis	0.0015	0.0048	0.0024	0.0090	0.7396	1
TAU	t0 vs. t6	t0	t6	14	Neisseria	Neisseria_bacilliformis	0.0017	0.0048	0.0263	0.0984	0.3686	1
TAU	t0 vs. t6	t0	t6	14	Neisseria	Neisseria_elongata	0.0156	0.0245	0.0233	0.0452	0.5554	1
TAU	t0 vs. t6	t0	t6	14	Neisseria	Neisseria_meningitidis	7.9093	4.4844	9.9745	6.7115	0.1860	1

TAU	t0 vs. t6	t0	t6	14	Neisseria	Neisseria_oralis	0.0097	0.0192	0.0251	0.0416	0.0596	1
TAU	t0 vs. t6	t0	t6	14	Neisseria	Neisseria_perflava	0.0025	0.0039	0.0085	0.0177	0.2480	1
TAU	t0 vs. t6	t0	t6	14	Simonsiella	Simonsiella_muelleri	0.0056	0.0180	0.0056	0.0167	0.8802	1
TAU	t0 vs. t6	t0	t6	14	Cardiobacterium	Cardiobacterium_hominis	0.0045	0.0060	0.0085	0.0146	0.3140	1
TAU	t0 vs. t6	t0	t6	14	Cardiobacterium	Cardiobacterium_valvarum	0.0077	0.0090	0.0061	0.0077	0.6198	1
TAU	t0 vs. t6	t0	t6	14	Actinobacillus	Actinobacillus_pleuropneumoniae	0.0213	0.0401	0.0383	0.0545	0.1644	1
TAU	t0 vs. t6	t0	t6	14	Actinobacillus	Haemophilus_parahaemolyticus	0.0148	0.0334	0.0077	0.0181	0.4097	1
TAU	t0 vs. t6	t0	t6	14	Aggregatibacter	Aggregatibacter_aphrophilus	0.0763	0.0955	0.1219	0.2855	0.4937	1
TAU	t0 vs. t6	t0	t6	14	Aggregatibacter	Aggregatibacter_segnis	0.0298	0.0368	0.0230	0.0409	0.5122	1
TAU	t0 vs. t6	t0	t6	14	Haemophilus	[Haemophilus]_ducreyi	0.0021	0.0057	0.0037	0.0104	0.6394	1
TAU	t0 vs. t6	t0	t6	14	Haemophilus	Haemophilus_haemolyticus	0.0561	0.0823	0.0749	0.0798	0.3919	1
TAU	t0 vs. t6	t0	t6	14	Haemophilus	Haemophilus_influenzae	0.0390	0.0514	0.0673	0.0873	0.2220	1
TAU	t0 vs. t6	t0	t6	14	Haemophilus	Haemophilus_parainfluenzae	0.9739	0.7214	1.6545	0.8205	0.0393	1
TAU	t0 vs. t6	t0	t6	14	Haemophilus	Haemophilus_pittmaniae	0.5408	0.5744	0.8513	0.6608	0.1573	1
TAU	t0 vs. t6	t0	t6	14	Haemophilus	Haemophilus_sputorum	0.0248	0.0410	0.0826	0.1532	0.1397	1
TAU	t0 vs. t6	t0	t6	14	Mannheimia	Mannheimia_haemolytica	0.0213	0.0310	0.0252	0.0428	0.7333	1
TAU	t0 vs. t6	t0	t6	14	Pasteurella	Pasteurella_multocida	0.4734	0.5217	0.7419	0.5436	0.1458	1
TAU	t0 vs. t6	t0	t6	14	Rodentibacter	Pasteurellaceae_bacterium	0.0048	0.0123	0.0373	0.0607	0.0721	1
TAU	t0 vs. t6	t0	t6	14	Acinetobacter	Acinetobacter_johnsonii	0.0006	0.0018	0.0016	0.0027	0.2854	1
TAU	t0 vs. t6	t0	t6	14	Treponema	Treponema_medium	0.0030	0.0071	0.0089	0.0202	0.1360	1
TAU	t0 vs. t6	t0	t6	14	Treponema	Treponema_refringens	0.0000	0.0001	0.0002	0.0005	0.1673	1
TAU	t3 vs. t6	t3	t6	14	Actinomyces	Actinomyces_dentalis	0.0006	0.0016	0.0009	0.0025	0.8090	1
TAU	t3 vs. t6	t3	t6	14	Actinomyces	Actinomyces_graevenitzii	0.8060	1.2588	1.0914	1.3939	0.5615	1
TAU	t3 vs. t6	t3	t6	14	Actinomyces	Actinomyces_lingnae	0.0003	0.0011	0.0003	0.0012	0.9641	1
TAU	t3 vs. t6	t3	t6	14	Actinomyces	Actinomyces_naeslundii	0.0323	0.0556	0.0355	0.0855	0.8224	1
TAU	t3 vs. t6	t3	t6	14	Actinomyces	Actinomyces_oris	0.0039	0.0086	0.0016	0.0055	0.0648	1
TAU	t3 vs. t6	t3	t6	14	Actinomyces	Actinomyces_viscosus	0.0006	0.0022	0.0004	0.0011	0.7477	1
TAU	t3 vs. t6	t3	t6	14	Actinomyces	Schaalia_odontolytica	0.3476	0.4341	0.3805	0.4124	0.8325	1
TAU	t3 vs. t6	t3	t6	14	Alloscardovia	Alloscardovia_omnicolens	0.0016	0.0061	0.0128	0.0272	0.1404	1
TAU	t3 vs. t6	t3	t6	14	Bifidobacterium	Bifidobacterium_longum	0.0010	0.0039	0.0043	0.0162	0.3356	1
TAU	t3 vs. t6	t3	t6	14	Scardovia	Scardovia_inopinata	0.0002	0.0006	0.0001	0.0005	0.8427	1
TAU	t3 vs. t6	t3	t6	14	Scardovia	Scardovia_wiggsiae	0.0002	0.0005	0.0000	0.0001	0.2865	1
TAU	t3 vs. t6	t3	t6	14	Corynebacterium	Corynebacterium_diphtheriae	0.0013	0.0042	0.0006	0.0019	0.5685	1
TAU	t3 vs. t6	t3	t6	14	Corynebacterium	Corynebacterium_durum	0.0098	0.0218	0.0169	0.0485	0.4066	1
TAU	t3 vs. t6	t3	t6	14	Corynebacterium	Corynebacterium_matruchotii	0.0047	0.0084	0.0025	0.0053	0.2895	1
TAU	t3 vs. t6	t3	t6	14	Rothia	Rothia_aeria	0.0697	0.0667	0.1222	0.1884	0.3472	1
TAU	t3 vs. t6	t3	t6	14	Rothia	Rothia_mucilaginosa	1.7984	1.4481	3.8246	3.4970	0.0394	1
TAU	t3 vs. t6	t3	t6	14	Pseudopropionibacterium	Pseudopropionibacterium_propionicum	0.0001	0.0002	0.0002	0.0006	0.5822	1
TAU	t3 vs. t6	t3	t6	14	Atopobium	Lancefieldella_parvula	0.0226	0.0410	0.0210	0.0276	0.9101	1
TAU	t3 vs. t6	t3	t6	14	Cryptobacterium	Cryptobacterium_curtum	0.0001	0.0003	0.0008	0.0031	0.3356	1
TAU	t3 vs. t6	t3	t6	14	Bacteroides	Bacteroidaceae_bacterium	0.0058	0.0160	0.0004	0.0011	0.2243	1
TAU	t3 vs. t6	t3	t6	14	F0058	Bacteroidetes_oral	0.0011	0.0039	0.0045	0.0091	0.2172	1
TAU	t3 vs. t6	t3	t6	14	Porphyromonas	Porphyromonas_catoniae	0.0255	0.0347	0.0174	0.0295	0.4517	1
TAU	t3 vs. t6	t3	t6	14	Porphyromonas	Porphyromonas_endodontalis	0.0071	0.0132	0.0076	0.0198	0.9366	1
TAU	t3 vs. t6	t3	t6	14	Porphyromonas	Porphyromonas_gingivalis	0.0003	0.0013	0.0002	0.0006	0.6474	1
TAU	t3 vs. t6	t3	t6	14	Porphyromonas	Porphyromonas_pasteri	1.4984	1.4107	1.2826	0.9950	0.5288	1
TAU	t3 vs. t6	t3	t6	14	Alloprevotella	Alloprevotella_rava	0.2204	0.6478	0.1972	0.5037	0.9135	1
TAU	t3 vs. t6	t3	t6	14	Alloprevotella	Alloprevotella_tannerae	0.1573	0.4228	0.0871	0.2653	0.2382	1
TAU	t3 vs. t6	t3	t6	14	Alloprevotella	Prevotellaceae_bacterium	0.5907	0.5444	0.8402	0.9999	0.4311	1
TAU	t3 vs. t6	t3	t6	14	Prevotella	Prevotella_aurantiaca	0.1213	0.2637	0.1461	0.4323	0.8308	1

TAU	t3 vs. t6	t3	t6	14	Prevotella	Prevotella_baroniae	0.0010	0.0036	0.0000	0.0001	0.3546	1
TAU	t3 vs. t6	t3	t6	14	Prevotella	Prevotella_denticola	0.0084	0.0210	0.0045	0.0094	0.3137	1
TAU	t3 vs. t6	t3	t6	14	Prevotella	Prevotella_enoeca	0.0003	0.0012	0.0004	0.0014	0.9036	1
TAU	t3 vs. t6	t3	t6	14	Prevotella	Prevotella_fusca	0.0059	0.0153	0.0010	0.0030	0.2233	1
TAU	t3 vs. t6	t3	t6	14	Prevotella	Prevotella_histicola	0.2243	0.4722	0.5654	1.2562	0.1900	1
TAU	t3 vs. t6	t3	t6	14	Prevotella	Prevotella_intermedia	0.3327	0.5727	0.1814	0.2459	0.3862	1
TAU	t3 vs. t6	t3	t6	14	Prevotella	Prevotella_jejuni	0.0483	0.1707	0.6865	1.9642	0.2075	1
TAU	t3 vs. t6	t3	t6	14	Prevotella	Prevotella_loescheii	0.1239	0.2839	0.4095	0.6987	0.0918	1
TAU	t3 vs. t6	t3	t6	14	Prevotella	Prevotella_maculosa	0.0005	0.0017	0.0003	0.0010	0.7520	1
TAU	t3 vs. t6	t3	t6	14	Prevotella	Prevotella_marshii	0.0004	0.0010	0.0001	0.0003	0.3911	1
TAU	t3 vs. t6	t3	t6	14	Prevotella	Prevotella_melaninogenica	9.0394	6.8063	7.7778	5.8302	0.5650	1
TAU	t3 vs. t6	t3	t6	14	Prevotella	Prevotella_nanceiensis	0.8250	1.1630	0.7252	1.1135	0.5669	1
TAU	t3 vs. t6	t3	t6	14	Prevotella	Prevotella_nigrescens	0.0475	0.1114	0.0217	0.0317	0.3538	1
TAU	t3 vs. t6	t3	t6	14	Prevotella	Prevotella_oralis	0.0004	0.0011	0.0008	0.0020	0.1753	1
TAU	t3 vs. t6	t3	t6	14	Prevotella	Prevotella_oulorum	0.0021	0.0037	0.0788	0.1526	0.0814	1
TAU	t3 vs. t6	t3	t6	14	Prevotella	Prevotella_pallens	0.7994	1.8524	0.5643	0.7183	0.6621	1
TAU	t3 vs. t6	t3	t6	14	Prevotella	Prevotella_pleuritidis	0.0034	0.0093	0.0012	0.0043	0.1526	1
TAU	t3 vs. t6	t3	t6	14	Prevotella	Prevotella_saccharolytica	0.0007	0.0017	0.0011	0.0029	0.6612	1
TAU	t3 vs. t6	t3	t6	14	Prevotella	Prevotella_salivae	0.8038	1.4456	0.8292	1.0827	0.9392	1
TAU	t3 vs. t6	t3	t6	14	Prevotella	Prevotella_shahii	0.0436	0.1006	0.1639	0.2642	0.0842	1
TAU	t3 vs. t6	t3	t6	14	Prevotella	Prevotella_veroralis	0.0017	0.0058	0.0005	0.0014	0.4971	1
TAU	t3 vs. t6	t3	t6	14	Rikenellaceae_RC9_gut_group	Bacteroidales_oral	0.0019	0.0061	0.0001	0.0002	0.2842	1
TAU	t3 vs. t6	t3	t6	14	Rikenellaceae_RC9_gut_group	Bacteroidia_bacterium	0.0001	0.0005	0.0000	0.0001	0.4772	1
TAU	t3 vs. t6	t3	t6	14	Tannerella	Tannerella_forsythia	0.0035	0.0053	0.0302	0.0532	0.0918	1
TAU	t3 vs. t6	t3	t6	14	Capnocytophaga	Capnocytophaga_gingivalis	0.0524	0.0626	0.0622	0.0535	0.6326	1
TAU	t3 vs. t6	t3	t6	14	Capnocytophaga	Capnocytophaga_granulosa	0.0023	0.0056	0.0032	0.0071	0.6627	1
TAU	t3 vs. t6	t3	t6	14	Capnocytophaga	Capnocytophaga_leadbetteri	0.0627	0.0987	0.1427	0.2444	0.1316	1
TAU	t3 vs. t6	t3	t6	14	Capnocytophaga	Capnocytophaga_ochracea	0.0403	0.0941	0.0697	0.0924	0.3755	1
TAU	t3 vs. t6	t3	t6	14	Capnocytophaga	Capnocytophaga_sputigena	0.0857	0.1200	0.0902	0.1654	0.8360	1
TAU	t3 vs. t6	t3	t6	14	Flavobacterium	Flavobacterium_branchiophilum	0.0001	0.0005	0.0002	0.0005	0.8632	1
TAU	t3 vs. t6	t3	t6	14	Bergeyella	Flavobacteriaceae_bacterium	0.0004	0.0011	0.0015	0.0033	0.3120	1
TAU	t3 vs. t6	t3	t6	14	Campylobacter	Campylobacter_concisus	0.0456	0.0657	0.0824	0.1940	0.5183	1
TAU	t3 vs. t6	t3	t6	14	Campylobacter	Campylobacter_gracilis	0.0002	0.0006	0.0001	0.0004	0.8905	1
TAU	t3 vs. t6	t3	t6	14	Campylobacter	Campylobacter_showae	0.0021	0.0040	0.0046	0.0063	0.2442	1
TAU	t3 vs. t6	t3	t6	14	Solobacterium	Solobacterium_moorei	0.2233	0.2117	0.2692	0.4013	0.6725	1
TAU	t3 vs. t6	t3	t6	14	Globicatella	Aerococcaceae_bacterium	0.0117	0.0371	0.0035	0.0101	0.4519	1
TAU	t3 vs. t6	t3	t6	14	Granulicatella	Granulicatella_adiacens	0.0091	0.0266	0.0011	0.0027	0.2794	1
TAU	t3 vs. t6	t3	t6	14	Granulicatella	Granulicatella_elegans	0.0407	0.1143	0.0403	0.1063	0.9934	1
TAU	t3 vs. t6	t3	t6	14	Enterococcus	Enterococcus_columbae	0.0005	0.0020	0.0005	0.0017	0.3356	1
TAU	t3 vs. t6	t3	t6	14	Lactobacillus	Lactobacillus_acidophilus	0.0001	0.0004	0.0021	0.0079	0.3623	1
TAU	t3 vs. t6	t3	t6	14	Lactobacillus	Lactobacillus_amylovorus	0.0006	0.0023	0.0262	0.0978	0.3356	1
TAU	t3 vs. t6	t3	t6	14	Lactobacillus	Lactobacillus_helveticus	0.0022	0.0081	0.0767	0.2851	0.3321	1
TAU	t3 vs. t6	t3	t6	14	Lactobacillus	Lactobacillus_reuteri	0.0012	0.0044	0.0226	0.0844	0.3356	1
TAU	t3 vs. t6	t3	t6	14	Lactobacillus	Lactobacillus_sakei	0.0067	0.0166	0.0003	0.0013	0.1806	1
TAU	t3 vs. t6	t3	t6	14	Lactobacillus	Lactobacillus_vaginalis	0.0009	0.0033	0.0152	0.0568	0.3356	1
TAU	t3 vs. t6	t3	t6	14	Streptococcus	Streptococcus_anginosus	0.0184	0.0472	0.0057	0.0128	0.2065	1
TAU	t3 vs. t6	t3	t6	14	Streptococcus	Streptococcus_australis	0.0161	0.0228	0.0086	0.0099	0.1439	1
TAU	t3 vs. t6	t3	t6	14	Streptococcus	Streptococcus_cristatus	0.0180	0.0269	0.0084	0.0135	0.0808	1
TAU	t3 vs. t6	t3	t6	14	Streptococcus	Streptococcus_gordonii	0.0241	0.0249	0.0229	0.0308	0.9069	1
TAU	t3 vs. t6	t3	t6	14	Streptococcus	Streptococcus_infantis	0.0336	0.0330	0.0325	0.0291	0.9188	1

TAU	t3 vs. t6	t3	t6	14	Streptococcus	Streptococcus_mitis	0.0101	0.0143	0.0061	0.0107	0.3589	1
TAU	t3 vs. t6	t3	t6	14	Streptococcus	Streptococcus_oralis	0.0090	0.0193	0.0092	0.0148	0.9511	1
TAU	t3 vs. t6	t3	t6	14	Streptococcus	Streptococcus_parasanguinis	2.3392	4.7323	0.8225	0.7793	0.2038	1
TAU	t3 vs. t6	t3	t6	14	Streptococcus	Streptococcus_pneumoniae	0.7804	1.2352	0.3300	0.2997	0.1800	1
TAU	t3 vs. t6	t3	t6	14	Streptococcus	Streptococcus_salivarius	7.2355	8.4936	3.6240	3.5179	0.1169	1
TAU	t3 vs. t6	t3	t6	14	Streptococcus	Streptococcus_sanguinis	0.1321	0.1824	0.1676	0.4113	0.7776	1
TAU	t3 vs. t6	t3	t6	14	Streptococcus	Streptococcus_suis	0.4049	0.4859	0.3750	0.3406	0.8421	1
TAU	t3 vs. t6	t3	t6	14	RF39	Firmicutes_oral	0.0023	0.0059	0.0037	0.0120	0.4564	1
TAU	t3 vs. t6	t3	t6	14	Gemella	Gemella_haemolysans	1.6567	1.2316	1.6337	0.8754	0.9432	1
TAU	t3 vs. t6	t3	t6	14	Gemella	Gemella_morbillorum	0.0154	0.0205	0.0125	0.0179	0.6575	1
TAU	t3 vs. t6	t3	t6	14	Gemella	Gemella_sanguinis	0.1308	0.1304	0.1122	0.0867	0.6245	1
TAU	t3 vs. t6	t3	t6	14	Staphylococcus	Staphylococcus_aureus	0.0010	0.0037	0.0001	0.0003	0.3356	1
TAU	t3 vs. t6	t3	t6	14	Clostridia_UCG-014	Clostridiales_bacterium	0.1164	0.0950	0.3902	0.5506	0.0853	1
TAU	t3 vs. t6	t3	t6	14	Defluviitaleaceae_UCG-011	Lachnospiraceae_bacterium	0.0046	0.0118	0.0066	0.0243	0.7572	1
TAU	t3 vs. t6	t3	t6	14	Catonella	Catonella_morbi	0.0515	0.0636	0.0480	0.0869	0.8476	1
TAU	t3 vs. t6	t3	t6	14	Howardella	Howardella_ureilytica	0.0032	0.0118	0.0028	0.0095	0.5558	1
TAU	t3 vs. t6	t3	t6	14	Johnsonella	Firmicutes_oral	0.0003	0.0009	0.0002	0.0007	0.7383	1
TAU	t3 vs. t6	t3	t6	14	Johnsonella	Johnsonella_ignava	0.0001	0.0002	0.0001	0.0003	0.9455	1
TAU	t3 vs. t6	t3	t6	14	Johnsonella	Lachnospiraceae_bacterium	0.0021	0.0055	0.0106	0.0387	0.3932	1
TAU	t3 vs. t6	t3	t6	14	Lachnoanaerobaculum	Lachnoanaerobaculum_umeaense	0.2012	0.2587	0.1319	0.1106	0.1472	1
TAU	t3 vs. t6	t3	t6	14	Shuttleworthia	Shuttleworthia_satelles	0.0083	0.0282	0.0013	0.0041	0.2986	1
TAU	t3 vs. t6	t3	t6	14	Stomatobaculum	Stomatobaculum_longum	0.1118	0.2165	0.0529	0.0545	0.3370	1
TAU	t3 vs. t6	t3	t6	14	[Eubacterium]_brachy_group	[Eubacterium]_brachy	0.0013	0.0041	0.0003	0.0007	0.3407	1
TAU	t3 vs. t6	t3	t6	14	Amnipila	Peptostreptococcaceae_bacterium	0.0053	0.0196	0.0035	0.0125	0.3800	1
TAU	t3 vs. t6	t3	t6	14	Family_XIII_UCG-001	Peptostreptococcaceae_bacterium	0.0146	0.0414	0.0015	0.0055	0.2436	1
TAU	t3 vs. t6	t3	t6	14	[Eubacterium]_yurii_group	[Eubacterium]_yurii	0.0227	0.0437	0.0143	0.0255	0.5246	1
TAU	t3 vs. t6	t3	t6	14	Filifactor	Filifactor_alocis	0.0062	0.0168	0.0036	0.0128	0.6573	1
TAU	t3 vs. t6	t3	t6	14	Parvimonas	Parvimonas_micra	0.0060	0.0113	0.0033	0.0068	0.1961	1
TAU	t3 vs. t6	t3	t6	14	Selenomonas	Selenomonas_flueggei	0.0019	0.0069	0.0886	0.3317	0.3462	1
TAU	t3 vs. t6	t3	t6	14	Selenomonas	Selenomonas_infelix	0.0014	0.0053	0.0007	0.0027	0.6846	1
TAU	t3 vs. t6	t3	t6	14	Selenomonas	Selenomonas_massiliensis	0.0048	0.0123	0.0099	0.0223	0.3721	1
TAU	t3 vs. t6	t3	t6	14	Selenomonas	Selenomonas_noxia	0.0288	0.0719	0.0103	0.0229	0.2294	1
TAU	t3 vs. t6	t3	t6	14	Selenomonas	Selenomonas_sputigena	0.0231	0.0688	0.0378	0.0875	0.2495	1
TAU	t3 vs. t6	t3	t6	14	Dialister	Dialister_invisus	0.2028	0.6939	0.0460	0.1042	0.4226	1
TAU	t3 vs. t6	t3	t6	14	Dialister	Dialister_micraerophilus	0.0064	0.0240	0.0010	0.0025	0.3842	1
TAU	t3 vs. t6	t3	t6	14	Dialister	Dialister_pneumosintes	0.0350	0.1309	0.0026	0.0078	0.3716	1
TAU	t3 vs. t6	t3	t6	14	Megasphaera	Megasphaera_micronuciformis	0.3647	1.0791	0.2797	0.3945	0.7099	1
TAU	t3 vs. t6	t3	t6	14	Veillonella	Veillonella_atypica	0.8052	1.4862	1.0798	2.2323	0.3101	1
TAU	t3 vs. t6	t3	t6	14	Veillonella	Veillonella_parvula	2.7131	2.3542	1.9448	1.3875	0.2135	1
TAU	t3 vs. t6	t3	t6	14	Veillonella	Veillonella_rogosae	0.0717	0.2644	0.0024	0.0050	0.3373	1
TAU	t3 vs. t6	t3	t6	14	Fusobacterium	Fusobacterium_nucleatum	0.9028	0.8307	0.7732	0.4778	0.6508	1
TAU	t3 vs. t6	t3	t6	14	Fusobacterium	Fusobacterium_periodonticum	1.7330	1.3193	1.7886	1.3947	0.9192	1
TAU	t3 vs. t6	t3	t6	14	Leptotrichia	Leptotrichia_buccalis	0.1623	0.2872	0.3284	0.5854	0.3791	1
TAU	t3 vs. t6	t3	t6	14	Leptotrichia	Leptotrichia_goodfellowii	0.0014	0.0035	0.0030	0.0081	0.5207	1
TAU	t3 vs. t6	t3	t6	14	Leptotrichia	Leptotrichia_hofstadii	0.0062	0.0113	0.0144	0.0489	0.5549	1
TAU	t3 vs. t6	t3	t6	14	Leptotrichia	Leptotrichia_hongkongensis	0.0017	0.0036	0.0007	0.0013	0.2432	1
TAU	t3 vs. t6	t3	t6	14	Leptotrichia	Leptotrichia_shahii	0.0194	0.0704	0.0063	0.0161	0.4129	1
TAU	t3 vs. t6	t3	t6	14	Leptotrichia	Leptotrichia_trevisanii	0.0002	0.0006	0.0015	0.0038	0.1666	1
TAU	t3 vs. t6	t3	t6	14	Leptotrichia	Leptotrichia_wadei	0.0592	0.0650	0.3245	0.6620	0.1701	1
TAU	t3 vs. t6	t3	t6	14	Absconditabacteriales_(SR1)	SR1_bacterium	0.0080	0.0136	0.0093	0.0150	0.6595	1

TAU	t3 vs. t6	t3	t6	14	Gracilibacteria	Gracilibacteria_bacterium	0.0002	0.0009	0.0005	0.0010	0.3762	1
TAU	t3 vs. t6	t3	t6	14	JGI_0000069-P22	Gracilibacteria_bacterium	0.0006	0.0014	0.0006	0.0018	0.9301	1
TAU	t3 vs. t6	t3	t6	14	Candidatus_Saccharimonas	TM7_phylum	0.0741	0.0771	0.1411	0.3171	0.3786	1
TAU	t3 vs. t6	t3	t6	14	Saccharimonadaceae	TM7_bacterium	0.0050	0.0106	0.0023	0.0041	0.3171	1
TAU	t3 vs. t6	t3	t6	14	Saccharimonadaceae	TM7_phylum	0.0004	0.0010	0.0009	0.0026	0.3766	1
TAU	t3 vs. t6	t3	t6	14	TM7a	candidate_division	0.0003	0.0004	0.0001	0.0002	0.0772	1
TAU	t3 vs. t6	t3	t6	14	TM7a	TM7_phylum	0.0005	0.0018	0.0003	0.0007	0.6822	1
TAU	t3 vs. t6	t3	t6	14	TM7x	Candidatus_Saccharibacteria	0.0630	0.0796	0.2052	0.3047	0.1268	1
TAU	t3 vs. t6	t3	t6	14	TM7x	TM7_phylum	0.1468	0.2644	0.2949	0.4808	0.3075	1
TAU	t3 vs. t6	t3	t6	14	Saccharimonadales	Candidatus_Saccharibacteria	0.0004	0.0014	0.0001	0.0005	0.5378	1
TAU	t3 vs. t6	t3	t6	14	Saccharimonadales	TM7_phylum	0.0723	0.1054	0.2275	0.4482	0.1352	1
TAU	t3 vs. t6	t3	t6	14	Shewanella	Shewanella_putrefaciens	0.0030	0.0056	0.0008	0.0016	0.1818	1
TAU	t3 vs. t6	t3	t6	14	Lautropia	Lautropia_mirabilis	0.0756	0.1104	0.1714	0.3795	0.3483	1
TAU	t3 vs. t6	t3	t6	14	Brachymonas	Brachymonas_denitrificans	0.0011	0.0018	0.0043	0.0147	0.4297	1
TAU	t3 vs. t6	t3	t6	14	Eikenella	Eikenella_corrodens	0.0007	0.0013	0.0003	0.0010	0.3940	1
TAU	t3 vs. t6	t3	t6	14	Kingella	Kingella_oralis	0.0097	0.0335	0.0024	0.0090	0.4540	1
TAU	t3 vs. t6	t3	t6	14	Neisseria	Neisseria_bacilliformis	0.0040	0.0126	0.0263	0.0984	0.3485	1
TAU	t3 vs. t6	t3	t6	14	Neisseria	Neisseria_elongata	0.0186	0.0267	0.0233	0.0452	0.5685	1
TAU	t3 vs. t6	t3	t6	14	Neisseria	Neisseria_meningitidis	7.4738	4.6829	9.9745	6.7115	0.1488	1
TAU	t3 vs. t6	t3	t6	14	Neisseria	Neisseria_oralis	0.0475	0.1089	0.0251	0.0416	0.4102	1
TAU	t3 vs. t6	t3	t6	14	Neisseria	Neisseria_perflava	0.0036	0.0065	0.0085	0.0177	0.3529	1
TAU	t3 vs. t6	t3	t6	14	Simonsiella	Simonsiella_muelleri	0.0006	0.0015	0.0056	0.0167	0.2528	1
TAU	t3 vs. t6	t3	t6	14	Cardiobacterium	Cardiobacterium_hominis	0.0057	0.0091	0.0085	0.0146	0.5537	1
TAU	t3 vs. t6	t3	t6	14	Cardiobacterium	Cardiobacterium_valvarum	0.0048	0.0063	0.0061	0.0077	0.7031	1
TAU	t3 vs. t6	t3	t6	14	Escherichia-Shigella	Escherichia_coli	0.0010	0.0035	0.0001	0.0003	0.3637	1
TAU	t3 vs. t6	t3	t6	14	Actinobacillus	Actinobacillus_pleuropneumoniae	0.0279	0.0549	0.0383	0.0545	0.6401	1
TAU	t3 vs. t6	t3	t6	14	Actinobacillus	Haemophilus_parahaemolyticus	0.0036	0.0085	0.0077	0.0181	0.4284	1
TAU	t3 vs. t6	t3	t6	14	Aggregatibacter	Aggregatibacter_aphrophilus	0.1166	0.2007	0.1219	0.2855	0.9523	1
TAU	t3 vs. t6	t3	t6	14	Aggregatibacter	Aggregatibacter_segnis	0.0146	0.0256	0.0230	0.0409	0.5512	1
TAU	t3 vs. t6	t3	t6	14	Haemophilus	[Haemophilus]_ducreyi	0.0032	0.0096	0.0037	0.0104	0.9068	1
TAU	t3 vs. t6	t3	t6	14	Haemophilus	Haemophilus_haemolyticus	0.0935	0.1163	0.0749	0.0798	0.4024	1
TAU	t3 vs. t6	t3	t6	14	Haemophilus	Haemophilus_influenzae	0.0908	0.1046	0.0673	0.0873	0.3425	1
TAU	t3 vs. t6	t3	t6	14	Haemophilus	Haemophilus_parainfluenzae	1.2198	0.6891	1.6545	0.8205	0.0906	1
TAU	t3 vs. t6	t3	t6	14	Haemophilus	Haemophilus_pittmaniae	0.5650	0.4262	0.8513	0.6608	0.0783	1
TAU	t3 vs. t6	t3	t6	14	Haemophilus	Haemophilus_sputorum	0.0446	0.0627	0.0826	0.1532	0.4144	1
TAU	t3 vs. t6	t3	t6	14	Mannheimia	Mannheimia_haemolytica	0.0424	0.0881	0.0252	0.0428	0.3863	1
TAU	t3 vs. t6	t3	t6	14	Pasteurella	Pasteurella_multocida	0.8594	0.7523	0.7419	0.5436	0.6548	1
TAU	t3 vs. t6	t3	t6	14	Rodentibacter	Pasteurellaceae_bacterium	0.0310	0.1001	0.0373	0.0607	0.8209	1
TAU	t3 vs. t6	t3	t6	14	Acinetobacter	Acinetobacter_johnsonii	0.0007	0.0011	0.0016	0.0027	0.2758	1
TAU	t3 vs. t6	t3	t6	14	Treponema	Treponema_medium	0.0036	0.0092	0.0089	0.0202	0.2356	1
TAU	t3 vs. t6	t3	t6	14	Treponema	Treponema_refringens	0.0003	0.0011	0.0002	0.0005	0.7929	1

Abbreviations: OPP, Intensive oral prophylaxis program; TAU, Treatmen-As-Usual

Supplementary table S6: Cluster of gingivitis- and health-associated bacteria

A) Cluster of gingivitis-associated bacteria	
**Meta-Analysis by Abusleme et al.**	**Species in our dataset**
Alloprevotella_tannerae	**Alloprevotella_tannerae**
Tanerella_sp_HMT_286	**Tannerella_forsythia**
Leptotrichia_sp_HMT_498	not found
Leptotrichia_shahii	**Leptotrichia_shahii***
Leptotrichia_buccalis	**Leptotrichia_buccalis**
Leptotrichia_sp._HMT_212	not found
Leptotrichia_sp._HMT_215	not found
Abiotrophia_defectiva	not found
Streptococcus_oralis_subsp._dentisani_clade_398	**Streptococcus_oralis**
Streptococcus_oralis_subsp._dentisani_clade_071	**Streptococcus_oralis**
Streptococcus_oralis_subsp._dentisani_clade_058	**Streptococcus_oralis**
Neisseria_flavescens_subflava	not found
Leptotrichia_sp._HMT_225	not found
Porphyromonas_pasteri	**Porphyromonas_pasteri**
Leptotrichia_hongkongensis	**Leptotrichia_hongkongensis**
Actinomyces_sp._HMT_169	**Actinomyces_naeslundi**
Leptotrichia_wadeii	**Leptotrichia_wadeii**
Leptotrichia_hofstadii	**Leptotrichia_hofstadii**
Streptococcus_cristatus_clade_578	**Streptococcus cristatus**
Neisseria_sicca	not found
Prevotella_melaninogenica	**Prevotella_melaninogenica**
Neisseria_sicca	not found
Neisseria_macacae	not found
Fusobacterium_nucleatum_subsp_polymorphum	not found
Fusobacterium_periodonticum	**Fusobacterium_periodonticum**
Leptotrichia_sp._HMT_392	not found
Leptotrichia_sp._HMT_417	not found

B) Cluster of kealth-associated bacteria	
**Meta-Analysis by Abusleme et al.**	**Species in our dataset**
Streptococcus_HMT_423_mitis_pneumoniae	**Streptococcus_mitis**
Rothia_aeria	**Rothia_aeria**
Corynebacterium_matruchotii	**Corynebacterium_matruchotii**
Lautropia_mirabilis	**Lautropia_mirabilis**
Actinomyces_sp._HMT_175	not found
Neisseria_flava	not found
Fusobacterium_nucleatum_subsp._vincentii	not found
Actinomyces_oris	**Actinomyces_oris**
Capnocytophaga_granulosa	**Capnocytophaga_granulosa**
Rothia_dentocariosa	not found
Corynebacterium_durum	**Corynebacterium_durum**
Actinomyces_sp._HMT_171	not found
Bergeyella_sp_HTML_322	not found
Gemella haemolysans	**Gemella_haemolysans**
Kingella oralis	**Kingella_oralis**

*In bold all species that are used for cluster analysis of gingivitis- and health associated bacteria in our study

2 Supplementary and Figures

Supplementary Figure S1: Sequencing depth of the samples included in the study.

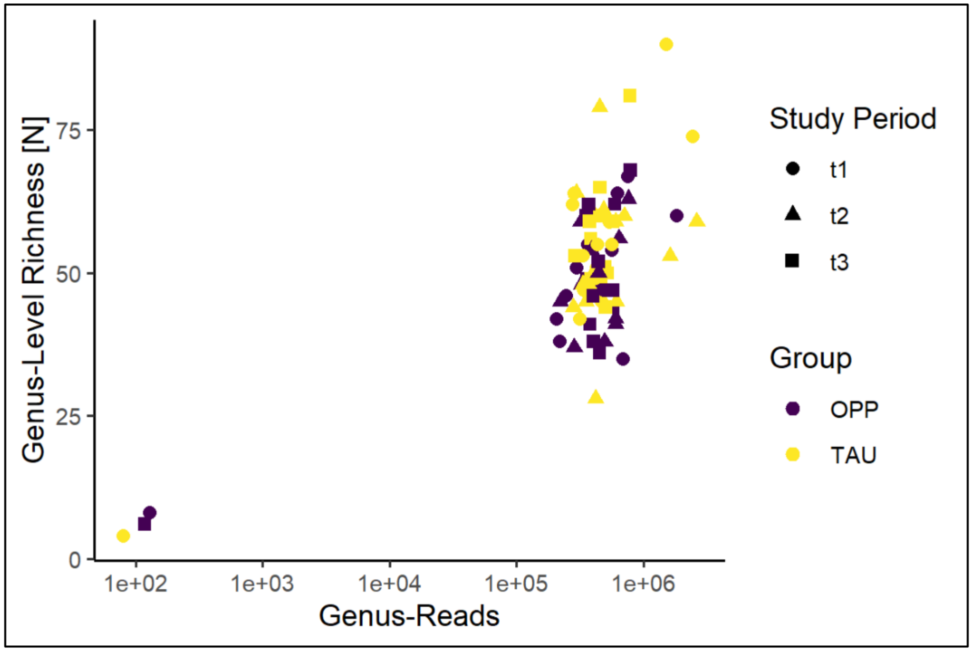

Abbreviations: OPP: Intensive oral prophylaxis program, TAU: Treatment-As-Usual

Supplementary Figure S2 (A-C): Average abundance of the most common phyla, genera and species in the two groups at basline (t0)

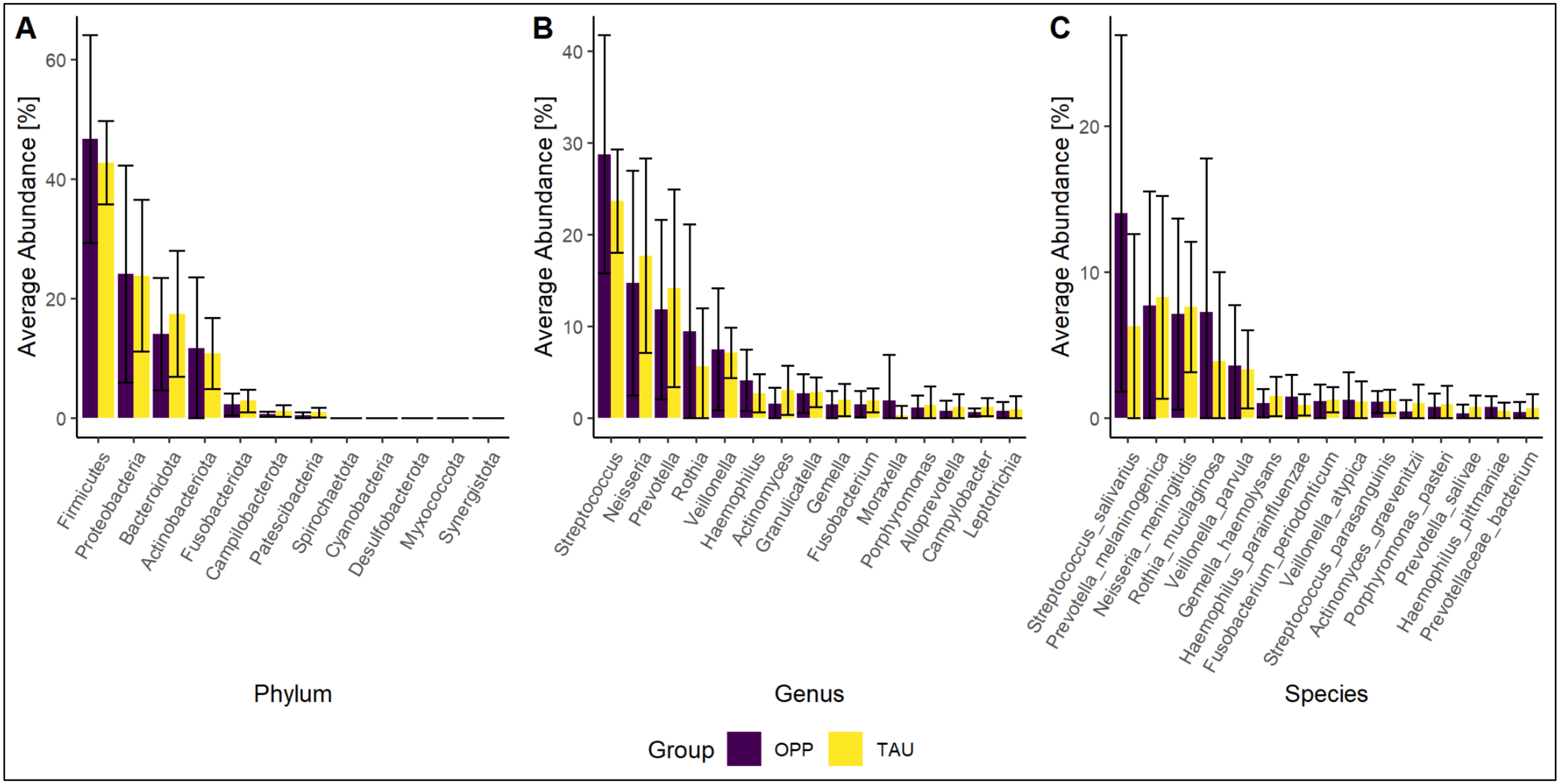

Abbreviations: OPP: Intensive oral prophylaxis program, TAU: Treatment-As-Usual

Supplementary Figure S3 (A-C): Comparison of alpha diversity (A), richness (B) and beta-diversity (C) at baseline (t1) between OPP and TAU group (genus level)

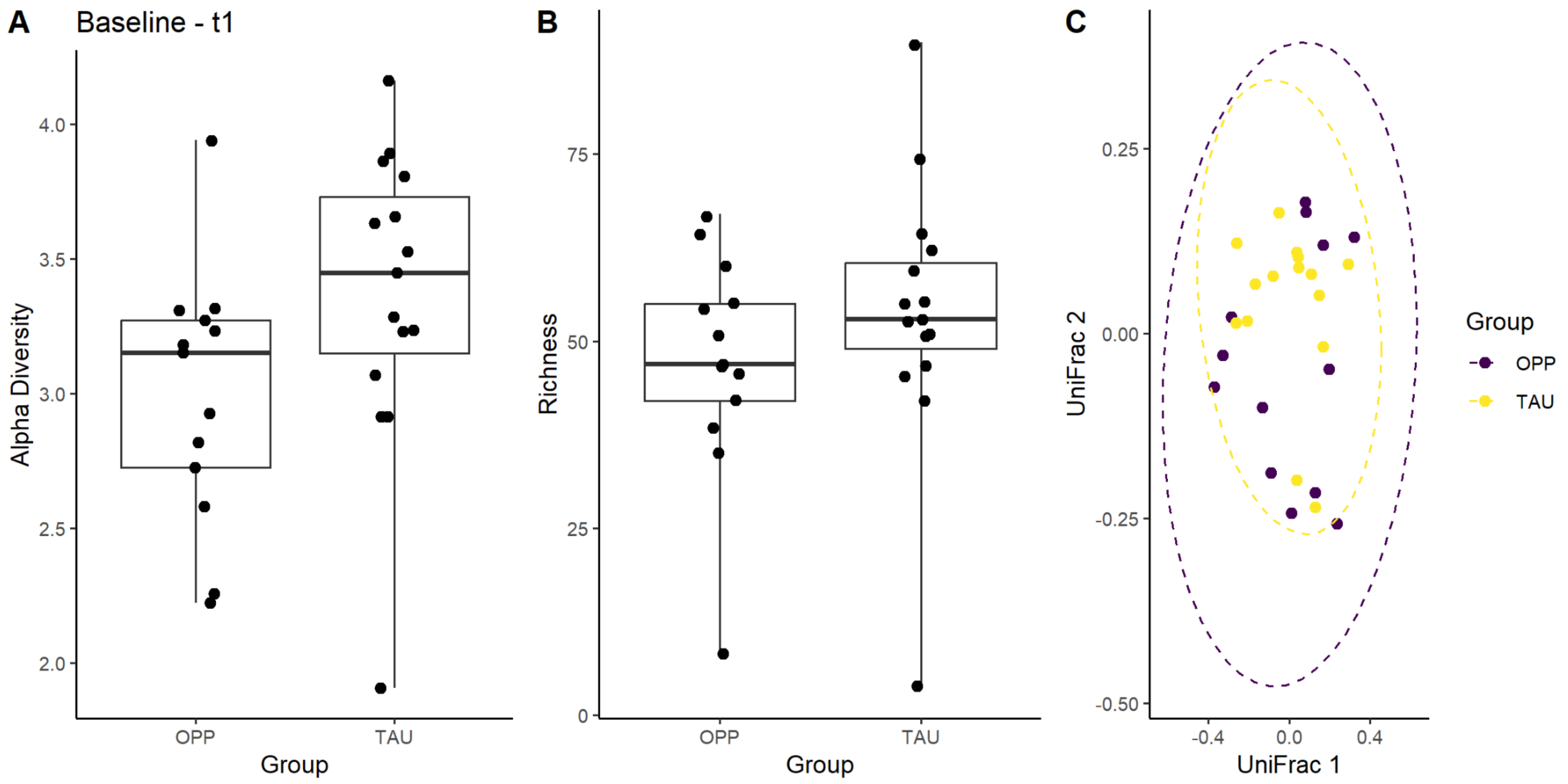

Abbreviations: OPP: Intensive oral prophylaxis program, TAU: Treatment-As-Usual
